# Design and Synthesis of 1-*O*- and 6′-*C*-Modified Heparan Sulfate Trisaccharides as Human Endo-6-*O*-Sulfatase 1 Inhibitors

**DOI:** 10.3389/fchem.2022.947475

**Published:** 2022-07-13

**Authors:** Kuei-Yao Tseng, Zheng-Hao Tzeng, Ting-Jen Rachel Cheng, Pi-Hui Liang, Shang-Cheng Hung

**Affiliations:** ^1^ School of Pharmacy, College of Medicine, National Taiwan University, Taipei, Taiwan; ^2^ Genomics Research Center, Academia Sinica, Taipei, Taiwan; ^3^ Department of Applied Science, National Taitung University, Taitung, Taiwan; ^4^ Department of Chemistry, National Cheng Kung University, Tainan, Taiwan

**Keywords:** heparan sulfate, glycosaminoglycans, endo-6-O-sulfatases, carbohydrate chemistry, inhibitors

## Abstract

The extracellular human endo-6-*O*-sulfatases (Sulf-1 and Sulf-2) are responsible for the endolytic cleavage of the 6-sulfate groups from the internal D-glucosamine residues in the highly sulfated subdomains of heparan sulfate proteoglycans. A trisaccharide sulfate, IdoA2OS-GlcNS6S-IdoA2OS, was identified as the minimal size of substrate for Sulf-1. In order to study the complex structure with Sulf-1 for developing potential drugs, two trisaccharide analogs, IdoA2OS-GlcNS6OSO_2_NH_2_-IdoA2OS-OMe and IdoA2OS-GlcNS6NS-IdoA2OS-OMe, were rationally designed and synthesized as the Sulf-1 inhibitors with IC_50_ values at 0.27 and 4.6 μM, respectively.

## Introduction

Heparan sulfate (HS) proteoglycans, which are ubiquitously distributed on the cell surface and in the extracellular matrix and basement membrane, play significant roles in adhesion, recognition, and signal transduction events (Vives et al., 2014). HS is a polyanionic polysaccharide belonging to the glycosaminoglycan (GAG) families, covalently bound to a core protein with a tetrasaccharide linkage region (El Masri et al., 2020). The HS backbone is composed of an alternative disaccharide repeating unit with all 1→4-linkages of *N*-acetyl-*α*-D-glucosamine (GlcNAc) and *β*-D-glucuronic acid (GlcA)/*α*-L-iduronic acid (IdoA). The acetyl group of GlcNAc could possibly be hydrolyzed to yield the amino group followed by *N*-sulfation, and/or the 2-*O* position of the uronic acid and/or the 3-*O* and/or 6-*O* positions of GlcN could undergo sulfation through a series of enzymatic modifications (Kreuger and Kjellén, 2012). The highly sulfated regions of HS, which were characterized as S-domains, are involved in binding with various proteins, such as fibroblast growth factors, transforming growth factor-*β*, Wnt, and bone morphogenetic protein (Li and Kusche-Gullberg, 2016). Changes in the HS sulfation patterns may cause dissociations with these proteins, resulting in up or downregulation of the corresponding signal transduction factors. Human endo-*O*-sulfatases 1 and 2 (Sulf-1 and Sulf-2) are two isoforms of the extracellular endo-6-*O*-sulfatases responsible for the hydrolysis of the sulfate groups at the 6-O positions of the internal D-glucosamine residues (Justo et al., 2022). Both enzymes modulate the sulfation patterns and regulate the HS-protein interactions (Hanson et al., 2004; El Masri et al., 2017). Numerous diseases have been proved to be related to the overexpression of Sulf-1 and Sulf-2, including gastric and pancreatic cancers, invasive breast carcinoma, lung adenocarcinoma, and osteoarthritis (OA) (Lai et al., 2003; Viviano et al., 2004; Otsuki et al., 2008; Otsuki et al., 2010; Rosen and Lemjabbar-Alaoui, 2010; Hur et al., 2012; Chanalaris et al., 2019; Lin et al., 2020; Severmann et al., 2020; Shamdani et al., 2020; Chivu-Economescu et al., 2022). Thus, the development of effective inhibitors may provide detailed information on their complex structures with Sulfs at the molecular level and offer an opportunity for structure-activity relationship for new drug discovery.

**FIGURE 1:**
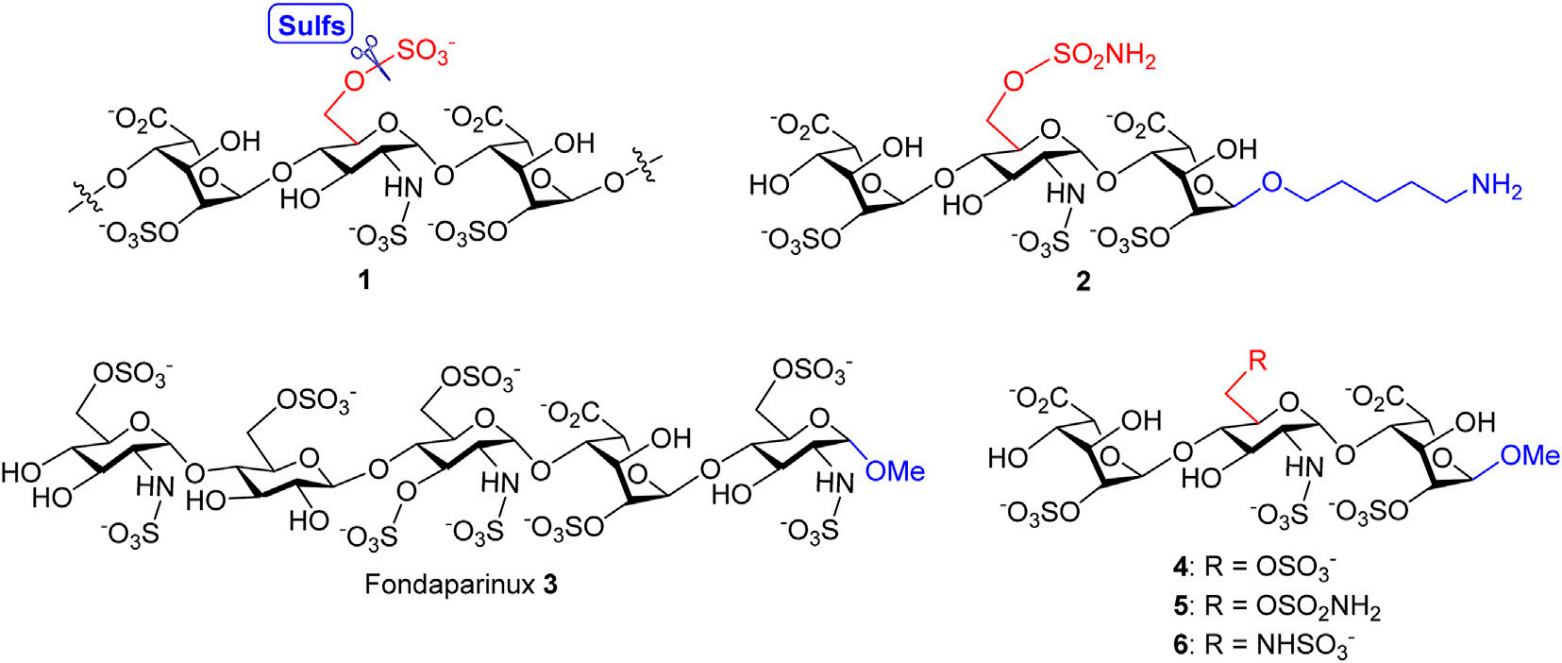
Structures of Sulf-1’s trisaccharide unit **1** and inhibitor **2** with a 5-amino-1-*n*-pentyl linker, the anticoagulant Fondaparinux **3**, and the proposed Sulf-1’s substrate **4** and inhibitors of the 6′-*O*-sulfonamide **5** and the 6′-*N*-sulfate **6** with the methyl group at 1-O.

In our previous study, a variety of HS oligosaccharides with different chain lengths and *N*- and *O*-sulfation patterns was screened for the substrate specificity of Sulf-1, and a trisaccharide sulfate, IdoA2OS-GlcNS6OS-IdoA2OS **1** (Figure 1), was identified as the minimal size of substrate for Sulf-1 (Chiu et al., 2020). A substrate analog **2** with the sulfonamide at the 6′-O position of GlcNS and a 5-amino-1-*n*-pentyl group at the 1-O position of IdoA had been developed as an inhibitor of Sulf-1. Although compound **2** could be immobilized on the chip of Surface Plasmon Resonance (SPR) for measurement of the dissociation constant (*K*_D_) with Sulf-1 or possibly be applied as a probe to identify new Sulfs *via* its attachment to the magnetic nanoparticles, the 5-amino-1-*n*-pentyl linker may cause the difficulty for crystallization of compound **2** with Sulf-1 for X-ray determination of the complex structure since its flexibility and the amino group might create an additional salt bridge with protein. Furthermore, the linker part may not be appropriate for the development of potential drug candidates. Inspired by the structure of Fondaparinux **3** (Chang et al., 2014), an anticoagulant drug used in the clinic, the methyl group is introduced to replace the 5-amino-1-*n*-pentyl group at the reducing end, and the substrate **4** and the 6′-*O*-sulfonamide inhibitor **5** are designed and prepared to test their activities for Sulf-1. Additionally, the 6′-*N*-sulfate **6** is synthesized for its interaction with Sulf-1 and its comparison with compounds **4** and **5**.

## Results and Discussion

**SCHEME 1:**
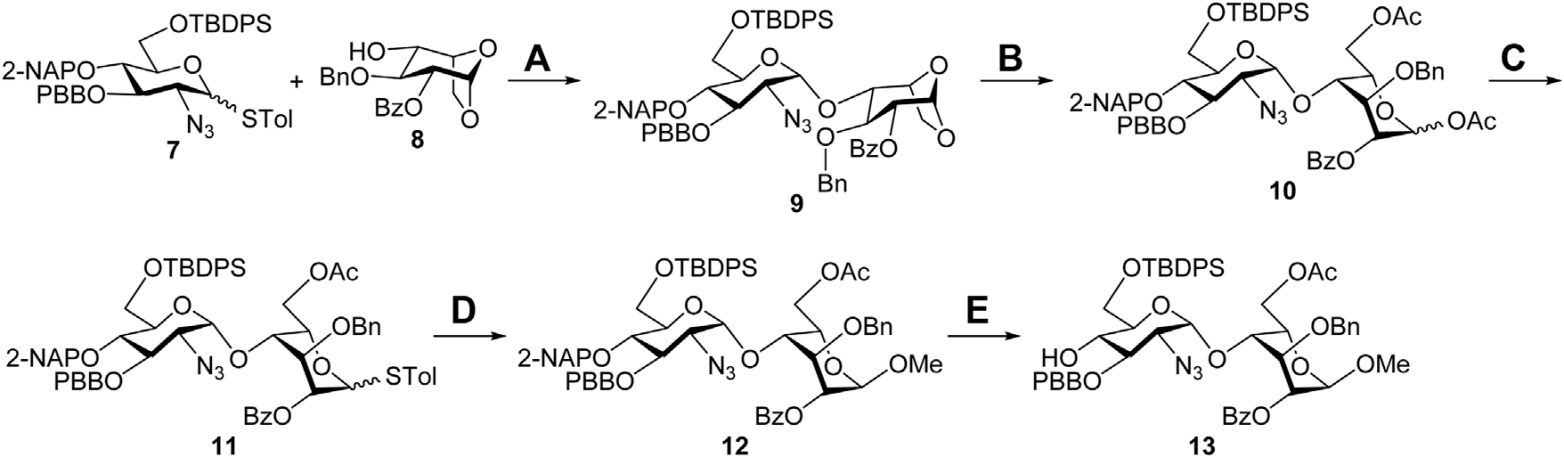
Synthesis of the disaccharide acceptor **13**. Reagents and conditions: (A) NIS, TfOH, CH_2_Cl_2_, 3 Å MS, −78°C to −20°C, 4 h, 71%; (B) Cu(OTf)_2_, Ac_2_O, RT, 1 h, 88%; (C) TMSSTol, ZnI_2_, CH_2_Cl_2_, 0°C to RT, 3 h, 76%; (D) NIS, TfOH, MeOH, CH_2_Cl_2_, 3 Å MS, −50°C, 2 h, 60%; (E) DDQ, CH_2_Cl_2_/H_2_O = 19/1, RT, 3 h, 79%. Ac, acetyl; Bn, benzyl; Bz, benzoyl; DDQ, 2,3-dichloro-5,6-dicyano-1,4-benzoquinone; 2-NAP, 2-naphthylmethyl; NIS, *N*-iodosuccinimide; PBB, *p*-bromobenzyl; TBDPS, *tert*-butyldiphenylsilyl; Tf, trifluoromethanesulfonyl; TMS, trimethylsilyl; Tol, 4-methylphenyl.

**Synthesis of the Sulf-1 substrate 4.** The backbone assembly of the target trisaccharide **4** is approached through a [1 + 2] strategy. The preparation of the disaccharide acceptor **13** is illustrated in Scheme 1. A coupling of the D-glucosamine-derived thioglycoside **7** with the 1,6-anhydro-*β*-L-idopyranosyl 4-alcohol **8** was carried out to yield the desired *α*-disaccharide **9** (71%), according to our previous report (Zulueta et al., 2012). Copper (II) trifluoromethanesulfonate-catalyzed acetolysis of compound **9** to open the 1,6-anhydro ring in acetic anhydride furnished the 1,6-diacetate **10** (88%), which underwent anomeric acetyloxy replacement *via* a combination of trimethylsilyl *p*-toluenyl thioether (TMSSTol) and zinc iodide (ZnI_2_) to give the corresponding thioglycoside **11** in 76% yield. Treatment of the disaccharide donor **11** with methanol in the presence of *N*-iodosuccinimide (NIS) and trifluoromethanesulfonic acid (TfOH) as the activators afforded the expected *α*-methyl disaccharide **12** (60%) because of neighboring group participation of the 2-*O*-benzoyl group. The stereochemistry was determined through a series of NMR spectral analyses, indicating the correlation of W-coupling between 1-H and 3-H in the 2D spectrum (Supplementary Material, Supplementary Pages S19–S22). Removal of the 2-naphthylmethyl (2-NAP) group at the 4′-O position of compound **12** using 2,3-dichloro-5,6-dicyano-1,4-benzoquinone (DDQ) provided the desired 4′-alcohol **13** in 79% yield.

**SCHEME 2:**
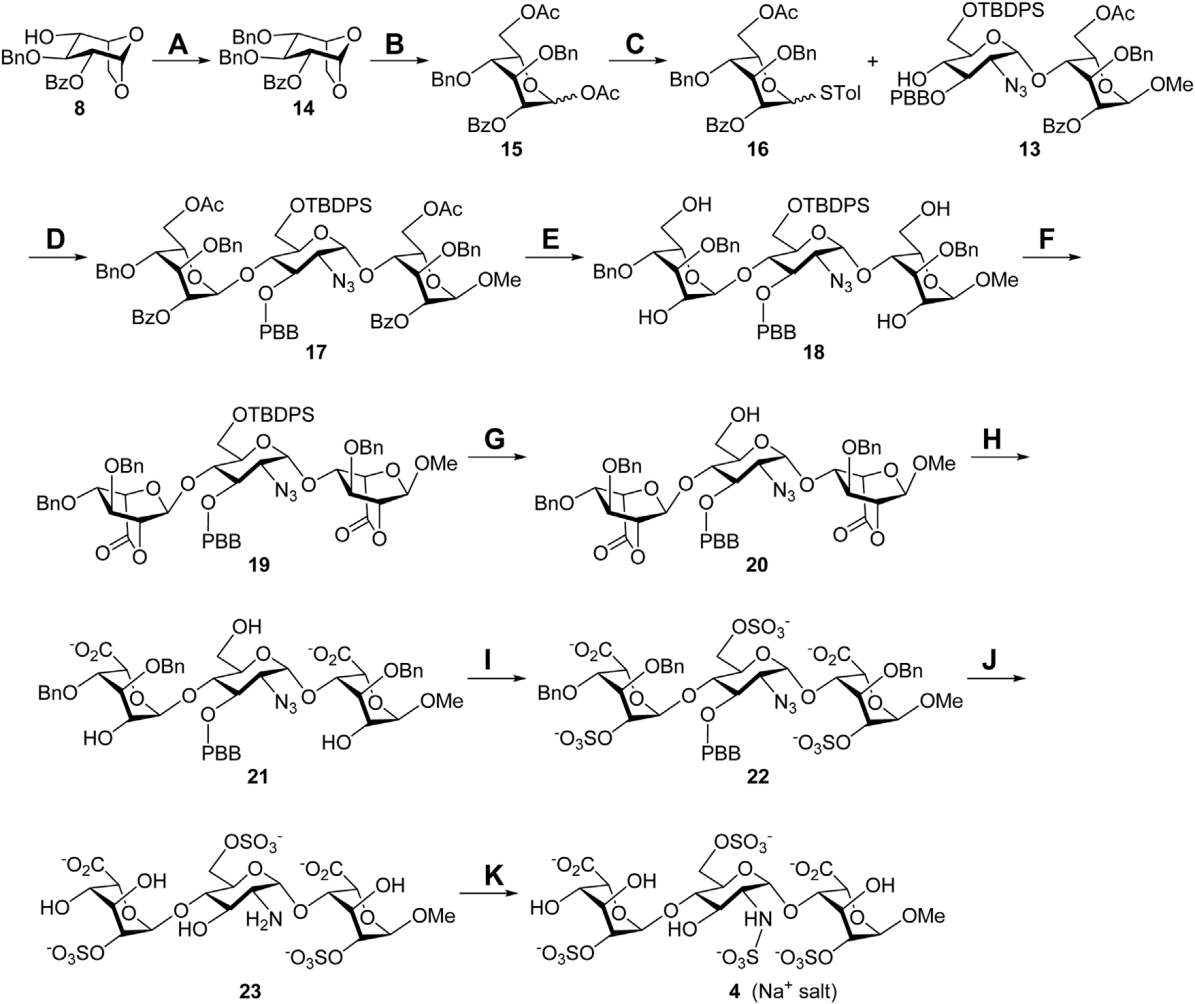
Synthesis of the Sulf-1 substrate **4**. Reagents and conditions: (A) BnBr, Ag_2_O, CH_2_Cl_2_, 3 Å MS, RT, 48 h, 94%; (B) Cu(OTf)_2_, Ac_2_O, RT, 1 h, 87%; (C) TMSSTol, ZnI_2_, CH_2_Cl_2_, 0°C to RT, 3 h, 74%; (D) NIS, TfOH, CH_2_Cl_2_, 3 Å MS, −40°C, 2 h, 71%; (E) NaOMe, CH_2_Cl_2_/MeOH = 1/1, 0°C to RT, 24 h, 86%; (F) TEMPO, BAIB, CH_2_Cl_2_/H_2_O = 2/1, RT, 6 h, 76%; (G) HF•pyridine, THF, 0°C to rt, 24 h, 78%; (H) 1 M LiOH_(aq)_, THF, RT, 1 h, 90%; (I) SO_3_•Et_3_N, DMF, 65°C, 24 h, 77%; (J) Pd(OH)_2_/C, H_2(g)_, phosphate buffer (pH = 7.0), MeOH, RT, 48 h, 90%; (K) SO_3_•pyridine, 1 M NaOH_(aq)_, H_2_O, RT, 48 h, 77%. BAIB, [bis(acetoxy)iodo]benzene; DMF, *N*,*N*-dimethylformide; TEMPO, (2,2,6,6-tetramethylpiperidin-1-yl)oxyl; THF, tetrahydrofuran.

With the glycosyl acceptor **13** in hand, the total synthesis of substrate **4** is depicted in Scheme 2. Benzylation of the 4-alcohol **8** (Ag_2_O, BnBr) led to the ether **14** (94%), which was opened under acetolysis conditions to generate the 1,6-diacetate **15** (87%). Anomeric substitution of compound **15** promoted by ZnI_2_ and TMSSTol was carried out, and the thioglycoside **16** was obtained in a 74% yield (Hu et al., 2011). Highly stereoselective *α*-glycosylation of the donor **16** with the acceptor **13** using NIS/TfOH delivered the desired trisaccharide **17** (71%), and all acyl groups in **17** were removed under Zemplén transesterification conditions to give the tetraol **18** (86%). Oxidation of **18** with (2,2,6,6-tetramethylpiperidin-1-yl)oxyl (TEMPO) and bis(acetoxy)iodobenzene (BAIB) furnished the dilactone **19** (76%), which was subjected upon cleavage of the *tert*-butyldiphenylsilyl (TBDPS) group with HF•pyridine complex at the 6′-O position yielding the 6′-alcohol **20** (78%). Opening of two lactone rings in compound **20** employing 1 M lithium hydroxide aqueous solution provided the 2,6′,2″-triol **21** (90%), which could be sulfonated with SO_3_•Et_3_N complex to afford the corresponding 2,6′,2″-tri-*O*-sulfate **22** (77%). Hydrogenolysis of compound **22** with Pd(OH)_2_/C and H_2_ in neutral buffer solution, allowing removal of the 1,3″,4″-tri-*O*-benzyl and 3′-*O*-*p*-bromobenzyl groups and reducing the 2′-*C*-azido group to the 2′-*C*-amino group, led to the desired product **23** (90%). *N*-Sulfonation of the amine **23** with SO_3_•pyridine complex at ca. pH 9.5, controlled by the addition of 1 M NaOH aqueous solution, furnished a crude mixture, which was purified through sequential Sephadex G10 size-exclusion column chromatography and Dowex 50WX8-Na^+^ ion-exchange column chromatography to yield target molecule **4** (Na^+^ salt, 77%). The structure of compound **4** was characterized through analyses of its 1D and 2D NMR spectra (Supplementary Material, Supplementary Pages S45–S49). The molecular weight of **4** (M+6Na^+^+H^+^, calculated for C_19_H_26_NO_29_S_4_Na_6_^+^ 997.8854, found 997.8815) was further confirmed by the high-resolution electrospray ionization mass spectrum (Supplementary Material, Supplementary Page S50).

**SCHEME 3:**
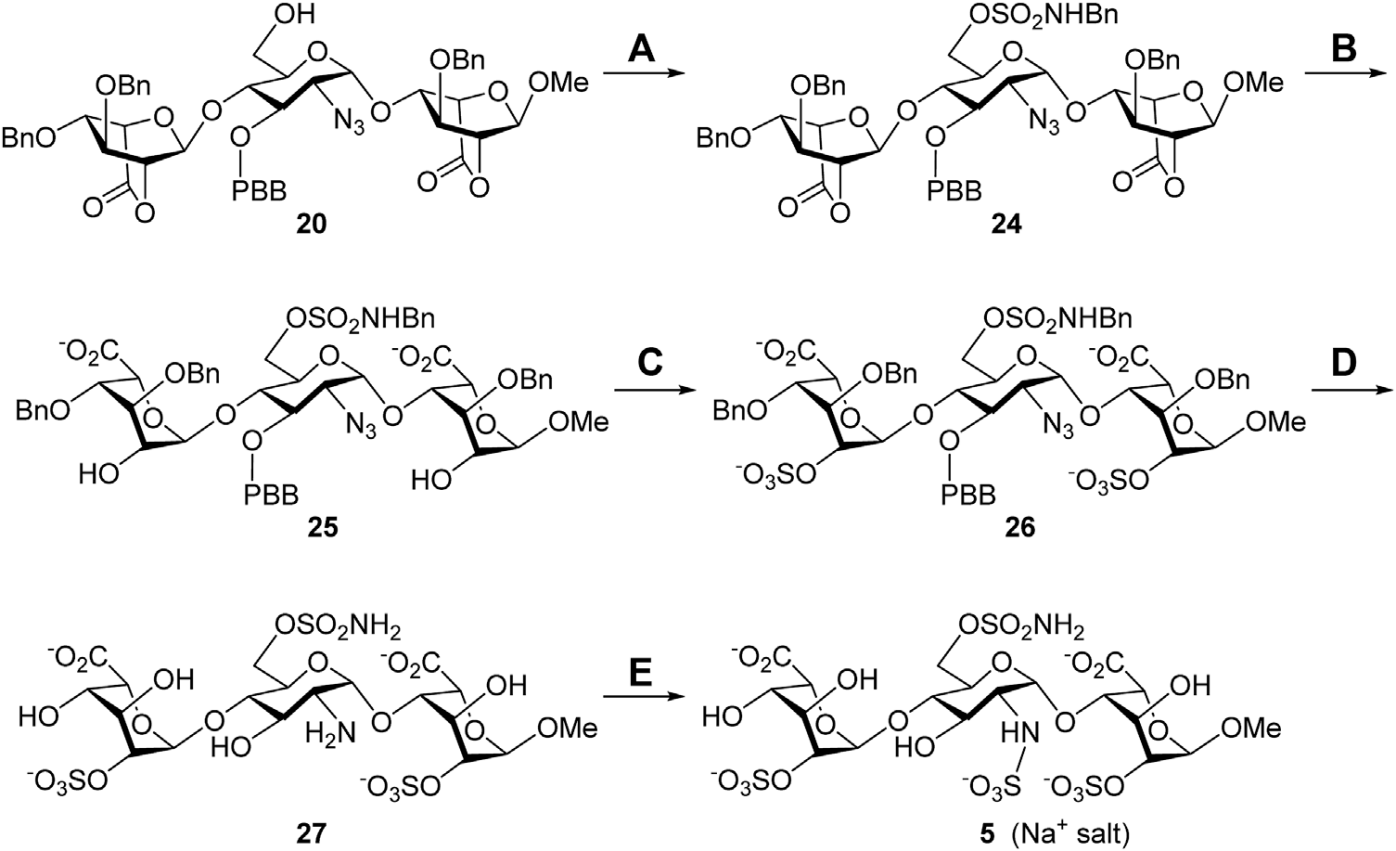
Synthesis of the 6′-*O*-sulfonamide trisaccharide inhibitor **5**. Reagents and conditions: (A) ClSO_2_NHBn, pyridine, RT, 15 min, 78%; (B) 1 M LiOH_(aq)_, THF, RT, 1 h, 90%; (C) SO_3_•Et_3_N, DMF, 65°C, 24 h, 70%; (D) Pd(OH)_2_/C, H_2(g)_, phosphate buffer (pH = 7.0), MeOH, RT, 48 h, 73%; (E) SO_3_•pyridine, 1 M NaOH_(aq)_, H_2_O, RT, 48 h, 75%.

**FIGURE 2:**
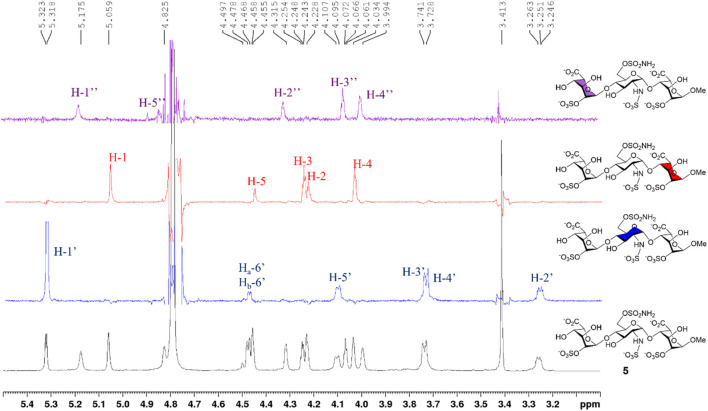
1D-TOCSY analysis of compound **5**.

**Synthesis of the 6′-*O*-sulfonamide trisaccharide inhibitor 5.** Scheme 3 describes the synthesis of HS trisaccharide analog **5** containing the 6′-*O*-sulfonamide as the Sulf-1 inhibitor. Starting from the common trisaccharide intermediate **20**, the 6′-hydroxy group was treated with *N*-benzyl sulfamoyl chloride and pyridine to give the *N*-benzyl-6′-*O*-sulfonamide derivative **24** (78%). It should be noted that the reaction needed to be quenched in 15 min since the NH-proton could be removed by the base and further coupled with *N*-benzyl sulfamoyl chloride to yield the unwanted side product when the reaction time is prolonged. The lactone rings of compound **24** were opened under basic conditions to provide the 2,2″-diol **25** (90%), which was converted into the corresponding di-*O*-sulfate **26** (70%) *via* sulfonation at the 2-O and 2″-O positions. Global deprotection of **26** under hydrogenolysis conditions led to the product **27** (73%), which underwent *N*-sulfonation to furnish the desired 6′-*O*-sulfonamide inhibitor **5** (Na^+^ salt) in 75% yield after consecutive purification through the size-exclusion column and ion-exchange column. The structure of compound **5** was determined *via* the 1D and 2D NMR spectral analyses (Supplementary Material, Supplementary Pages S57–S61). By using the trisaccharide **5** as a representative example, the assignments of all protons, including the splitting patterns and coupling constants, were examined by the 1D-Total Correlation Spectroscopy (1D-TOCSY) experiments in detail. As indicated in Figure 2, three isolated spectroscopic patterns were generated by 1H connectivity through *J*-coupling after the excitation of selective 1H nuclei at a given frequency (MacKinnon et al., 2016). The complicated proton NMR patterns of compound **5** could be resolved in three individual pyranosyl rings (purple one: non-reducing end L-iduronic acid sugar unit; blue one: D-glucosamine unit; red one: reducing end L-iduronic acid sugar unit) and characterized by combination with basic 1D and 2D NMR spectra. Starting from the 2D-HMBC spectrum, the correlation of ^3^J_C-H_ coupling between the anomeric carbon of the reducing end L-iduronic acid and the methyl protons at 1-O was identified. Two sets of ^2^J_C-H_ coupling correlations between the anomeric carbon of the L-iduronic acid and H-5 were also recognized. All of the anomeric hydrogen atoms could be illustrated through the ^1^J_C-H_ coupling between the corresponding anomeric carbon *via* the 2D-HSQC spectroscopy. In comparison with the 1D-TOCSY spectra, the red individual ^1^H spectrum was elucidated for the reducing end L-iduronic acid sugar unit *via* the corresponding H-1 and H-5. The purple individual ^1^H spectrum corresponded to the nonreducing end L-iduronic acid sugar unit through the H-5” correlation. The remaining blue individual ^1^H spectrum represented the D-glucosamine sugar unit. Finally, all proton peaks were assigned with the combination of 2D-COSY spectrum analysis. These techniques were applied to identify the structures of all compounds in this study. In addition, the high-resolution electrospray ionization mass spectrum further confirmed the correct molecular weight of **5** (M-6H^+^+5Na^+^, calculated for C_19_H_26_N_2_O_28_S_4_Na_5_^−^ 972.9049, found 972.9021) in comparison with the results of computational simulation (Supplementary Material, Supplementary Page S62).

**SCHEME 4:**
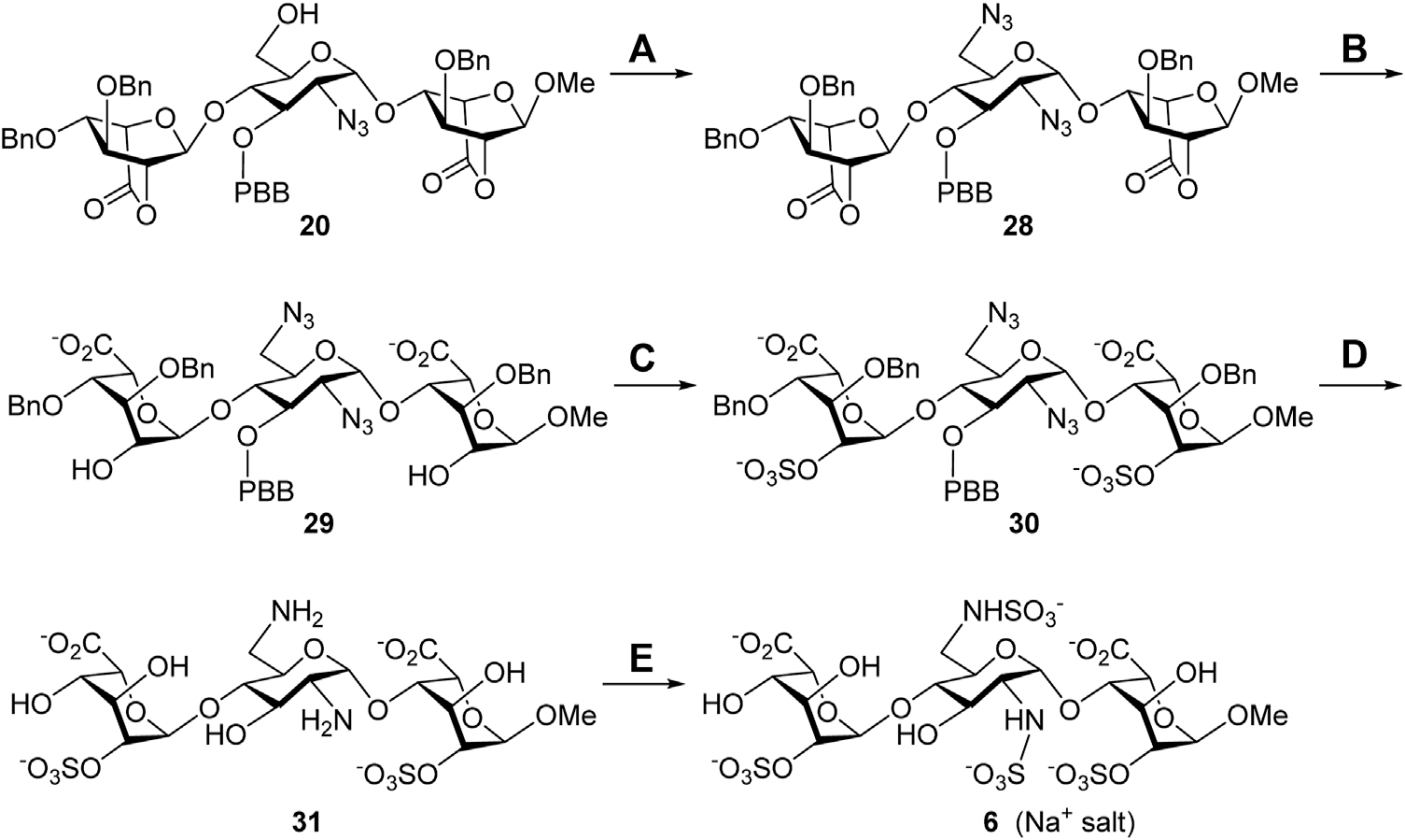
Synthesis of the 6′-*N*-sulfate trisaccharide inhibitor **5**. Reagents and conditions: (A) DPPA, DBU, toluene, 100°C, 24 h, 60%; (B) 1 M LiOH_(aq)_, THF, rt., 1 h, 86%; (C) SO_3_•Et_3_N, DMF, 65°C, 24 h, 86%; (D) Pd(OH)_2_/C, H_2(g)_, phosphate buffer (pH = 7.0), MeOH, rt., 48 h, 40%; (E) SO_3_•pyridine, 1 M NaOH_(aq)_, H_2_O, rt., 48 h, 78%. DBU, 1,8-diazabicyclo [5.4.0]undec-7-ene; DPPA, diphenylphosphoryl azide.

**Synthesis of the 6′-*N*-sulfate trisaccharide inhibitor 6.** The aforementioned common building block 6′-alcohol **20** was utilized to synthesize the 6′-*N*-sulfate derivative **6** (Scheme 4). The reaction of **20** with diphenylphosphoryl azide (DPPA) and 1,8-diazabicyclo [5.4.0]undec-7-ene (DBU) in toluene successfully transformed the 6′-hydroxy group into the 6′-azido group, and the expected 2′,6′-diazide **28** was obtained in 60% yield. Such harsh conditions, at 100°C for 24 h, were needed for the counterattack of the azide anion to the 6′-phosphate intermediate through S_N_2 nucleophilic substitution. With compound **28** ready, a similar four-step conversion was carried out to generate the final target molecule **6**, including the opening of two lactone rings (**29**, 86%), 2,2″-di-*O*-sulfonation (**30**, 86%), global deprotection by hydrogenolysis reduction, (**31**, 40%), and 2′,6′-di-*N*-sulfonation (**6**, 78%). Perhaps due to the chelation effect of the amino groups with the palladium catalyst causing difficult separation, the 2′,6′-diamine **31** was isolated in moderate yield. The structure of compound **6** was determined *via* the 1D and 2D NMR spectral analyses (Supplementary Material, Supplementary Pages S69–S73). The high-resolution electrospray ionization mass spectrum has confirmed the correct molecular weight of **6** (M + Na^+^, calculated for C_19_H_28_N_2_O_28_S_4_Na_5_^+^ 974.9194, found 974.9195) in comparison with the result of computational simulation (Supplementary Material, Supplementary Page S74).

**FIGURE 3:**
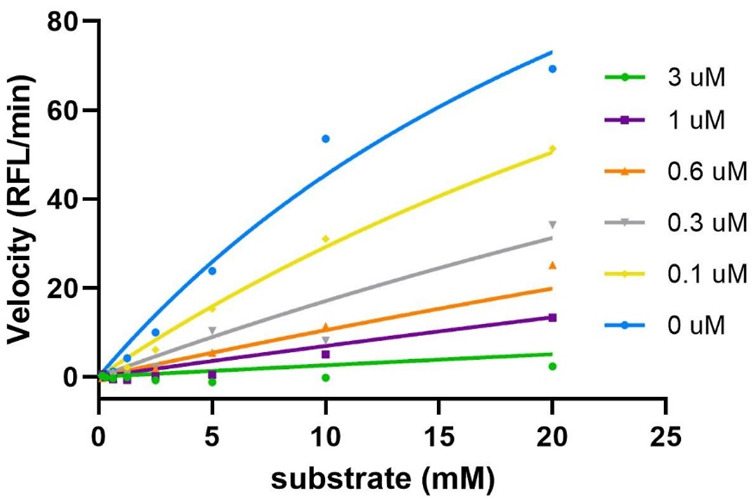
Kinetics studies of inhibition of compound 5. The enzyme activities were evaluated using various concentrations of substrate (0∼20 mM) and inhibitor (0∼3 uM). The results were then fitted into the competitive model using the equation *K*m, Obs = *K*m*(1+[I]/*K*i), and Y=Vmax*X/(*K*m, Obs + X) (GraphPad). The *K*i value was determined as 0.14 μM.

**Sulf-1 inhibition assay.** Human Sulf-1 was overexpressed and purified according to the procedure of the previous report (Chiu et al., 2020). The fluorescent assay was employed to measure the inhibitory activity by the released 4-methylumbelliferone (4-MU) from hydrolysis of 4-methylumbelliferyl sulfate (4-MUS) in the presence of human Sulf-1 and the inhibitor. Compounds **4**, **5**, and **6** were examined in these experiments. Since the methyl trisaccharide **4** exhibited similar substrate activity as **1** toward Sulf-1, it did not have any inhibition property (IC_50_ ˃ 100 μM). The IC_50_ value of the 6′-*O*-sulfonamide trisaccharide **5** was measured at 0.27 μM, and the kinetic studies of the inhibitory activity showed a competitive inhibition with the *K*i value of 0.14 μM (Figure 3). This indicated that replacement of the methyl moiety from 5-amino-1-*n*-pentyl at the 1-O position maintained a similar level of inhibitory activity to Sulf-1, suggesting that the 5-amino-pentyl group of **2** did not contribute to additional interaction with Sulf-1. Interestingly, the 6′-*N*-sulfate trisaccharide **6** with the IC_50_ value of 4.6 μM confirmed that it was an inhibitor to Sulf-1. Although the structures of compounds **4** and **6** were only one bioisosteric modification from *O*-sulfate to *N*-sulfate at the 6′-position, the interactions with Sulf-1 were different. These results have revealed that the sulfonamide group could be an appropriate moiety to inhibit Sulf-1 activity (Hanson et al., 2004). The *N*-sulfate moiety, albeit with lower inhibition, provided alternative considerations for the design of new inhibitors.

## Conclusion

The total syntheses of the 1-*O*- and 6′-*C*-modified HS trisaccharides **4**, **5**, and **6** *via* the 6′-alcohol **20** as the common intermediate have been successfully developed. Both bicyclo[2.2.2]-lactone rings in **20** block the 2,2″-dihydroxy groups, eliminating two protecting groups and allowing the functional group transformation at the 6′-hydroxy group. A straightforward four-step transformation, including lactone opening, *O*-sulfonation, hydrogenolysis, and *N*-sulfonation, has efficiently yielded the desired products **4**, **5**, and **6**. The 1-*O*-methyl-modified trisaccharide **4** has been identified as the Sulf-1 substrate that can be used to test the enzyme activity. In comparison with compound **2**, the 1-*O*-methyl-modified 6′-*O*-sulfonamide trisaccharide **5** exhibits similar inhibitory property (IC_50_ = 0.27 μM) that is an appropriate molecule to complex with Sulf-1 for further 3D structural studies by X-ray single crystal diffraction technique. Compound **5** is also a potent candidate for new drug discovery against diseases related to Sulf-1 overexpression. The 6′-*N*-sulfate trisaccharide **6**, which is not a Sulf-1 substrate, has been characterized as an inhibitor with IC_50_ = 4.6 μM for Sulf-1. The effectiveness of **5** to inhibit Sulf-1’s activity is 17 times better than **6**.

## Data Availability

The original contributions presented in the study are included in the article/Supplementary Material, further inquiries can be directed to the corresponding author.
