# Supplementary material for: Design and Synthesis of 1-O- and 6′-C-Modified Heparan Sulfate Trisaccharides as Human Endo-6-O-Sulfatase 1 Inhibitors
Source: Front Chem. 2022 Jul 13;10:947475. doi: 10.3389/fchem.2022.947475 (PMC9326219; doi:10.3389/fchem.2022.947475)
Supplement: Supplementary file 1 [file DataSheet1.PDF]

Supplementary Material

Design and Synthesis of 1-*O*- and 6'-*C*-Modified Heparan Sulfate Trisaccharides as Human Endo-6-*O*-sulfatase 1 Inhibitors

Kuei-Yao Tseng[1,2], Zheng-Hao Tzeng[2], Ting-Jen Rachel Cheng[2], Pi-Hui Liang[1,2]*, and Shang-Cheng Hung[2,3,4]*

[1]School of Pharmacy, College of Medicine, National Taiwan University, Taipei 10050, Taiwan
[2]Genomics Research Center, Academia Sinica, Taipei 11529, Taiwan
[3]Department of Applied Science, National Taitung University, Taitung 95092, Taiwan
[4]Department of Chemistry, National Cheng Kung University, Tainan 70101, Taiwan

Table of Contents

1. Inhibitory Activity Assay for Sulf-1…………………… ………….…………….……..S3
2. Chemical Synthesis and Data Characterization… ………….…………….……….……S3
3. General Information… …………..………………………….……..…………….…….……S3
4. General Procedures for *O*-Sulfonation, Hydrogenolysis, and *N*-Sulfonation……………S4
5. Experimental Procedures and Data Analyses ……………………...………….……..……S5
6. Synthesis of Compound **12**………………..……………………………………………. S5
7. Synthesis of Compound **13**………………..……………………………………………. S6
8. Synthesis of Compound **17**………………..……………………………………………. S7
9. Synthesis of Compound **18**………………..……………………………………………. S8
10. Synthesis of Compound **19**………………..……………………………………………. S9
11. Synthesis of Compound **20**………………..………………………………...………. S10
12. Synthesis of Compound **21**………………..………………………………...………. S11
13. Synthesis of Compound **4**………………..………………………………………………. S11
14. Synthesis of Compound **24**………………..……………………..………………. S12
15. Synthesis of Compound **25**………………..……………..…………………………. S13
16. Synthesis of Compound **5**………………..……………………………………………. S14
17. Synthesis of Compound **28**………………..…………………………..……………. S14
18. Synthesis of Compound **29**………………..……………..…………………………. S15
19. Synthesis of Compound **6**………………...……………………………………………. S16
20. References……………………………………………………...……….. ….…S17
21. NMR and HRMS data……..…………………… ……………………………………S18

1. Inhibitory Activity Assay for Sulf-1

Human Sulf-1 was overexpressed and purified according to the procedure reported previously.[1] The activity of Sulf-1 was determined by incubating 4-MUS (4.35 mM) with Sulf-1 in 50 mM Tris, 15 mM HEPES, 225 mM NaCl, 5 mM $CaCl_2$, and 5 mM $MgCl_2$ (pH 7.4) for 1 h at 37 °C, and the fluorescent intensity was measured at 460 nm following excitation at 355 nm in an ELISA reader (CLARIOstar plate reader).

2. Chemical Synthesis and Data Characterization

General Information. CH_2Cl_2 was purified and dried from a safe purification system filled with anhydrous Al_2O_3. All other reagents obtained from commercial sources were used without further purification. Water was either distilled or Milli-Q-purified. All the air/or moisture-sensitive reactions were carried out in an argon atmosphere with anhydrous solvents. Flash column chromatography was carried out on Silica Gel 60 (230–400 mesh, E. Merck). TLC was performed on glass plates pre-coated with Silica Gel 60 F_{254} (0.25 mm, E. Merck); detection was executed by spraying with a solution of $Ce(NH_4)_2(NO_3)_6$, $(NH_4)_6Mo_7O_{24}$, and H_2SO_4 in water and subsequent heating on a hot plate. Specific rotations were taken at ambient conditions and reported in 10^{-1}•deg•cm^2•g^{-1}; the sample concentrations are in g•dL^{-1}. ^{1}H and ^{13}C NMR spectra were recorded on 600 MHz spectrometers. Chemical shifts are in ppm from Me_4Si calibrated using the resonances of the carbon and the residual proton of the deuterated solvent. Proton peaks were assigned with the aid of 2D NMR techniques (^{1}H-^{1}H COSY, HMQC, and NOESY); the hydrogen multiplicities of carbon peaks were determined using DEPT experiments. Optical rotations were determined with HORIBA Sepa-300 Polarimeter by using 589 nm (sodium D line) at room temperature. IR spectra were used KBr plated and recorded by a Perkin-Elmer Paragon 1000 FTIR Spectrometer. Mass spectra were obtained with ESI Finnign LCQ mass spectrometer (Thermo Finnigan). Before all the glycosylation and one-pot reactions, the starting materials were dried under a high vacuum overnight in a desiccator.

3. General Procedures for *O*-Sulfonation, Hydrogenolysis, and *N*-Sulfonation

Method A: General procedure for *O*-sulfonation. A solution of the oligosaccharide (1 equiv.) and SO_3•Et_3N (3 equiv. per OH group of the oligosaccharide) in DMF (10 mL/g of oligosaccharide) was stirred at 60 °C for 16 h under nitrogen atmosphere. The reaction flask was cooled down to room temperature, and water and solid $NaHCO_3$ were added to the solution, and the resulting mixture was stirred for another 4 h. The whole mixture was concentrated *in vacuo*, and the residue was purified by silica gel (CH_2Cl_2/MeOH = 10/1→3/1) followed by Sephadex LH-20 with MeOH as eluent to yield the *O*-sulfonated compound.

Method B: General procedure for hydrogenolysis. A solution of the oligosaccharide and $Pd(OH)_2$/C (3 g/g of the oligosaccharide) in phosphate buffer (pH = 7.0, 15 mL/g of the oligosaccharide) was added appropriate amount of MeOH to dissolve the oligosaccharide at room temperature. The mixture was flushed with argon for 10 min, and the reaction flask was equipped with a hydrogen balloon. After stirring for 2 d. The whole mixture was filtered through Celite®, and the filtrate was concentrated *in vacuo*. The residue was purified through Sephadex G-10 column using water as the eluent, and the product portion was lyophilized to afford the desired amine.

Method C: General procedure for *N*-sulfonation. The oligosaccharide was dissolved in water (10 mL/g of oligosaccharide), and the solution was adjusted to pH 9.5 through the addition of 1.0 N $NaOH_{(aq)}$. SO_3•pyridine (25 equiv. per NH_2 group of the oligosaccharide) was added to the mixture in four equal portions at half-hour intervals at room temperature, while the pH value was maintained at 9.5 by careful addition of 1.0 N $NaOH_{(aq)}$. After stirring for 2 d, the reaction mixture was concentrated *in vacuo*, and the residue was purified by Sephadex G-10 column followed by AG 50W-X8 cation exchange column (Na^+ form) using water as the eluent. The product portion was lyophilized to give the *N*-sulfate (Na^+ salt).

4. Experimental Procedures and Data Analyses

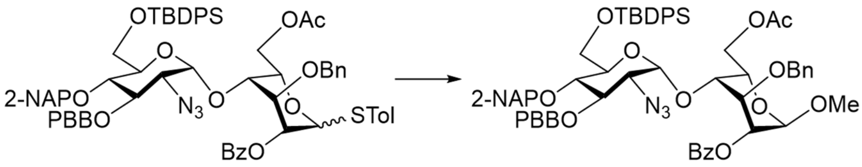

Compound 12. Flame-dried AW-300 molecular sieves (1.2 g) were added to a solution of Compound **11**[2] (1.23 g, 0.98 mmol) and NIS (0.33 g, 1.47 mmol) in CH_2Cl_2 (25 mL) at room temperature, and the mixture was stirred for 30 min under nitrogen atmosphere. The reaction flask was cooled down to –40 °C and kept stirring for 0.5 h. TfOH (87 μL, 0.98 mmol) was added dropwise to the reaction, and the mixture was kept stirring for 5 min. Methanol (47 μL, 1.18 mmol) was added dropwise to the reaction, and the solution was stirred for 2 h. The reaction was quenched with 10% $Na_2S_2O_{3(aq)}$ and sat. $NaHCO_{3(aq)}$. The reaction mixture was filtered through a pad of Celite® and washed with CH_2Cl_2. The combined organic layers were washed with water, dried over anhydrous $MgSO_4$, filtered, and concentrated *in vacuo*. The crude product was purified by flash column chromatography (hexane/ethyl acetate = 5/1) on silica gel to yield compound **12** (0.68 g, 60%) as a white solid. IR (thin film in KBr) ν 3054, 2930, 2858, 2109, 1744, 1720, 1601, 1489, 1453, 1428, 1366, 1317, 1269, 1237, 1112, 1071, 1042, 1012, 894, 855, 822, 743, 710, 614, 505 cm^{-1}; ^1H NMR (600 MHz, $CDCl_3$): δ 8.13-8.12 (m, 2H; Ar-H), 7.85-7.83 (m, 1H; Ar-H), 7.77-7.75 (m, 2H; Ar-H), 7.67-7.63 (m, 4H; Ar-H), 7.58 (s, 1H; Ar-H), 7.51-7.50 (m, 2H; Ar-H), 7.43-7.33 (m, 13H; Ar-H), 7.30-7.24 (m, 4H; Ar-H), 6.98 (d, J = 8.1 Hz, 2H; Ar-H), 5.10 (m, 1H; H-2), 4.92 (d, J = 11.8 Hz, 1H; Ar-CH_2), 4.88-4.82 (m, 3H; H-1, Ar-CH_2), 4.76 (d, J = 11.8 Hz, 1H; Ar-CH_2), 4.49 (d, J = 3.6 Hz, 1H; H-1'), 4.41-4.33 (m, 3H; H-5, H_a-6, Ar-CH_2), 4.13-4.07 (m, 2H; H_b-6, Ar-CH_2), 4.05 (s, 1H; H-3), 3.99 (d, J = 11.5 Hz, 1H; H_a-6'), 3.87 (d, J = 11.6 Hz, 1H; H_b-6'), 3.77-3.76 (m, 2H; H-4', H-5'), 3.68-3.65 (m, 1H; H-3'), 3.60 (m, 1H; H-4), 3.44 (s, 3H, OCH_3), 3.26 (dd, J = 7.1, 3.5 Hz, 1H; H-2'), 1.94 (s, 3H; $OCOCH_3$), 1.05 (s, 9H; *t*-Bu); ^{13}C NMR (150 MHz, $CDCl_3$): δ 170.5 (C), 165.7 (C), 137.7 (C), 136.7 (C), 135.9 (CH), 135.6 (CH), 135.4 (C), 133.5 (C), 133.3 (C), 133.2 (CH), 133.0 (C), 131.5 (CH), 130.0 (C), 129.9 (CH), 129.78 (CH), 129.76 (CH), 129.6 (CH), 128.6 (CH), 128.47 (CH), 128.46 (CH), 128.3 (CH), 128.1 (CH), 128.0 (CH), 127.8 (CH), 127.7 (CH), 126.4 (CH), 126.3 (CH), 126.1 (CH), 125.7 (CH), 121.8 (C), 99.5 (CH),

98.5 (CH), 80.7 (CH), 77.9 (CH), 75.2 (CH_2), 74.5 (CH), 74.4 (CH_2), 73.1 (CH), 72.7 (CH_2), 72.6 (CH), 69.0 (CH), 65.4 (CH), 64.0 (CH), 63.3 (CH_2), 62.3 (CH_2), 55.6 (CH_3), 26.9 (CH_3), 20.8 (CH_3), 19.4 (C); HRMS m/z (ESI, M+NH_4^+) calcd for $C_{63}H_{70}BrN_4O_{12}Si^+$ 1181.3937, found 1181.3937.

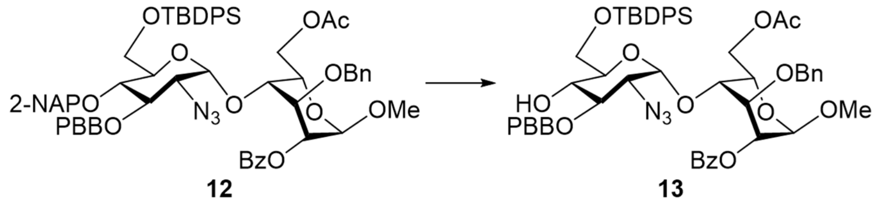

12 13

Compound 13. Compound **12** (1.54 g, 1.32 mmol) was treated with DDQ (0.60 g, 2.64 mmol), in two equal portions in half-hour intervals in a mixed solvent (CH_2Cl_2/H_2O = 19/1, 77 mL) at room temperature. After stirring for 3 h, the reaction mixture was quenched with 10% $Na_2S_2O_{4(aq)}$ and filtered through a pad of Celite®. The filtrate was concentrated *in vacuo*, and the residue was purified by flash column chromatography (hexane/ethyl acetate = 4/1) on silica gel to afford the desired disaccharide **13** (1.07 g, 79%) as a white solid. IR (thin film in KBr) ν 3497, 3070, 2931, 2858, 2109, 1743, 1719, 1600, 1489, 1453, 1428, 1367, 1317, 1268, 1113, 1070, 1043, 1013, 823, 804, 743, 709, 614, 505 cm^{-1}; ^1H NMR (600 MHz, $CDCl_3$): δ 8.09 (d, *J* = 7.3 Hz, 2H; Ar-H), 7.62-7.60 (m, 4H; Ar-H), 7.43-7.32 (m, 13H; Ar-H), 7.27-7.25 (m, 2H; Ar-H), 7.21-7.19 (m, 1H; Ar-H), 7.06-7.05 (m, 2H; Ar-H), 5.07 (m, 1H; H-2), 4.85-4.82 (m, 3H; H-1, Ar-CH_2), 4.69 (d, *J* = 11.8 Hz, 1H; Ar-CH_2), 4.48-4.45 (m, 2H; H-1', Ar-CH_2), 4.37-4.31 (m, 2H; H-5, H_a-6), 4.17-4.13 (m, 2H; H_b-6, Ar-CH_2), 3.98 (s, 1H; H-3), 3.84 (d, *J* = 10.9 Hz, 1H; H_a-6'), 3.75 (d, *J* = 10.6 Hz, 1H; H_b-6'), 3.66-3.57 (m, 3H; H-4, H-4', H-5'), 3.52-3.49 (m, 1H; H-3'), 3.41 (s, 3H, OCH_3), 3.10 (d, *J* = 10.0 Hz, 1H; H-2'), 2.61 (s, 1H; OH), 1.96 (s, 3H; $OCOCH_3$), 1.00 (s, 9H; *t*-Bu); ^{13}C NMR (150 MHz, $CDCl_3$): δ 170.5 (C), 165.9 (C), 137.5 (C), 137.0 (C), 135.6 (CH), 135.5 (CH), 133.2 (CH), 132.8 (C), 132.7 (C), 131.5 (CH), 129.97 (CH), 129.96 (CH), 129.91 (C), 129.86 (CH), 129.7 (CH), 128.5 (CH), 128.4 (CH), 128.0 (CH), 127.9 (CH), 127.8 (CH), 121.7 (C), 99.5 (CH), 98.0 (CH), 80.2 (CH), 74.2 (CH_2), 74.0 (CH), 72.6 (CH), 72.4 (CH), 71.9 (CH), 69.2 (CH), 65.2 (CH), 64.1 (CH_2), 63.3 (CH_2), 63.1 (CH), 55.6 (CH_3), 26.7 (CH_3), 20.8 (CH_3), 19.2 (C); HRMS m/z (ESI, M+NH_4^+) calcd for $C_{52}H_{62}BrN_4O_{12}Si^+$ 1041.3311, found 1041.3308.

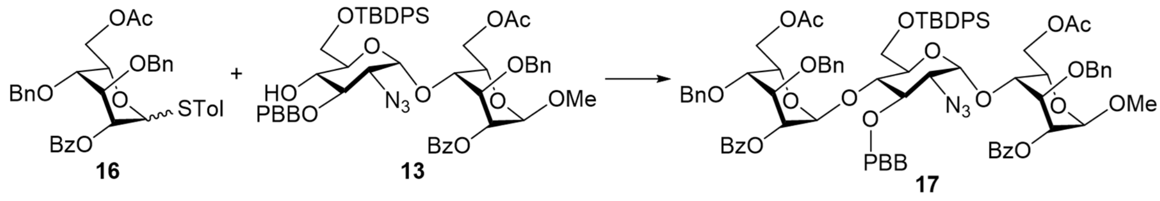

Compound 17. A mixture of the donor **16**[3] (0.98 g, 1.60 mmol), the acceptor **13** (1.37 g, 1.34 mmol), NIS (91 mg, 0.4 mmol) and flame-dried AW-500 molecular sieves (2.2 g) in anhydrous CH_2Cl_2 (50 mL) were stirred at room temperature for 30 min under nitrogen atmosphere. The reaction flask was cooled down to –40 °C, and the solution was kept stirring for 30 min. TfOH (35 μL, 0.40 mmol) was added to the mixture, and the resulting solution was stirred for 2 h. The reaction was quenched by addition of 10% $Na_2S_2O_{3(aq)}$ and sat. $NaHCO_{3(aq)}$. The crude reaction mixture was filtered through a pad of Celite® followed by wash with CH_2Cl_2. The combined organic layers were washed with water, dried over anhydrous $MgSO_4$, filtered, and concentrated *in vacuo*. The crude residue was purified by flash column chromatography (hexane/ethyl acetate = 5/1) on silica gel to get compound **17** (1.44 g, 71%) as a white solid. IR (thin film in KBr) ν 3066, 3031, 2931, 2858, 2110, 1743, 1719, 1601, 1489, 1453, 1428, 1390, 1369, 1317, 1269, 1243, 1159, 1113, 1071, 1042, 1028, 876, 823, 804, 753, 711, 701, 613, 506, 488 cm^{-1}; ^{1}H NMR (600 MHz, $CDCl_3$): δ 8.10-8.08 (m, 2H; Ar-H), 7.89 (d, J = 7.4 Hz, 2H; Ar-H), 7.61-7.60 (m, 2H; Ar-H), 7.54-7.50 (m, 3H; Ar-H), 7.39-7.27 (m, 20H; Ar-H), 7.24-7.22 (m, 4H; Ar-H), 7.13-7.10 (m, 4H; Ar-H), 6.89 (d, J = 8.2 Hz, 2H; Ar-H), 5.30 (s, 1H; H-1”), 5.20 (s, 1H; H-2”), 5.08 (m, 1H; H-2), 4.89-4.87 (m, 2H; H-1, Ar-CH_2), 4.81 (d, J = 11.5 Hz, 1H; Ar-CH_2), 4.71 (d, J = 11.8 Hz, 1H; Ar-CH_2), 4.61 (d, J = 11.4 Hz, 1H; Ar-CH_2), 4.56 (d, J = 11.4 Hz, 1H; Ar-CH_2), 4.47 (d, J = 11.5 Hz, 1H; Ar-CH_2), 4.37-4.33 (m, 2H; H_a-6, H_a-6”), 4.29-4.27 (m, 3H; H-1’, H-5, H-5”), 4.08-4.02 (m, 3H; H-4’, H_b-6, H_b-6”), 3.99-3.96 (m, 2H; H-3, Ar-CH_2), 3.93-3.87 (m, 3H; H_a-6’, H_b-6’, H-3”), 3.74 (d, J = 11.9 Hz, 1H; Ar-CH_2), 3.59 (d, J = 9.7 Hz, 1H; H-5’), 3.52-3.48 (m, 1H; H-3’), 3.46-3.45 (m, 1H; H-4), 3.43 (s, 3H; OCH_3), 3.34 (s, 1H; H-4”), 3.14 (dd, J = 10.3, 3.8 Hz, 1H; H-2’), 1.88 (s, 3H; $OCOCH_3$), 1.79 (s, 3H; $OCOCH_3$), 0.95 (s, 9H; *t*-Bu); ^{13}C NMR (150 MHz, $CDCl_3$): δ 170.4 (C), 170.3 (C), 165.8 (C), 165.7 (C), 137.59 (C), 137.56 (C), 137.2 (C), 136.0 (CH), 135.6 (CH), 133.5 (C), 133.3 (CH), 133.2 (CH), 131.0 (CH), 130.0 (C), 129.9 (C), 129.8 (CH), 129.7 (CH), 129.6 (CH), 129.58 (C), 129.50 (CH), 129.1 (CH), 128.94 (CH), 128.58 (CH), 128.56 (CH), 128.50 (CH), 128.44 (CH), 128.42 (CH), 128.39 (CH), 128.38 (CH), 128.30 (CH), 128.2 (CH), 128.1

(CH), 128.0 (CH), 127.9 (CH), 127.8 (CH), 127.5 (CH), 127.4 (CH), 125.4 (CH), 121.0 (C), 99.5 (CH), 97.5 (CH), 96.6 (CH), 79.1 (CH), 74.0 (CH_2), 73.7 (CH), 73.2 (CH), 73.0 (CH), 72.7 (CH_2), 72.6 (CH_2), 72.5 (CH), 72.3 (CH), 72.1 (CH_2), 71.9 (CH), 69.2 (CH), 68.1 (CH), 65.3 (CH), 65.2 (CH), 64.4 (CH), 63.0 (CH_2), 62.5 (CH_2), 62.2 (CH_2), 55.6 (CH_3), 26.8 (CH_3), 20.8 (CH_3), 20.7 (CH_3), 19.5 (C); HRMS m/z (ESI, M+NH_4^+) calcd for $C_{81}H_{90}BrN_4O_{19}Si^+$ 1529.5146, found 1529.5158.

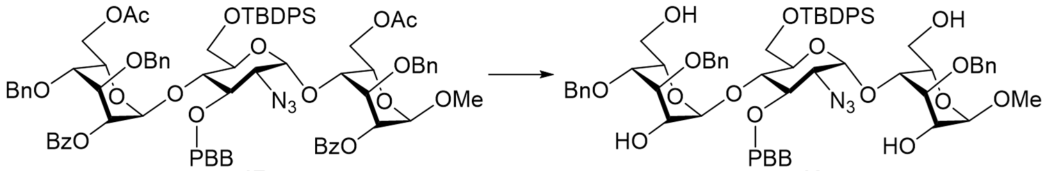

Compound 18. A solution of compound **17** (0.88 g, 0.58 mmol) in a mixed solvent (MeOH/CH_2Cl_2 = 1/1, 26 mL) was immersed in an ice bath under nitrogen atmosphere, $NaOCH_3$ (1.43g, 26.0 mmol) was added to the solution. The reaction flask was gradually warmed up to room temperature, and the solution was kept stirring for 24 h. The reaction was quenched by DOWEX 50 (H^+ exchange resin), the mixture was filtered off, and the solvent was removed under reduced pressure. The crude compound was dissolved in ethyl acetate followed by wash with water and brine. The organic solution was dried over anhydrous $MgSO_4$, filtered, and concentrated *in vacuo*. The residue was purified by flash column chromatography (hexane/ethyl acetate = 1.5/1) on silica gel to give compound **18** (0.61 g, 86%) as a white solid. IR (thin film in KBr) ν 3502, 3031, 2932, 2113, 1590, 1489, 1455, 1428, 1361, 1320, 1258, 1216, 1151, 1103, 1070, 1029, 845, 823, 806, 753, 701, 615, 505 cm^{-1}; ^{1}H NMR (600 MHz, $CDCl_3$): δ 7.71-7.69 (m, 5H; Ar-H), 7.42-7.40 (m, 5H; Ar-H), 7.38-7.31 (m, 14H; Ar-H), 7.26-7.25 (m, 2H; Ar-H), 7.06 (d, J = 8.1 Hz, 2H; Ar-H), 5.06 (s, 1H; H-1''), 4.83 (d, J = 3.7 Hz, 1H, H-1'), 4.81 (s, 1H, H-1), 4.73 (d, J = 12.0 Hz, 1H; Ar-CH_2), 4.66-4.62 (m, 3H; Ar-CH_2), 4.58 (d, J = 12.0 Hz, 1H; Ar-CH_2), 4.53 (d, J = 11.0 Hz, 1H; Ar-CH_2), 4.49 (d, J = 11.2 Hz, 1H; Ar-CH_2), 4.45 (d, J = 11.5 Hz, 1H; Ar-CH_2), 4.29-4.27 (m, 1H; H-4), 4.04-4.02 (m, 1H; H-5'), 3.98-3.94 (m, 1H; OH), 3.89-3.81 (m, 4H; H-5, H_a-6, H_a-6'', OH), 3.79-3.77 (m, 3H; H-2, H-5'', OH), 3.74-3.65 (m, 5H; H-2'', H-3, H-3', H-4', OH), 3.61 (s, 1H; H_b-6), 3.56-3.52 (m, 2H; H-2', H_b-6''), 3.45 (s, 3H; OCH_3), 3.44-3.41 (m, 1H; H_a-6'), 3.38 (s, 1H; H-4''), 3.28-3.26 (m, 1H; H-3''), 3.18-3.16 (m, 1H; H_b-6'), 1.07 (s, 9H; *t*-Bu); ^{13}C NMR (150 MHz, $CDCl_3$): δ 137.7 (C), 137.5 (C), 136.8 (C), 136.7 (C), 135.9 (CH), 135.8 (CH), 133.1 (C), 133.0 (C), 131.4 (CH),

129.9 (CH), 129.8 (CH), 129.1 (CH), 128.7 (CH), 128,64 (CH), 128.60 (CH), 128.4 (CH), 128.39 (CH). 128.36 (CH), 128.34 (CH), 128.1 (CH), 127.9 (CH), 127.8 (CH), 127.7 (CH), 121.6 (C), 102.4 (CH), 100.2 (CH), 94.8 (CH), 79.9 (CH), 74.9 (CH), 74.4 (CH_2), 73.5 (CH), 73.4 (CH), 72.9 (CH_2), 72.4 (CH_2), 71.8 (CH_2), 71.4 (CH), 70.6 (CH), 67.4 (CH), 67.3 (CH), 66.7 (CH), 66.4 (CH), 64.3 (CH), 62.5 (CH_2), 62.4 (CH_2), 62.0 (CH_2), 55.7 (CH_3), 26.9 (CH_3), 19.4 (C); HRMS m/z (ESI, M+NH_4^+) calcd for $C_{63}H_{78}BrN_4O_{15}Si^+$ 1237.4411, found 1237.4426.

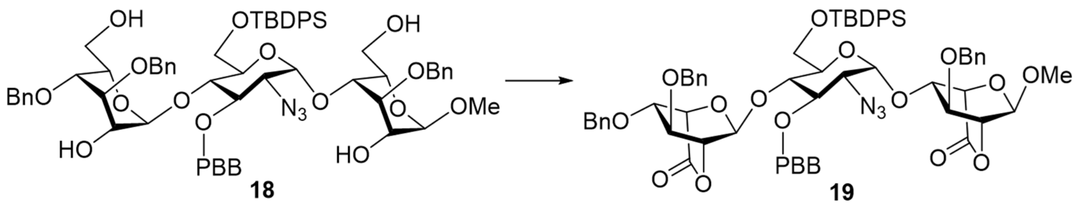

Compound 19. Compound **18** (0.52 g, 0.43 mmol) was dissolved in a mixed solvent (CH_2Cl_2/H_2O = 2/1, 6 mL) at room temperature, TEMPO (13 mg, 0.09 mmol) and BAIB (0.69 g, 2.15 mmol) were added to the mixture, and the reaction solution was stirred for 6 h. The reaction was quenched with 10% $Na_2S_2O_{3(aq)}$, and the aqueous layer was extracted with ethyl acetate (3 x 10 mL). The combined organic layers were washed with brine, dried over anhydrous $MgSO_4$, filtered, and concentrated *in vacuo*. The residue was purified by flash column chromatography (hexane/ethyl acetate = 4/1) on silica gel to provide compound **19** (0.40 g, 76%) as a white solid. IR (thin film in KBr) ν 3032, 2931, 2109 cm^{-1}; ^1H NMR (600 MHz, $CDCl_3$): δ 7.67-7.63 (m, 4H; Ar-H), 7.45-7.43 (m, 4H; Ar-H), 7.41-7.33 (m, 10H; Ar-H), 7.30-7.27 (m, 4H; Ar-H), 7.25-7.24 (m, 3H; Ar-H), 7.20-7.16 (m,4H; Ar-H), 5.45 (s, 1H; H-1”), 5.02-5.01 (m, 2H; H-1, H-1’), 4.87 (d, J = 11.4 Hz, 1H; Ar-CH_2), 4.74 (ABq, J = 12.1 Hz, 2H; Ar-CH_2), 4.57-4.55 (m, 2H; H-2, Ar-CH_2), 4.44-4.38 (m, 3H; Ar-CH_2), 4.30 (d, J = 3.2, 1H; H-5), 4.28 (s, 1H; H-2”), 4.24-4.22 (m, 3H; H-4, H-4”, Ar-CH_2), 3.99 (t, J = 9.5 Hz, 1H; H-4’), 3.88 (d, J = 12.0, 1H, H_a-6’), 3.85-3.84 (m, 1H; H-3), 3.80-3.79 (m, 2H; H-3”, H-5”), 3.72-3.66 (m, 3H; H-3’, H-5’, H_b-6’), 3.54 (s, 3H; OCH_3), 3.34 (dd, J = 10.4, 3.8 Hz, 1H; H-2’), 1.05 (s, 9H; *t*-Bu); ^{13}C NMR (150 MHz, $CDCl_3$): δ 167.6 (C), 167.3 (C), 137.2 (C), 137.0 (C), 136.9 (C), 136.8 (C), 135.9 (CH), 135.7 (CH), 132.9 (C), 132.7 (C), 131.5 (CH), 130.1 (CH), 129.9 (CH), 129.2 (CH), 128.63 (CH), 128.60 (CH), 128.53 (CH), 128.28 (CH), 128.24 (CH), 128.1 (CH), 128.07 (CH), 128.03 (CH), 127.76 (CH), 128.71 (CH), 121.6 (C), 99.8 (CH), 98.3 (CH), 98.1 (CH), 80.6 (CH), 79.5 (CH), 79.0 (CH), 77.9 (CH), 77.8 (CH), 77.7 (CH), 74.0

(CH$_2$), 72.5 (CH), 72.34 (CH$_2$), 72.33 (CH$_2$), 72.2 (CH), 72.0 (CH), 71.65 (CH$_2$), 69.5 (CH), 68.6 (CH), 63.2 (CH), 61.7 (CH$_2$), 57.3 (CH$_3$), 27.0 (CH$_3$), 19.3 (C); HRMS m/z (ESI, M+NH$_4^+$) calcd for $C_{63}H_{70}BrN_4O_{15}Si^+$ 1229.3785, found 1229.3795.

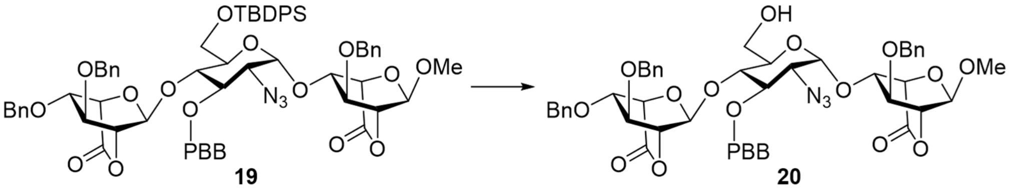

Compound 20. HF•pyridine (5.2 mL) was slowly added to a solution of compound **19** (0.70 g, 0.58 mmol) in THF (7 mL) at 0 °C under nitrogen atmosphere, and the reaction flask was gradually warmed up to room temperature for 24 h. The reaction was quenched with sat. $NaHCO_{3(aq)}$, and the aqueous layer was extracted by ethyl acetate (3 x 10 mL). The combined organic layers were washed with brine, dried over anhydrous $MgSO_4$, filtered, and concentrated *in vacuo*. The residue was purified by flash column chromatography (hexane/ethyl acetate = 1.5/1) on silica gel to furnish compound **20** (0.44 g, 78%) as a white solid. IR (thin film in KBr) ν 3515, 3031, 2932, 2110, 1790, 594, 1489, 1455, 1368, 1335, 1318, 1260, 1207, 1157, 1126, 1081, 1030, 911, 870, 806, 751, 698, 591, 555, 479 cm^{-1}; ^{1}H NMR (600 MHz, CDCl$_3$): δ 7.48-7.46 (m, 2H; Ar-H), 7.44-7.43 (m, 2H; Ar-H), 7.41-7.37 (m, 5H; Ar-H), 7.36-7.34 (m, 3H; Ar-H), 7.33-7.29 (m, 3H; Ar-H), 7.25-7.24 (m, 2H; Ar-H), 7.20 (d, J = 8.3 Hz, 2H; Ar-H), 5.38 (s, 1H; H-1''), 5.08-5.07 (m, 2H; H-1, H-1'), 4.91 (d, J = 11.3 Hz, 1H; Ar-CH$_2$), 4.78 (ABq, J = 12.1 Hz, 2H; Ar-CH$_2$), 4.65-4.55 (m, 6H; H-2, H-2'', Ar-CH$_2$), 4.45 (d, J = 11.8 Hz, 1H; Ar-CH$_2$), 4.30-4.24 (m, 3H; H-4'', H-5, H-5''), 3.96-3.86 (m, 4H; H-3, H-3'', H-4, H$_a$-6'), 3.83-3.75 (m, 3H; H-3', H-4', H-5'), 3.72-3.70 (m, 1H; H$_b$-6'), 3.56 (s, 3H, OCH$_3$), 3.36 (dd, J = 10.3, 3.7 Hz, 1H; H-2'); ^{13}C NMR (150 MHz, CDCl$_3$): δ 168.1 (C), 167.2 (C), 137.1 (C), 136.96 (C), 136.95 (C), 136.8 (C), 131.5 (CH), 129.1 (CH), 128.67 (CH), 128.66 (CH), 128.5 (CH), 128.35 (CH), 128.33 (CH), 128.2 (CH), 128.14 (CH), 128.10 (CH), 121.6 (C), 99.8 (CH), 98.3 (CH), 98.1 (CH), 80.6 (CH), 79.5 (CH), 79.0 (CH), 77.85 (CH), 77.83 (CH), 73.8 (CH$_2$), 72.58 (CH$_2$), 72.57 (CH), 72.4 (CH$_2$), 72.2 (CH), 71.9 (CH), 71.6 (CH$_2$), 69.5 (CH), 68.5 (CH), 63.2 (CH), 61.1 (CH$_2$), 57.3 (CH$_3$); HRMS m/z (ESI, M+Na$^+$) calcd for $C_{47}H_{48}BrN_3O_{15}Na^+$ 996.2161, found 996.2162.

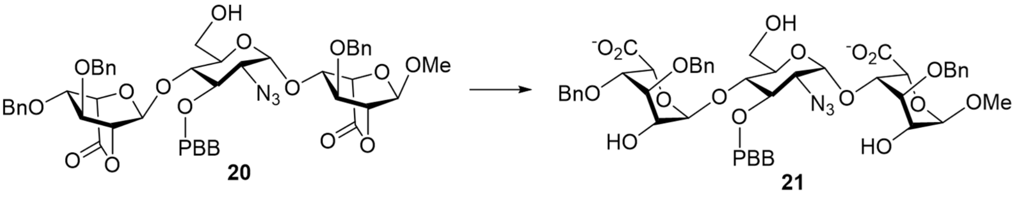

Compound 21. To a solution of compound **20** (22.2 mg, 0.02 mmol) in THF (1 mL) was added LiOH (1.0 M solution in water, 0.14 mL) at room temperature. After 1 h, the reaction was quenched by sat. $NH_4Cl_{(aq)}$, and the aqueous phase was extracted by ethyl acetate (3 x 3 mL). The combined organic layers were washed with brine, dried over anhydrous $MgSO_4$, filtered, and concentrated *in vacuo*. The residue was purified by flash column chromatography (chloroform/methanol = 10/1) on silica gel to obtain compound **21** (20.8 mg, 90%) as a white solid. IR (thin film in KBr) ν 3411, 2919, 2113, 1606, 1411, 1100, 1028, 803, 737, 698 cm^{-1}; ^1H NMR (600 MHz, CD_3OD): δ 7.38-7.35 (m, 6H; Ar-H), 7.33-7.29 (m, 4H; Ar-H), 7.28-7.26 (m, 2H; Ar-H), 7.25-7.22 (m, 7H; Ar-H), 5.24 (d, *J* = 2.1 Hz, 1H; H-1”), 4.99 (d, *J* = 3.6 Hz, 1H; H-1’), 4.91 (d, *J* = 11.4, 1H; Ar-CH_2), 4.80 (s, 1H; H-1), 4.70-4.63 (m, 4H; H-5”, Ar-CH_2), 4.59-4.57 (m ,2H; Ar-CH_2), 4.53-4.50 (m, 3H; H-5, Ar-CH_2), 4.27 (s, 1H; H-4), 4.10-4.08 (m, 1H; H-4’), 4.02-3.99 (m,2H; H-3’, H-5’), 3.89-3.80 (m, 3H; H-3, H-3”, H_a-6’), 3.75-3.73 (m, 2H; H-4”, H_b-6’), 3.68-3.66 (m, 2H; H-2, H-2”), 3.55 (dd, *J* = 9.9, 3.6 Hz, 1H; H-2’), 3.40 (s, 3H; OCH_3); ^{13}C NMR (150 MHz, CD_3OD): δ 176.5 (C), 175.9 (C), 139,6 (C), 139.4 (C), 139.3 (C), 138.9 (C), 132.3 (CH), 130.9 (CH), 129.6 (CH), 129.4 (CH), 129.3 (CH), 128.94 (CH), 128.92 (CH), 128.8 (CH), 128.7 (CH), 128.6 (CH), 122.2 (C), 103.8 (CH), 100.1 (CH), 95.8 (CH), 79.5 (CH), 77.9 (CH), 77.6 (CH), 76.3 (CH), 75.1 (CH_2), 74.3 (CH), 74.2 (CH_2), 73.4 (CH), 72.7 (CH_2), 72.5 (CH), 71.8 (CH), 70.5 (CH), 69.1 (CH), 68.2 (CH), 65.6 (CH), 62.0 (CH_2), 55.9 (CH_3); HRMS m/z (ESI, $M+H^+$) calcd for $C_{47}H_{53}BrN_3O_{17}^+$ 1010.2553, found 1010.2595.

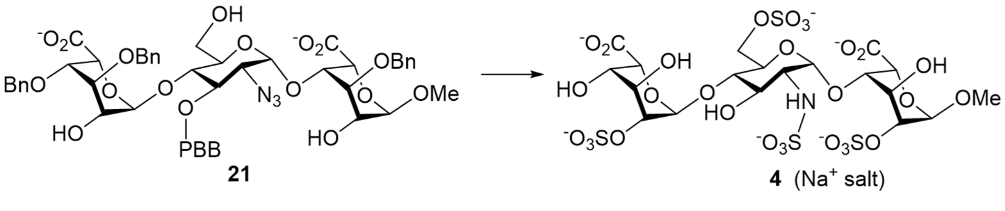

Compound 4. Compound **21** was subjected to three consecutive reactions: (1) the general

procedure for *O*-sulfonation (Method A) to furnish the *O*-sulfonated derivative **22** (77%); (2) the general procedure for hydrogenolysis (Method B) to yield the deprotected compound **23** (90%); and the general procedure for *N*-sulfonation (Method C) to provide the trisaccharide **4** (77%). ^{1}H NMR (600 MHz, D_2O): δ 5.32 (s, 1H; H-1'), 5.16 (s, 1H; H-1"), 5.03 (s, 1H; H-1), 4.84 (s, 1H; H-5"), 4.44 (s, 1H; H-5), 4.31-4.30 (m, 2H; H-2", H_a-6'), 4.26-4.21 (m, 3H; H-2, H-4", H_b-6'), 4.09 (s, 1H; H-3"), 4.04-4.01 (m, 2H; H-3, H-4'), 3.97 (s, 1H; H-4), 3.77-3.69 (m, 2H; H-3', H-5'), 3.40 (s, 3H; OCH_3), 3.25 (m, 2H; H-2'); ^{13}C NMR (150 MHz, D_2O): δ 176.4 (C), 175.2 (C), 99.6 (CH), 99.0 (CH), 96.9 (CH), 76.5 (CH), 75.7(CH), 75.2 (CH), 73.9 (CH), 69.5 (CH), 69.1 (CH), 68.8 (CH), 68.7 (CH), 68.0 (CH), 67.8 (CH), 66.3 (CH_2), 58.0 (CH), 55.3 (CH_3); HRMS m/z (ESI, M+6Na$^+$+H$^+$) calcd for $C_{19}H_{26}NO_{29}S_4Na_6^+$ 997.8854, found 997.8815.

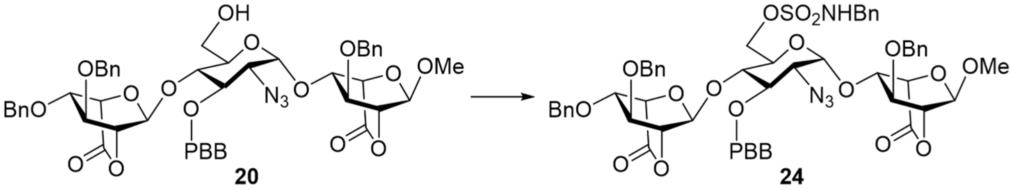

Compound 24. *N*-Benzylsulfamoyl chloride (0.30 mL, 2.05 mmol) was added to a solution of compound **20** (0.20 g, 0.21 mmol) in pyridine (20 mL) at room temperature under nitrogen atmosphere. After 10 mins, the reaction was quenched by methanol. The solvent was concentrated *in vacuo*, and the residue was purified by flash column chromatography (hexane/ethyl acetate = 3/1) on silica gel to yield the compound **24** (0.18 g, 78%) as a white solid. IR (thin film in KBr) ν 3315, 3031, 2927, 2110, 1787, 1489, 1455, 1365, 1319, 1261, 1208, 1173, 1143, 1081, 1036, 931, 870, 806, 750, 698, 615, 540 cm^{-1}; ^{1}H NMR (600 MHz, $CDCl_3$): δ 7.46 (d, *J* = 8.3 Hz, 2H; Ar-H), 7.42-7.41 (m, 2H; Ar-H), 7.39-7.36 (m, 2H; Ar-H), 7.35-7.33 (m, 4H; Ar-H), 7.31-7.28 (m, 10H; Ar-H), 7.24 (s, 2H; Ar-H), 7.20 (d, *J* = 8.2 Hz, 2H; Ar-H), 5.30 (s, 1H; H-1"), 5.07 (s, 1H; H-1), 5.02 (d, *J* = 3.6 Hz, 1H; H-1'), 4.86 (d, *J* = 10.6 Hz, 1H; Ar-CH_2), 4.75 (ABq, *J* = 12.1 Hz, 2H; Ar-CH_2), 4.69-4.67 (m, 2H; Ar-CH_2), 4.63-4.59 (m, 4H; H-2, H-2", NH, Ar-CH_2), 4.53 (d, *J* = 11.7 Hz, 1H; Ar-CH_2), 4.44 (d, *J* = 3.2 Hz, 1H; H-5), 4.40 (d, *J* = 11.7 Hz, 1H; Ar-CH_2), 4.34-4.32 (m, 1H; H_a-6'), 4.27-4.25 (m, 2H; H-4, H-5"), 4.22-4.17 (m, 2H; Ar-CH_2), 4.06-4.04 (m, 1H; H_b-6'), 4.00 (t, *J* = 3.9 Hz; H-4"), 3.88-3.87 (m, 1H; H-3), 3.85-3.82 (m, 2H; H-3", H-5'), 3.79-3.76 (m, 1H; H-3'), 3.65-3.62 (m, 1H; H-4'), 3.55 (s, 1H; OCH_3), 3.28 (dd, *J* = 10.2, 3.7 Hz; H-2'); ^{13}C NMR (150 MHz, $CDCl_3$):

δ 167.9 (C), 167.3 (C), 137.1 (C), 136.92 (C), 136.91 (C), 136.7 (C), 136.0 (C), 131.6 (CH), 129.4 (CH), 129.0 (CH), 128.9 (CH), 128.7 (CH), 128.6 (CH), 128.4 (CH), 128.3 (CH), 128.2 (CH), 128.15 (CH), 128.12 (CH), 127.9 (CH), 121.9 (C), 99.7 (CH), 98.6 (CH), 98.1 (CH), 80.8 (CH), 79.8 (CH), 78.9 (CH), 78.2 (CH), 77.9 (CH), 74.0 (CH_2), 72.7 (CH_2), 72.6 (CH), 72.4 (CH_2), 72.1 (CH), 71.8 (CH_2), 69.5 (CH), 69.3 (CH), 68.7 (CH), 67.8 (CH_2), 63.3 (CH), 57.3 (CH_3), 47.9 (CH_2); HRMS m/z (ESI, $M+H^+$) calcd for $C_{54}H_{56}BrN_4O_{17}S^+$ 1201.2570, found 1201.2559.

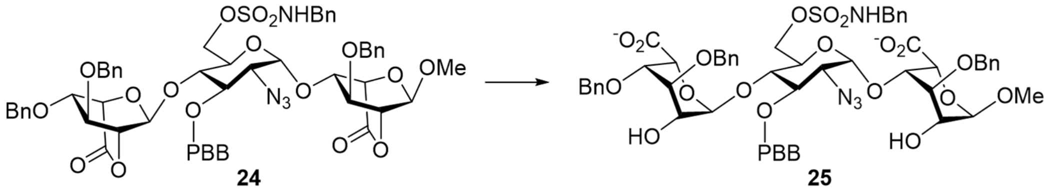

Compound 25. To a solution of compound **24** (0.15 g, 0.13 mmol) in THF (1.5 mL) was added a 1.0 M LiOH in water (0.8 mL) at room temperature. After stirring for 1 h, the reaction was quenched by sat. $NH_4Cl_{(aq)}$, and the aqueous phase was extracted by ethyl acetate (3 x 3 mL). The combined organic layers were washed with brine, dried over anhydrous $MgSO_4$, filtered, and concentrated *in vacuo*. The residue was purified by flash column chromatography (chloroform/methanol = 10/1) on silica gel to generate compound **25** (0.13 g, 90%) as a white solid. IR (thin film in KBr) ν 3431, 3032, 2939, 2116, 1733, 1607, 1489, 1455, 1362, 1257, 1173, 1148, 1099, 1071, 1030, 1012, 914, 868, 805, 750, 699, 613, 542 cm^{-1}; ^1H NMR (600 MHz, CD_3OD): δ 7.40-7.35 (m, 6H; Ar-H), 7.33-7.29 (m, 5H; Ar-H), 7.28-7.23 (m, 12H; Ar-H), 7.21-7.18 (m, 1H; Ar-H), 5.28 (d, *J* = 3.7 Hz, 1H; H-1''), 4.98 (d, *J* = 3.7 Hz, 1H; H-1'), 4.92 (d, *J* = 11.3 Hz, 1H; Ar-CH_2), 4.80 (s, 1H; H-1), 4.69 (d, *J* = 11.8 Hz, 1H; Ar-CH_2), 4.65-4.62 (m, 5H; H-5'', Ar-CH_2), 4.59-4.53 (m, 3H; H-5, Ar-CH_2), 4.50 (d, *J* = 10.5 Hz, 1H; H_a-6'), 4.37 (d, *J* = 10.1 Hz, 1H; H_b-6'), 4.27-4.23 (m, 4H; H-4, H-4', Ar-CH_2), 4.00-3.97 (m, 1H; H-3'), 3.94-3.91 (m, 2H; H-4'', H-5'), 3.81-3.79 (m, 2H; H-3, H-3''), 3.68-3.66 (m, 2H; H-2, H-2''), 3.55 (dd, *J* = 6.8, 3.6 Hz, 1H; H-2'), 3.41 (s, 3H; OCH_3); ^{13}C NMR (150 MHz, CD_3OD): δ 176.3 (C), 175.1 (C), 139.7 (C), 139.4 (C), 139.2 (C), 139.0 (C), 138.9 (C), 132.3 (CH), 130.8 (CH), 129.5 (CH), 129.4 (CH), 129.37 (CH), 129.34 (CH), 129.32 (CH), 129.1 (CH), 128.99 (CH), 128.95 (CH), 128.8 (CH), 128.7 (CH), 128.6 (CH), 128.5 (CH), 122.2 (C), 103.8 (CH), 100.9 (CH), 95.9 (CH), 79.7 (CH), 78.2 (CH), 76.2 (CH), 75.0 (CH), 74.6 (CH), 74.2 (CH_2),

73.9 (CH_2), 73.7 (CH), 72.9 (CH_2), 72.3 (CH), 71.2 (CH), 70.1 (CH), 69.1 (CH), 68.9 (CH_2), 68.4 (CH), 65.1 (CH), 56.0 (CH_3), 48.1 (CH_2); HRMS m/z (ESI, M+Na^+) calcd for $C_{54}H_{59}BrN_4O_{19}SNa^+$ 1143.2539, found 1143.2558.

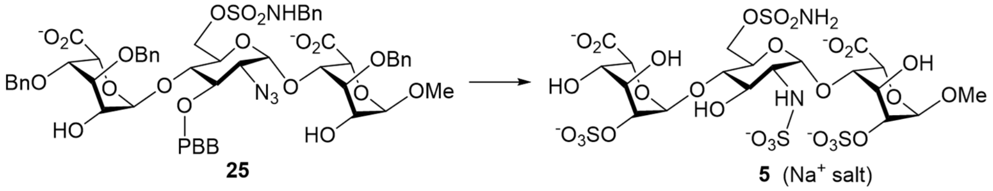

Compound 5. Compound **25** was subjected to three consecutive reactions: (1) the general procedure for *O*-sulfonation (Method A) to furnish the *O*-sulfonated derivative **26** (70%); (2) the general procedure for hydrogenolysis (Method B) to yield the deprotected compound **27** (73%); and the general procedure for *N*-sulfonation (Method C) to provide the trisaccharide **5** (75%). ^{1}H NMR (600 MHz, D_2O) δ = 5.33 (d, *J* = 3.4, 1H, H-1'), 5.14 (s, 1H, H-1"), 5.03 (s, 1H, H-1), 4.89-4.87 (m, 1H, H-5"), 4.48-4.43 (m, 3H, H-5, H_a-6', H_b-6'), 4.30-4.21 (m, 3H, H-2, H-2", H-3), 4.10-3.97 (m, 4H, H-3", H-4, H-4", H-5'), 3.75-3.70 (m, 2H, H-3', H-4'), 3.40 (s, 3H, OCH_3), 3.25 (dd, 1H, *J* = 3.3, 12.2 Hz, H-2'); ^{13}C NMR (150 MHz, D_2O) δ =176.3 (C), 175.0 (C), 99.5 (CH), 99.4 (CH), 97.5 (CH), 77.6 (CH), 76.1 (CH), 74.5 (CH), 73.9 (CH), 69.4 (CH), 68.9 (CH), 68.8 (CH), 68.7 (CH), 68.6 (CH), 68.3 (CH_2), 67.5 (CH), 67.3 (CH), 58.1 (CH), 55.4 (OCH_3); HRMS m/z (ESI, M-6H^++5Na^+) calcd for $C_{19}H_{26}N_2O_{28}S_4Na_5^-$ 972.9049, found 972.9021.

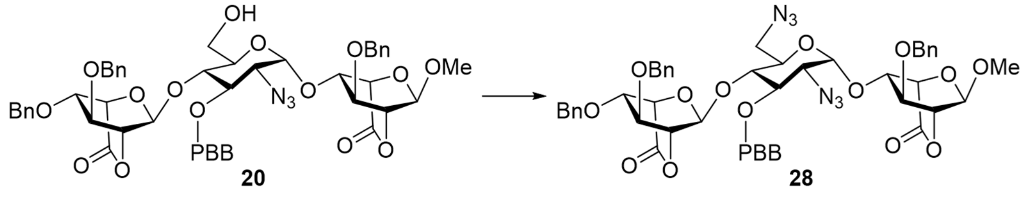

Compound 28. Compound **20** (0.11 g, 0.11 mmol) was dissolved in toluene (1.1 mL) at room temperature under atmosphere. DPPA (28 μL, 0.13 mmol) and DBU (20 μL, 0.13 mmol) were sequentially added to the solution, and the resulting mixture was warmed up to 100 °C. After stirring for 24 h, the reaction flask was cooled down to room temperature, and the mixture was diluted with ethyl acetate followed by washed with water and brine. The organic layer was

dried over anhydrous $MgSO_4$, filtered, and concentrated *in vacuo*. The residue was purified by flash column chromatography (hexane/ethyl acetate = 4/1) on silica gel, and compound **28** (65 mg) was obtained in 60% yield as a white solid. $[\alpha]^{25.5}_{D}$ = -8.40 (c = 1.0, $CHCl_3$); IR (thin film in KBr) ν 3031, 2926, 2107, 1789, 1593, 1489, 1455, 1367, 1316, 1207, 1156, 1080, 1036, 911, 871, 807, 751, 698 cm^{-1}; ^1H NMR (600 MHz, $CDCl_3$): δ 7.46 (d, *J* = 8.3 Hz, 2H; Ar-H), 7.41 (d, *J* = 6.8 Hz, 2 H; Ar-H), 7.39-7.29 (m, 12H; Ar-H), 7.27-7.19 (m, 3H; Ar-H), 5.32 (s, 1H; H-1"), 5.07-5.06 (m, 2H; H-1, H-1'), 4.88 (d, *J* = 10.8 Hz, 1H; Ar-CH_2), 4.75 (ABq, *J* = 12.1 Hz, 2H; Ar-CH_2), 4.65 (d, *J* = 11.0 Hz, 1H; Ar-CH_2), 4.62-4.58 (m, 2H; H-2, Ar-CH_2), 4.54-4.46 (m, 4H; H-2", Ar-CH_2), 4.34-4.26 (m, 3H; H-4", H-5, H-5"), 3.90-3.89 (m, 1H; H-4), 3.87-3.85 (m, 2H; H-3, H-3"), 3.83-3.82 (m, 1H; H-4'), 3.77-3.74 (m, 2H; H-3', H_a-6'), 3.55 (s, 3H; OCH_3), 3.54-3.50 (m, 1H; H_b-6'), 3.38-3.35 (m, 2H; H-2', H-5'); ^{13}C NMR (150 MHz, $CDCl_3$): δ 167.8 (C), 167.1 (C), 136.9 (C), 136.8 (C), 136.7 (C), 131.6 (CH), 129.5 (CH), 129.3 (CH), 128.7 (CH), 128.6 (CH), 128.5 (CH), 128.3 (CH), 128.25 (CH), 128.22 (CH), 128.1 (CH), 127.9 (CH), 127.8 (CH), 121.8 (C), 99.6 (CH), 98.4 (CH), 98.1 (CH), 80.6 (CH), 79.6 (CH), 78.8 (CH), 78.1 (CH), 77.8 (CH), 77.7 (CH), 73.9 (CH_2), 72.8 (CH_2), 72.6 (CH), 72.4 (CH), 72.3 (CH_2), 71.7 (CH_2), 70.7 (CH), 69.5 (CH), 68.5 (CH), 63.3 (CH), 57.3 (CH_3), 50.4 (CH_2); HRMS m/z (ESI, M+Na^+) calcd for $C_{47}H_{47}BrN_6O_{14}Na^+$ 1021.2226, found 1021.2230.

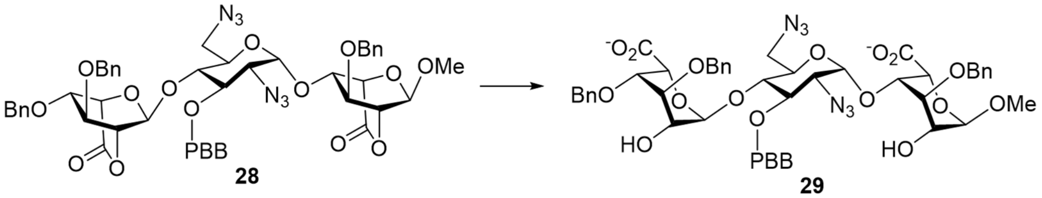

Compound 29. LiOH (1.0 M solution in water, 0.39 mL) was added to a solution of compound **28** (65 mg, 0.65mmol) in THF (1 mL) at room temperature. After stirring for 1 h, the reaction was quenched with sat. $NH_4Cl_{(aq)}$, and the aqueous layer was extracted by ethyl acetate (3 x 3 mL). The combined organic layers were washed with brine, dried over anhydrous $MgSO_4$, filtered, and concentrated *in vacuo*. The residue was purified by flash column chromatography (chloroform/methanol = 10/1) on silica gel to give compound **29** (61 mg, 86%) as a white solid. IR (thin film in KBr) ν 3410, 2911, 2110, 1606, 1410, 1099, 1031, 805, 738, 699 cm^{-1}; ^1H NMR (600 MHz, CD_3OD): δ 7.39-7.38 (m, 4H; Ar-H), 7.36-7.2 (m, 3H; Ar-H), 7.31-7.29 (m, 2H; Ar-H), 7.27-7.23 (m, 10 H; Ar-H), 5.19 (d, *J* = 3.4 Hz, 1H; H-1"), 4.99 (d, *J* = 3.7 Hz, 1H; H-

1'), 4.91 (d, J = 11.3 Hz, 1H; Ar-CH_2), 4.79 (s, 1H; H-1), 4.71 (d, J = 11.9 Hz, 1H; Ar-CH_2), 4.68-4.65 (m, 2H; Ar-CH_2), 4.61-4.56 (m, 5H; H-5'', Ar-CH_2), 4.54-4.53 (s, 1H; H-5), 4.28-4.24 (m, 2H; H-4, H-4'), 4.01-3.98 (m 2H; H-3', H-4''), 3.91-3.88 (m, 1H; H-5'), 3.80-3.79 (m, 1H; H-3), 3.76-3.74 (m, 1H; H-3''), 3.65-3.62 (m, 3H; H-2, H-2'', H_a-6'), 3.60-3.58 (dd, J = 10.0, 3.7 Hz, 1H; H-2'), 3.55-3.52 (m, 1H; H_b-6'), 3.41 (s, 3H; OCH_3); ^{13}C NMR (150 MHz, CD_3OD): δ 176.4 (C), 175.8 (C), 139.7 (C), 139.4 (C), 139.3 (C), 138.9 (C), 132.3 (CH), 131.0 (CH), 129.5 (CH), 129.4 (CH), 129.3 (CH), 129.2 (CH), 129.0 (CH), 128.9 (CH), 128.8 (CH), 128.6 (CH), 122.3 (C), 103.8 (CH), 100.6 (CH), 95.5 (CH), 79.4 (CH), 78.4 (CH), 78.2 (CH), 77.3 (CH), 75.1 (CH_2), 74.3 (CH_2), 74.1 (CH), 73.5 (CH_2), 73.2 (CH), 72.8 (CH_2), 72.4 (CH), 71.6 (CH), 71.1 (CH), 69.1 (CH), 68.4 (CH), 65.4 (CH), 55.9 (CH_3), 52.1 (CH_2); HRMS m/z (ESI, $M+H^++2Na^+$) calcd for $C_{47}H_{50}BrN_6O_{16}Na_2^+$ 1079.2257, found 1079.2257.

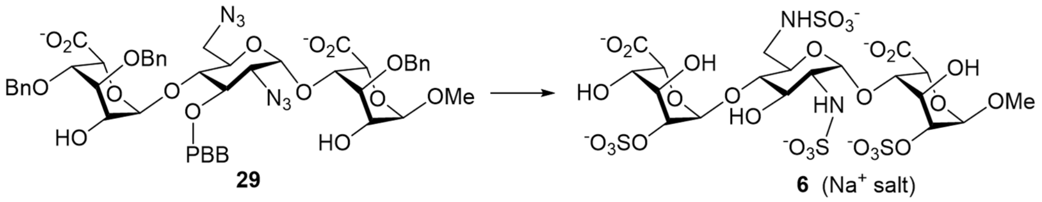

29 **6** (Na$^+$ salt)

Compound 6. Compound **29** was subjected to three consecutive reactions: (1) the general procedure for *O*-sulfonation (Method A) to furnish the *O*-sulfonated derivative **30** (86%); (2) the general procedure for hydrogenolysis (Method B) to yield the deprotected compound **31** (40%); and the general procedure for *N*-sulfonation (Method C) to provide the trisaccharide **6** (78%). ^1H NMR (600 MHz, D_2O): δ 5.31 (d, J = 3.5 Hz, 1H; H-1'), 5.24 (s, 1H; H-1''), 5.12 (s, 1H; H-1), 4.86 (d, J = 2.1 Hz, 1H; H-5''), 4.51 (d, J = 2.2 Hz, 1H; H-5), 4.36-4.35 (m, 1H; H-2''), 4.28-4.26 (m, 2H; H-2, H-3), 4.15 (t, J = 3.1 Hz,1H; H-3''), 4.08-4.07 (m, 1H; H-4), 4.03 (s, 1H; H-4''), 4.00-3.97 (m, 1H; H-5'), 3.79-3.74 (m, 1H; H-3'), 3.71-3.68 (m, 1H;, H-4'), 3.45 (s, 3H; OCH_3), 3.38-3.36 (m, 1H; H_a-6'), 3.29 (dd, J = 10.4, 3.5 Hz 1H; H-2'), 3.27-3.24 (m, 1H; H_b-6'); ^{13}C NMR (150 MHz, D_2O): δ 176.5 (C), 175.2 (C), 99.44(CH), 99.42 (CH), 96.9 (CH), 79.1 (CH), 75.4 (CH), 74.4 (CH), 73.7 (CH), 69.4 (CH), 69.3 (CH), 68.8 (CH), 68.7 (CH), 68.6 (CH), 67.4 (CH), 67.3 (CH), 58.1 (CH), 55.5 (CH_3), 43.3 (CH_2); HRMS m/z (ESI, $M+Na^+$) calcd for $C_{19}H_{28}N_2O_{28}S_4Na_5^+$ 974.9194, found 974.9195.

5. References

1. Lamanna, W. C.; Frese, M.-A.; Balleininger, M.; Dierks, T., Sulf Loss Influences *N*-, 2-*O*-, and 6-*O*-Sulfation of Multiple Heparan Sulfate Proteoglycans and Modulates Fibroblast Growth Factor Signaling. *J. Biol. Chem.* **2008**, *283*, 27724-27735.
2. Chiu, L.-T.; Sabbavarapu, N. M.; Lin, W.-C.; Fan, C.-Y.; Wu, C.-C.; Cheng, T.-J. R.; Wong, C.-H.; Hung, S.-C., Trisaccharide Sulfate and Its Sulfonamide as an Effective Substrate and Inhibitor of Human Endo-*O*-sulfatase-1. *J. Am. Chem. Soc.* **2020**, *142*, 5282-5292.
3. Hu, Y.-P.; Lin, S.-Y.; Huang, C.-Y.; Zulueta, M. M. L.; Liu, J.-Y.; Chang, W.; Hung, S.-C., Synthesis of 3-*O*-Sulfonated Heparan Sulfate Octasaccharides that Inhibit the Herpes Simplex Virus Type 1 Host–Cell Interaction. *Nat. Chem.* **2011**, *3*, 557-563.

6. NMR and HRMS data

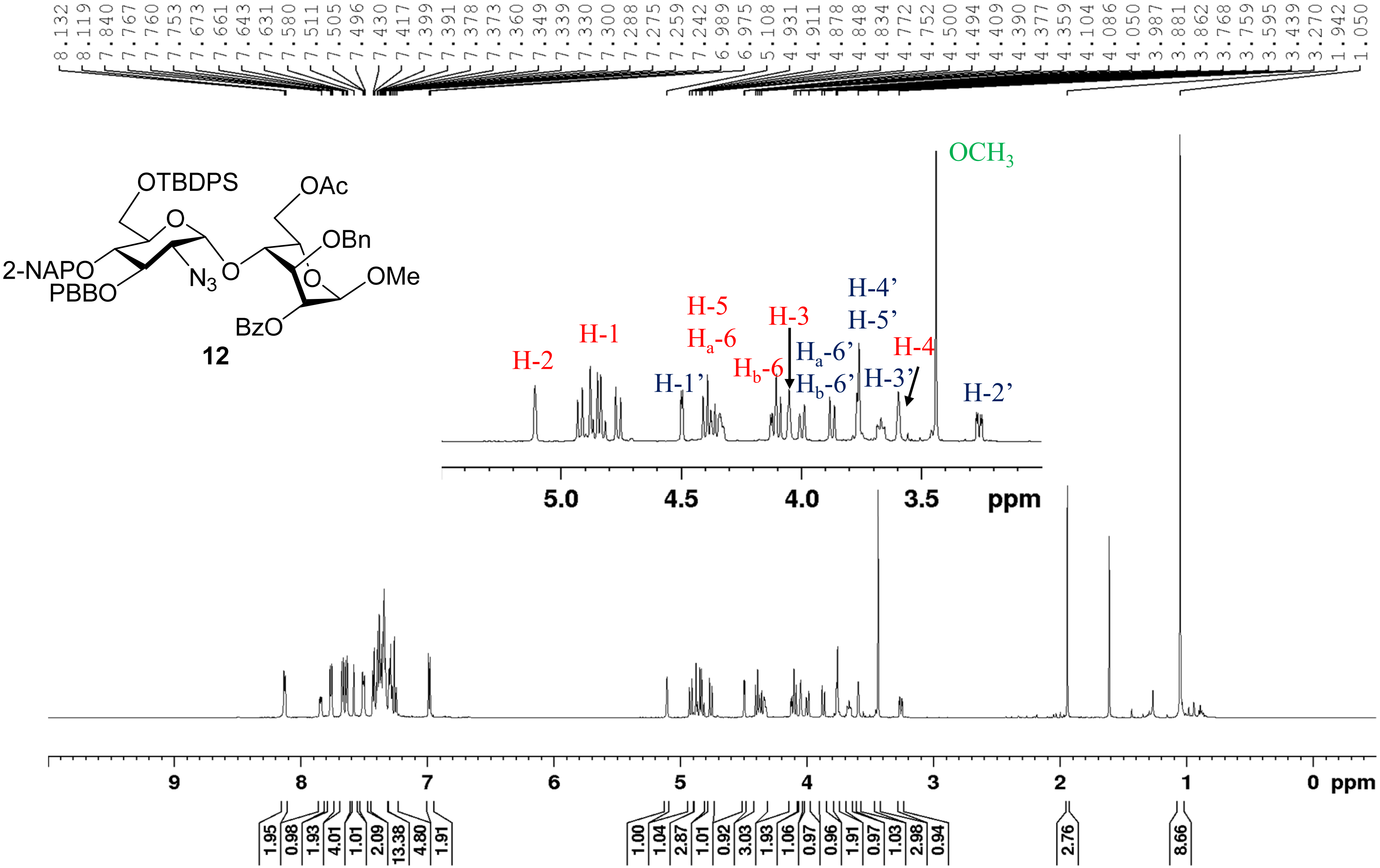

12

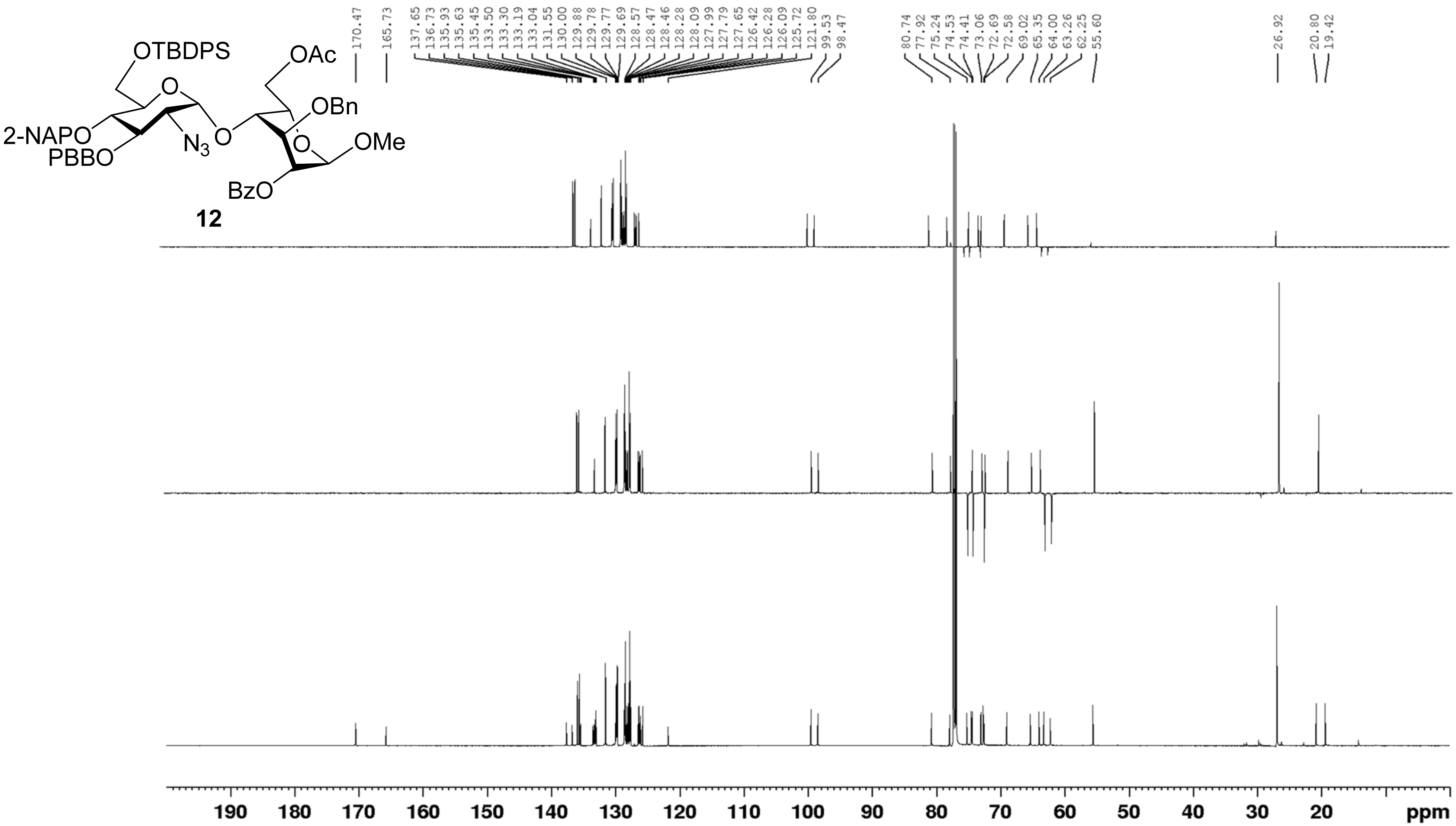

12

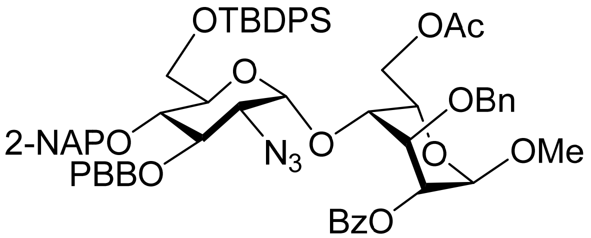
OTBDPS
OAc
OBn
2-NAPO
PBBO
N3
O
OMe
BzO

12

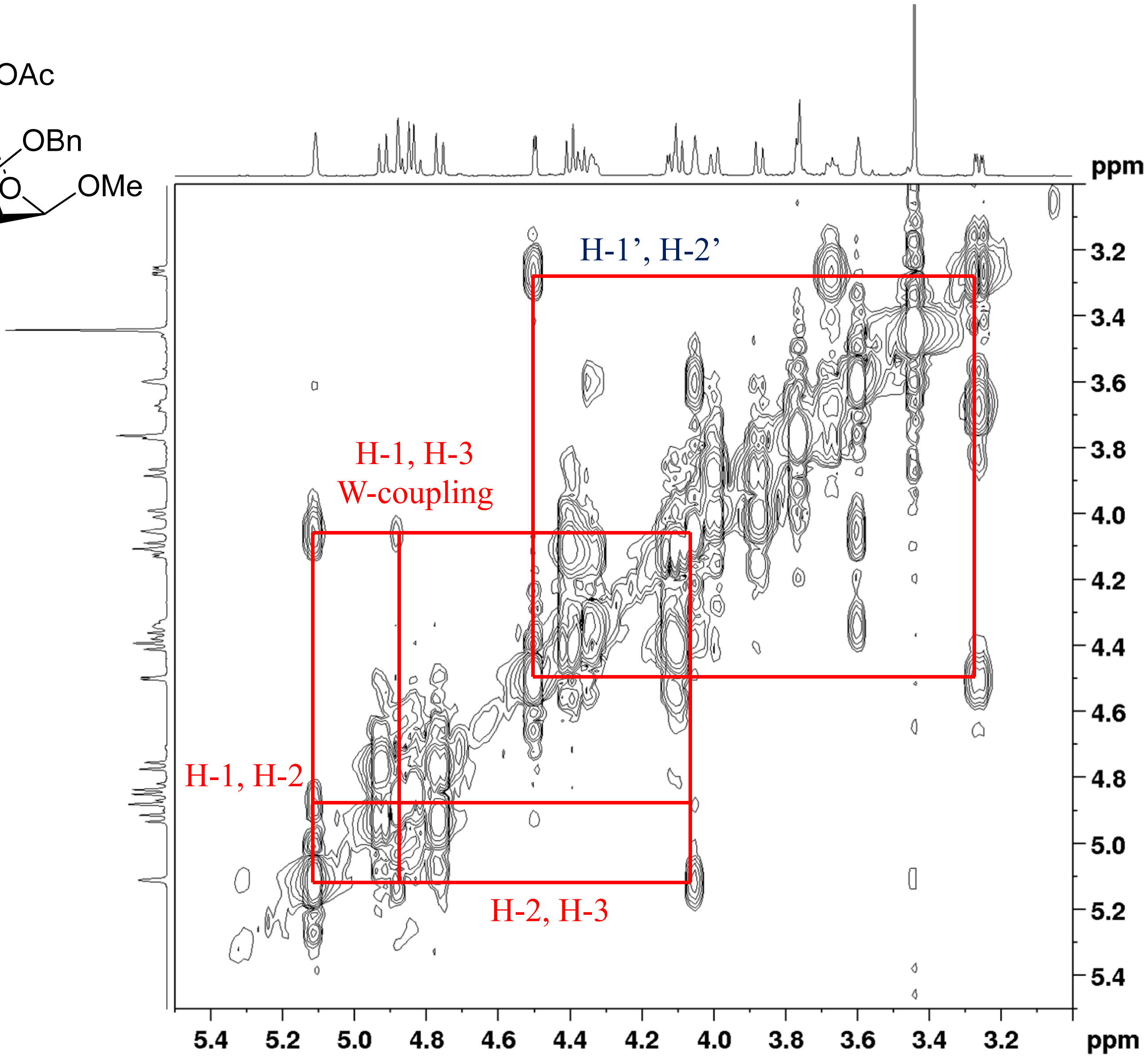
H-1’, H-2’
H-1, H-3
W-coupling
H-1, H-2
H-2, H-3
ppm
3.2
3.4
3.6
3.8
4.0
4.2
4.4
4.6
4.8
5.0
5.2
5.4
ppm
5.4
5.2
5.0
4.8
4.6
4.4
4.2
4.0
3.8
3.6
3.4
3.2
ppm

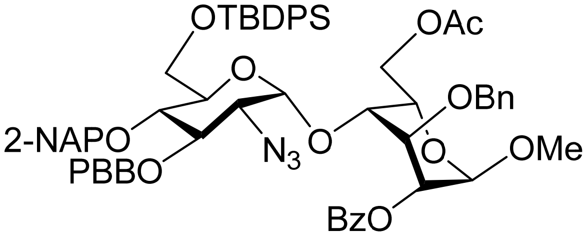
OTBDPS
OAc
OBn
2-NAPO
PBBO
N3
O
OMe
BzO

12

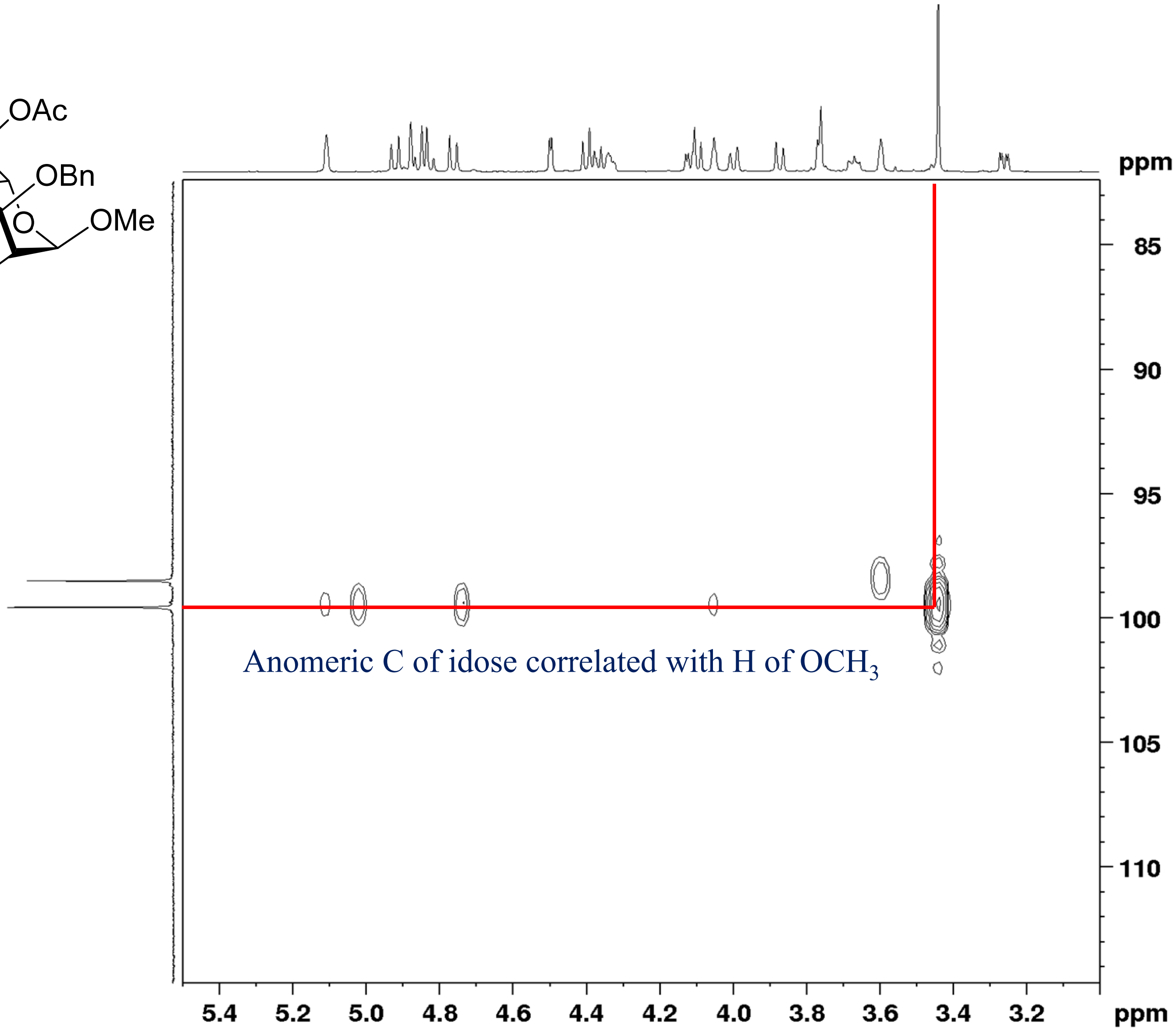
ppm
85
90
95
100
105
110
Anomeric C of idose correlated with H of OCH3
5.4
5.2
5.0
4.8
4.6
4.4
4.2
4.0
3.8
3.6
3.4
3.2
ppm

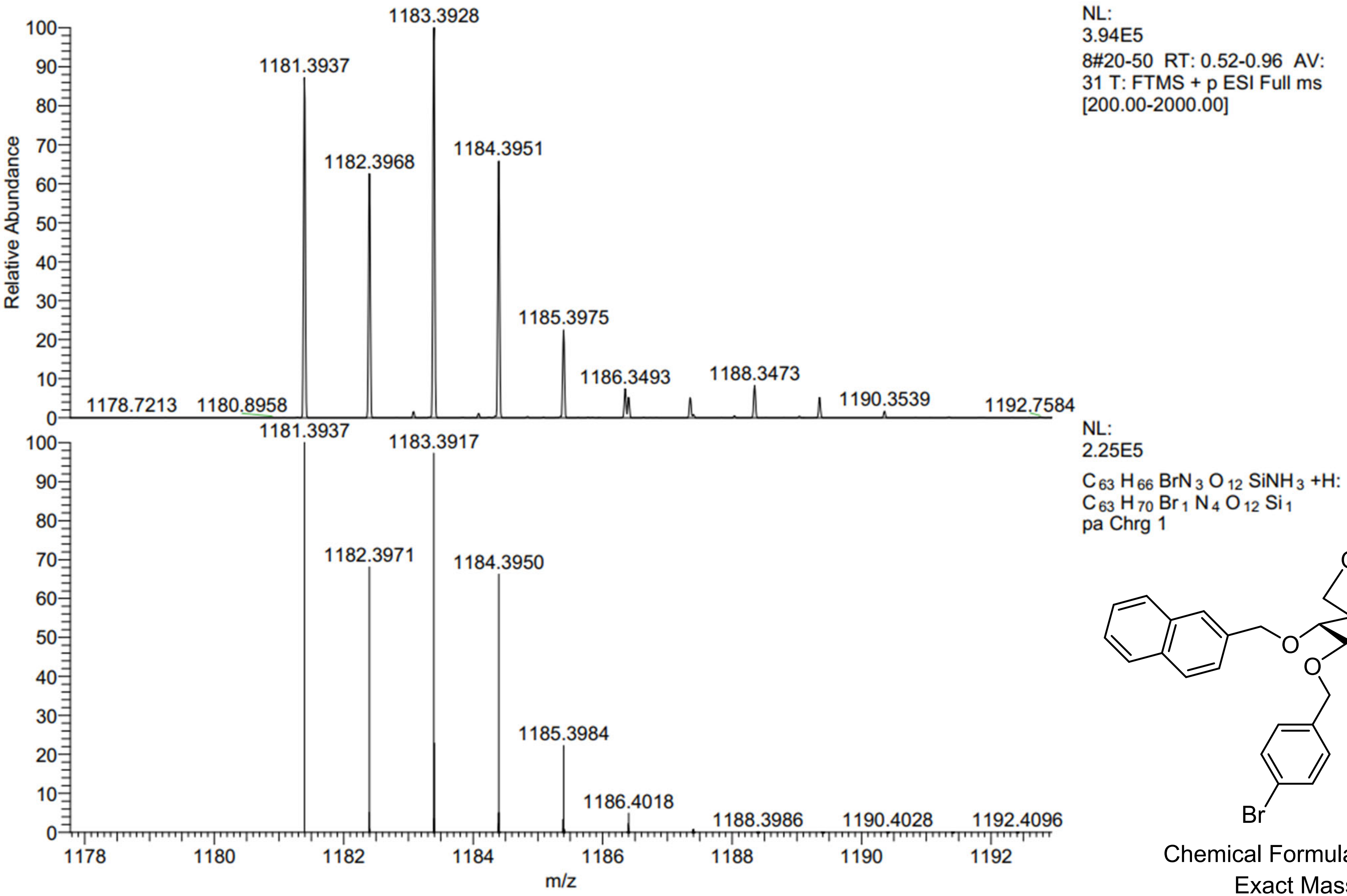

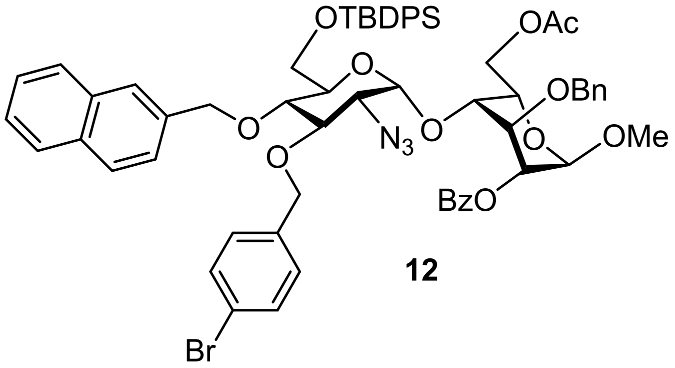

12

Chemical Formula: $C_{63}H_{66}BrN_3O_{12}Si$

Exact Mass: 1163.3599

Molecular Weight: 1165.2190

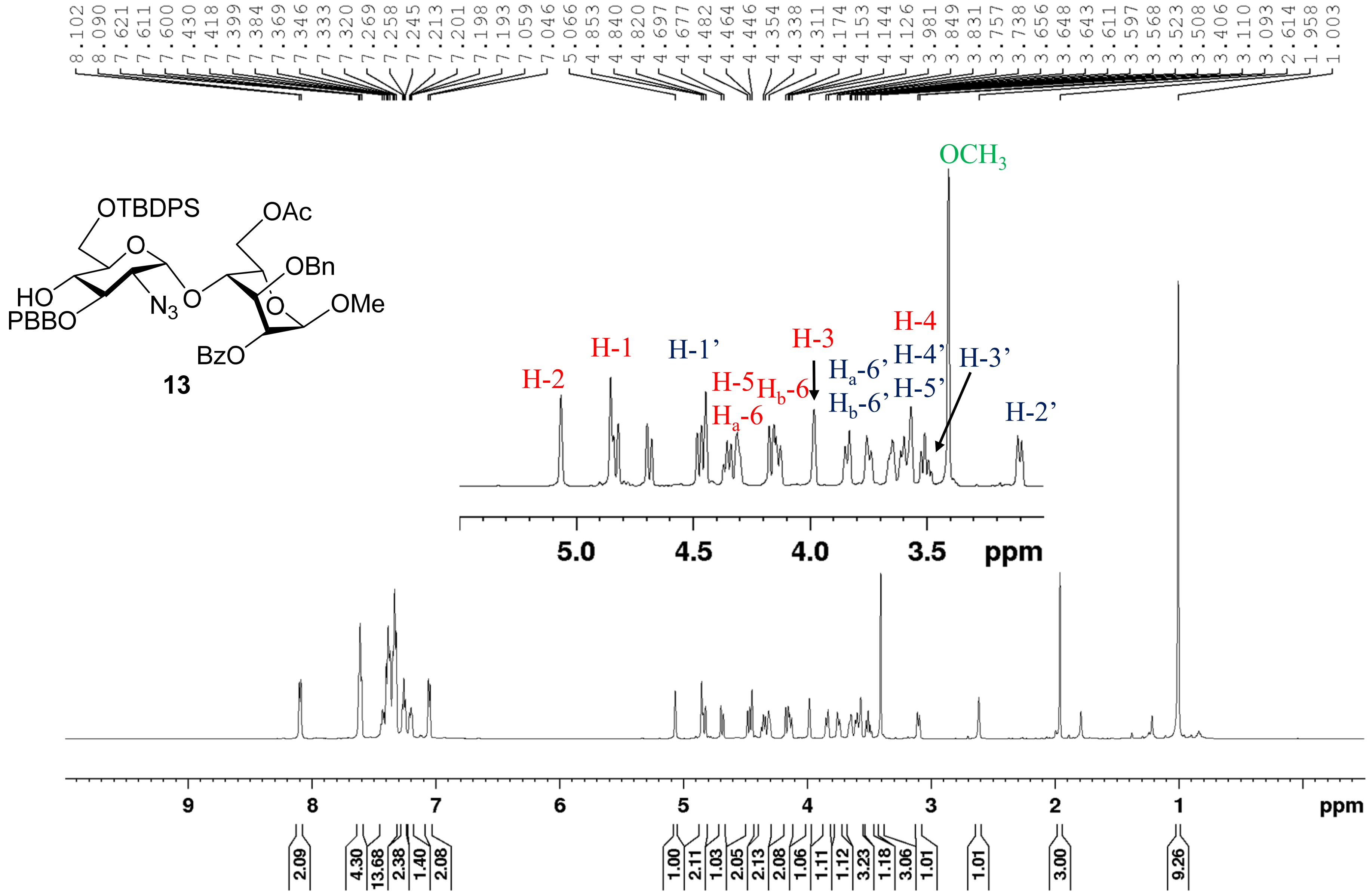

OTBDPS
OAc
OBn
HO
PBBO
N3
O
OMe
BzO
13
OCH3
H-2
H-1
H-1'
H-5
Ha-6
Hb-6
H-3
Ha-6', Hb-6'
H-4
H-4'
H-5'
H-3'
H-2'
ppm

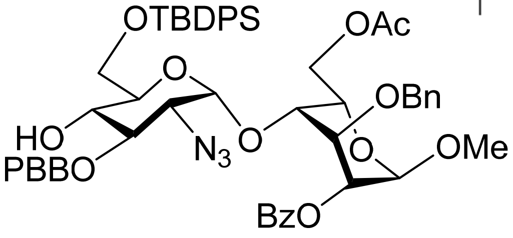
OTBDPS
OAc
OBn
HO
PBBO
N3
OMe
BzO

13

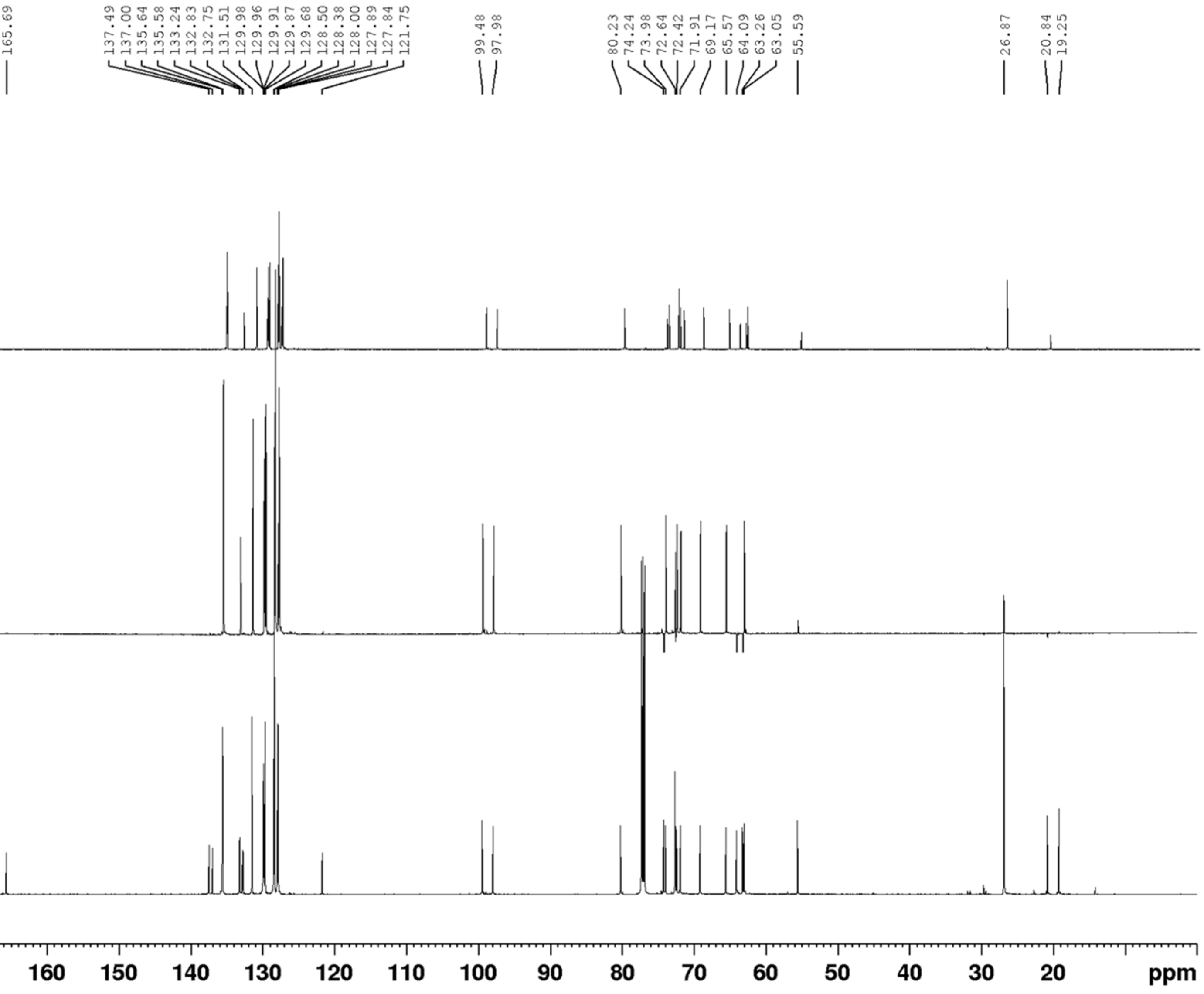
170.52
165.69
137.49
137.00
135.64
135.58
133.24
132.83
132.75
131.51
129.98
129.96
129.91
129.87
129.68
128.50
128.38
128.00
127.89
127.84
121.75
99.48
97.98
80.23
74.24
73.98
72.64
72.42
71.91
69.17
65.57
64.09
63.26
63.05
55.59
26.87
20.84
19.25
190 180 170 160 150 140 130 120 110 100 90 80 70 60 50 40 30 20 ppm

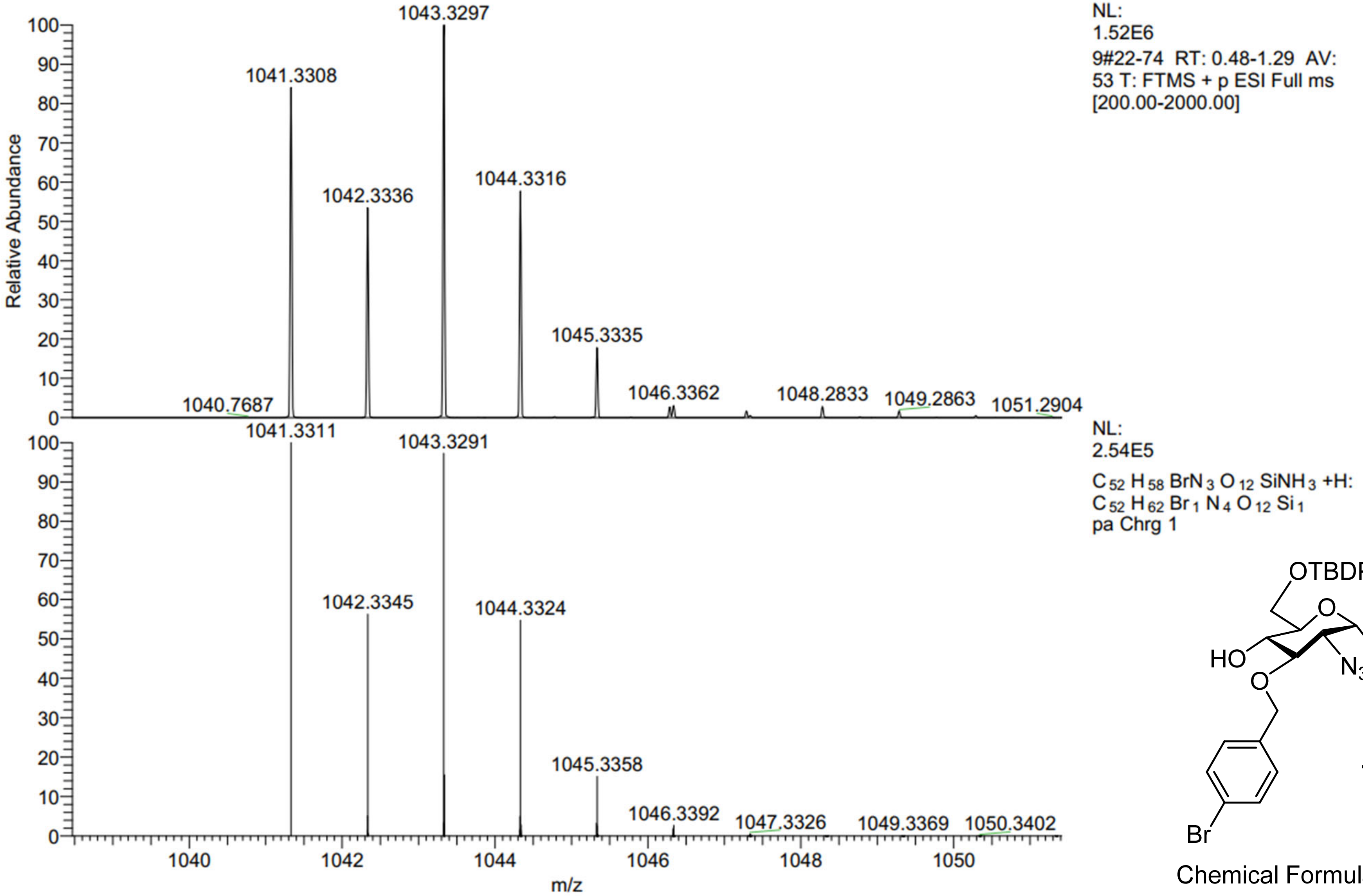

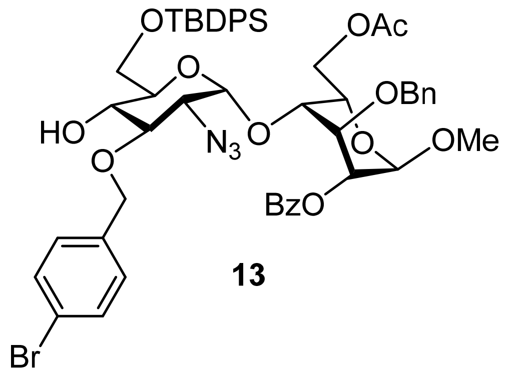

13

Chemical Formula: $C_{52}H_{58}BrN_3O_{12}Si$
Exact Mass: 1023.2973
Molecular Weight: 1025.0340

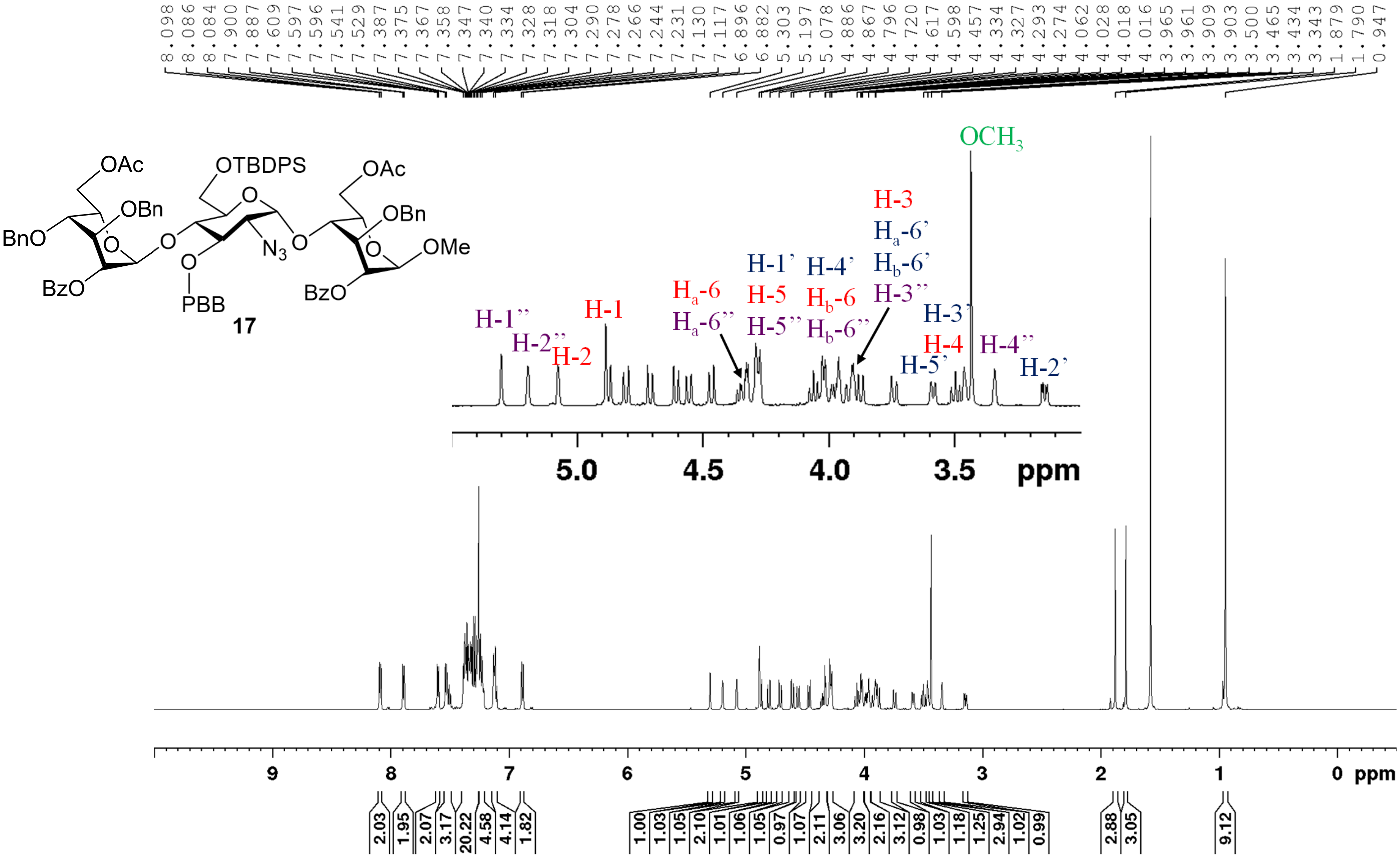
OAc
OTBDPS
OAc
OBn
OBn
BnO
O
N_3
OMe
BzO
BzO
PBB
17
8.098 8.086 8.084 7.900 7.887 7.609 7.597 7.596 7.541 7.529 7.387 7.375 7.367 7.358 7.347 7.340 7.334 7.328 7.318 7.304 7.290 7.278 7.266 7.244 7.231 7.130 7.117 6.896 6.882 5.303 5.197 5.078 4.886 4.867 4.796 4.720 4.617 4.598 4.457 4.334 4.327 4.293 4.274 4.062 4.028 4.018 4.016 3.965 3.961 3.909 3.903 3.500 3.465 3.434 3.343 1.879 1.790 0.947
OCH_3
H-1''
H-2''
H-2
H-1
H_a-6
H_a-6''
H-1'
H-5
H-5''
H-4'
H_b-6
H_b-6''
H-3
H_a-6'
H_b-6'
H-3''
H-3'
H-4
H-5'
H-4''
H-2'
5.0 4.5 4.0 3.5 ppm
9 8 7 6 5 4 3 2 1 0 ppm
2.03 1.95 2.07 3.17 20.22 4.58 4.14 1.82 1.00 1.03 1.05 2.10 1.01 1.06 1.05 0.97 1.07 2.11 3.06 3.20 2.16 3.12 0.98 1.03 1.18 1.25 2.94 1.02 0.99 2.88 3.05 9.12

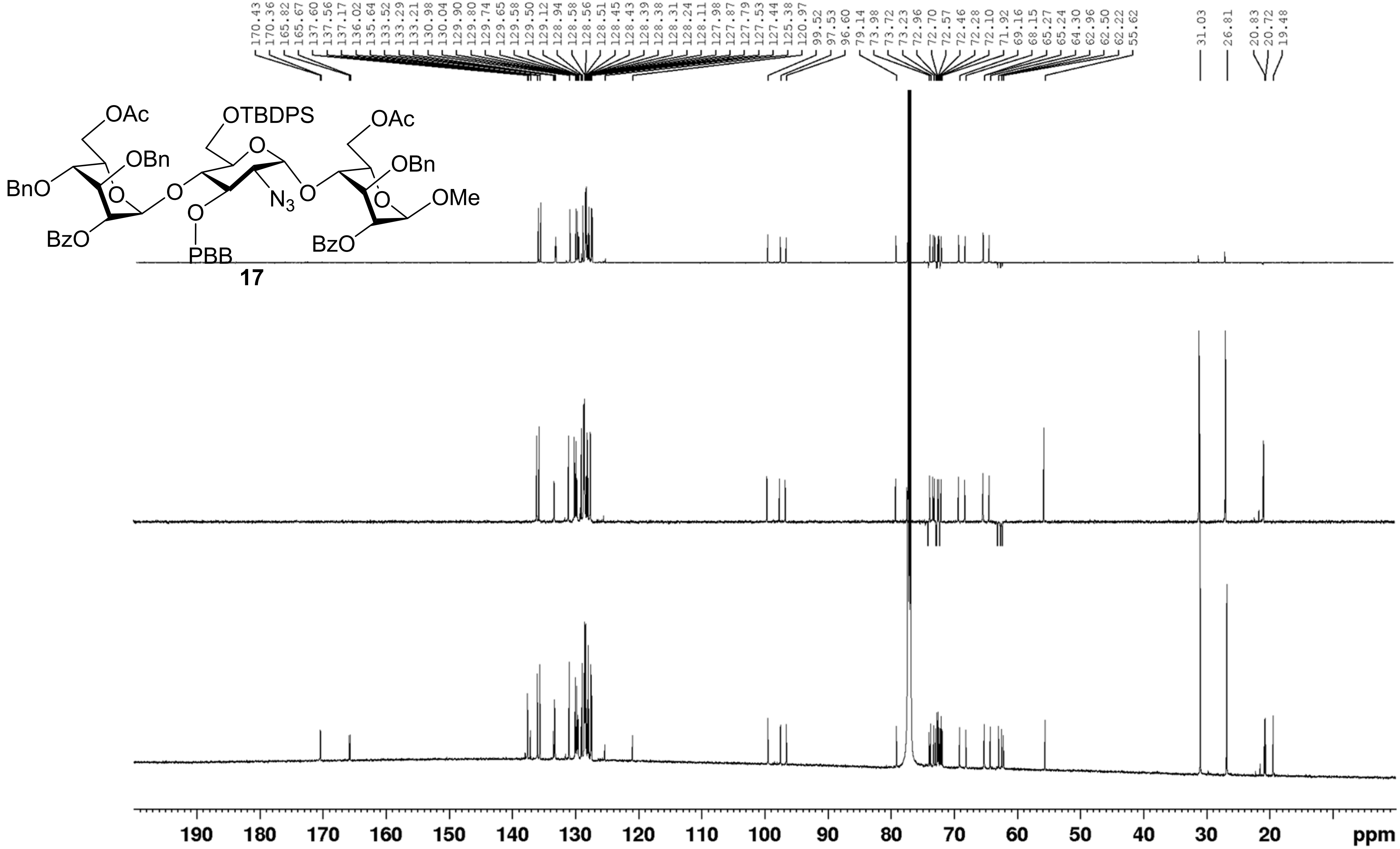
OAc
OTBDPS
OAc
OBn
OBn
BnO
O
O
O
O
N_3
OMe
BzO
BzO
PBB
17
ppm
190
180
170
160
150
140
130
120
110
100
90
80
70
60
50
40
30
20

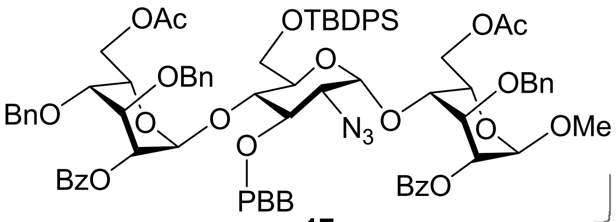

17

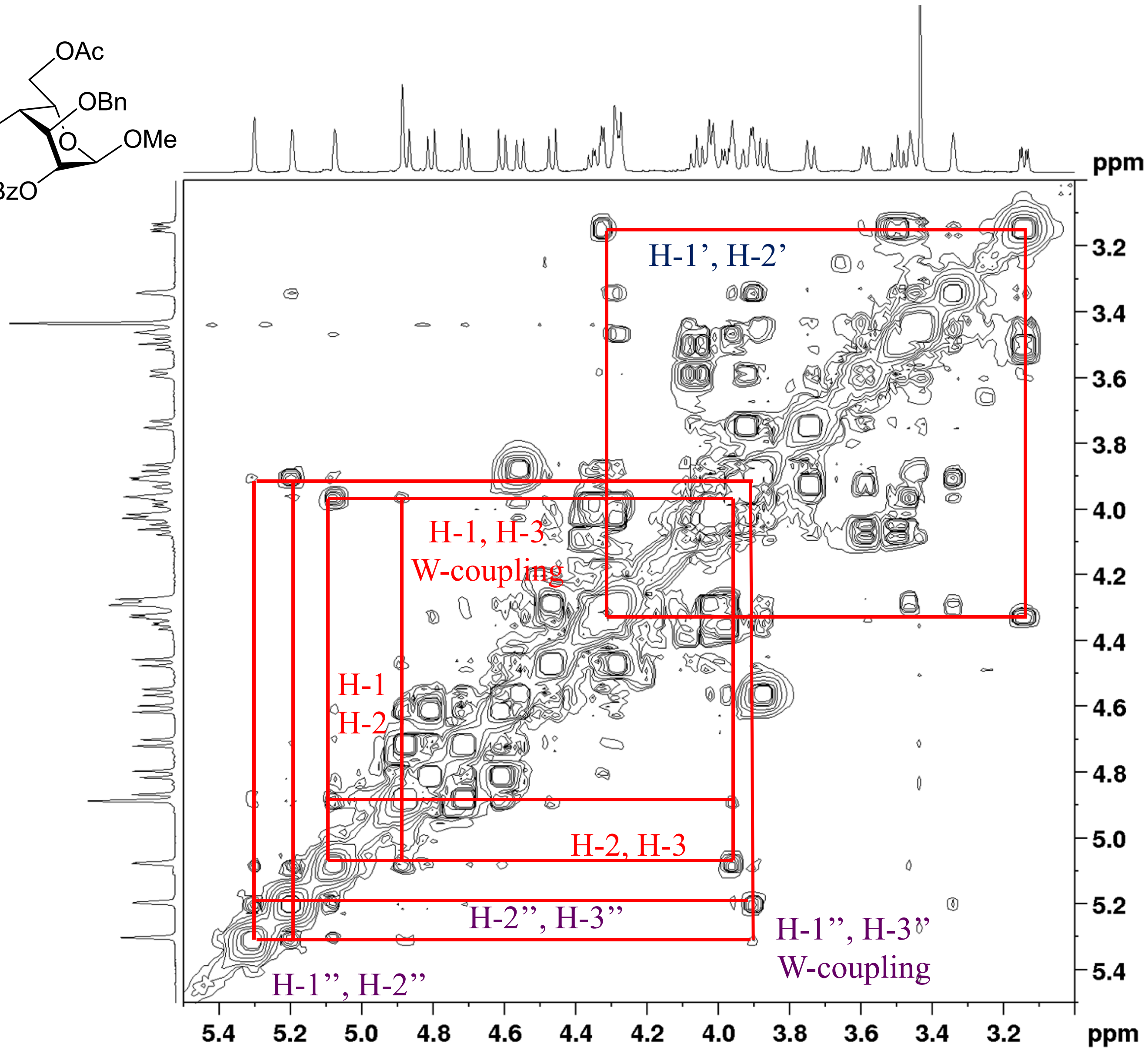

ppm
H-1', H-2'
H-1, H-3
W-coupling
H-1
H-2
H-2, H-3
H-2'', H-3''
H-1'', H-3''
W-coupling
H-1'', H-2''
5.4
5.2
5.0
4.8
4.6
4.4
4.2
4.0
3.8
3.6
3.4
3.2
ppm

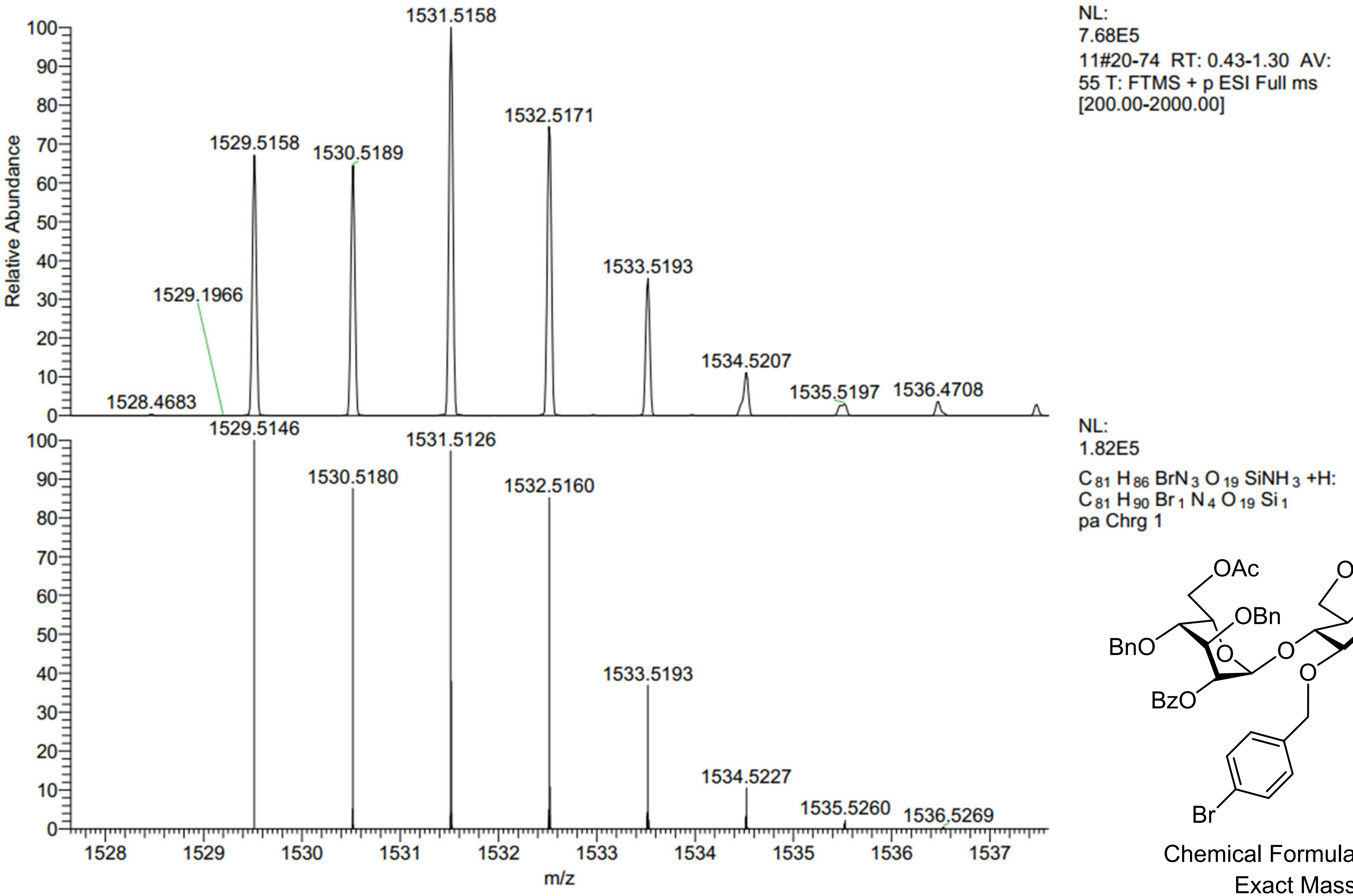

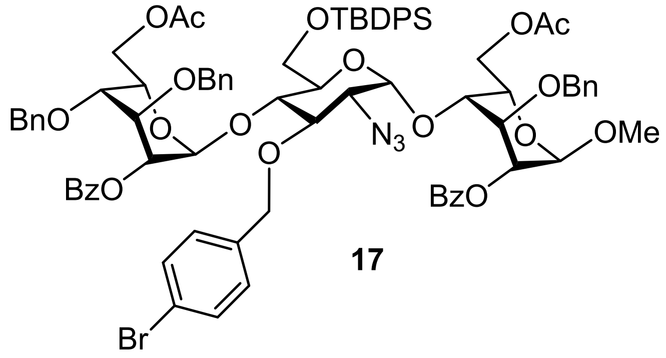

17

Chemical Formula: $C_{81}H_{86}BrN_3O_{19}Si$
Exact Mass: 1511.4808
Molecular Weight: 1513.5700

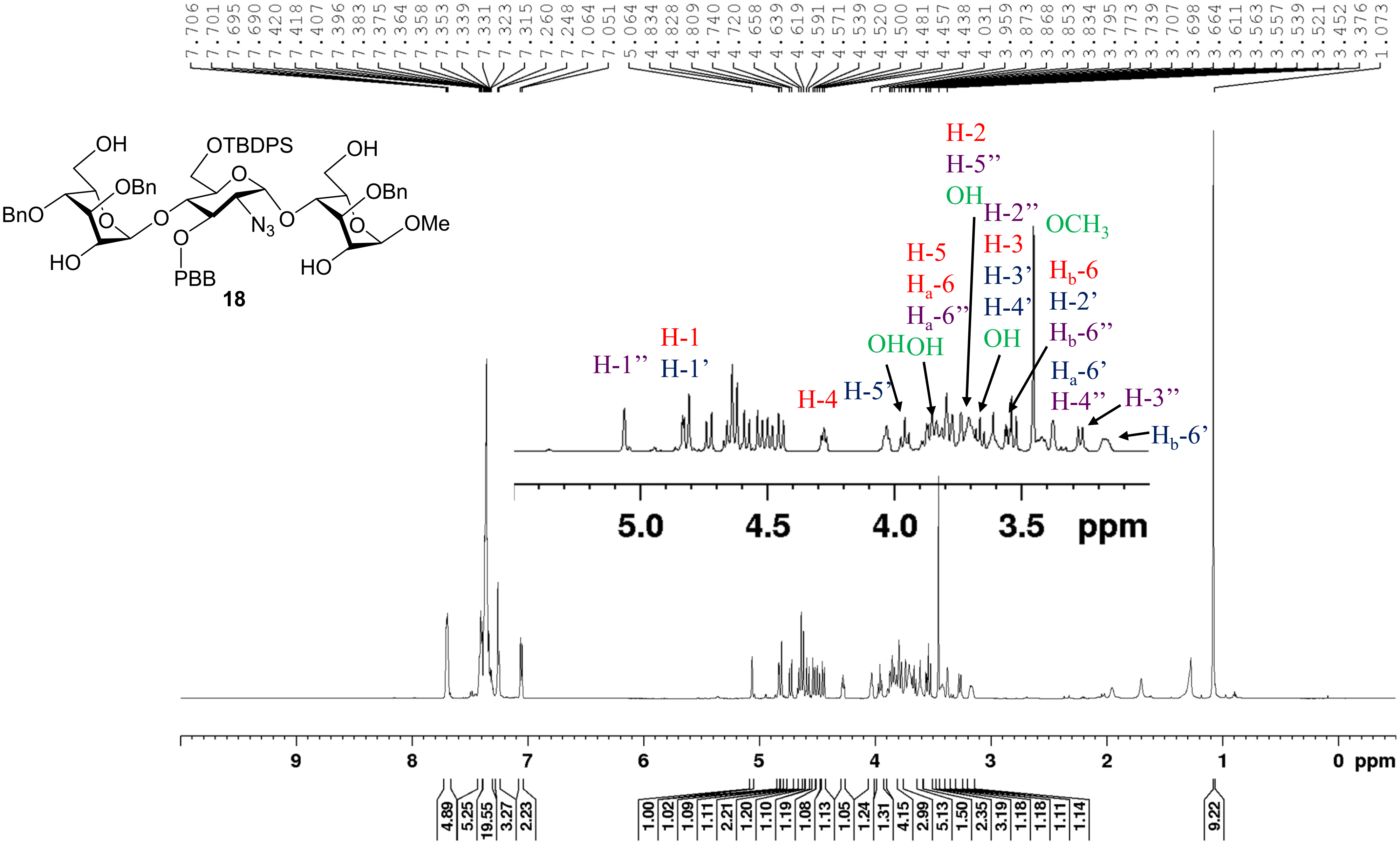
OH
OTBDPS
OH
OBn
OBn
BnO
O
N3
OMe
HO
PBB
HO
18
H-2
H-5''
OH
H-2''
OCH3
H-3
H-5
Ha-6
Ha-6''
H-3'
H-4'
OH
Hb-6
H-2'
Hb-6''
Ha-6'
H-4''
H-3''
Hb-6'
H-1
H-1''
H-1'
H-4
H-5'
OH
OH
5.0
4.5
4.0
3.5
ppm
9
8
7
6
5
4
3
2
1
0
ppm

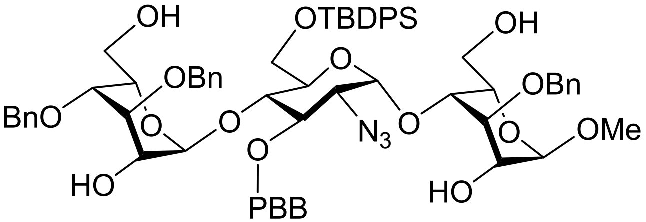
OH
OTBDPS
OH
OBn
OBn
BnO
O
O
O
O
N_3
O
O
OMe
HO
O
HO
PBB

18

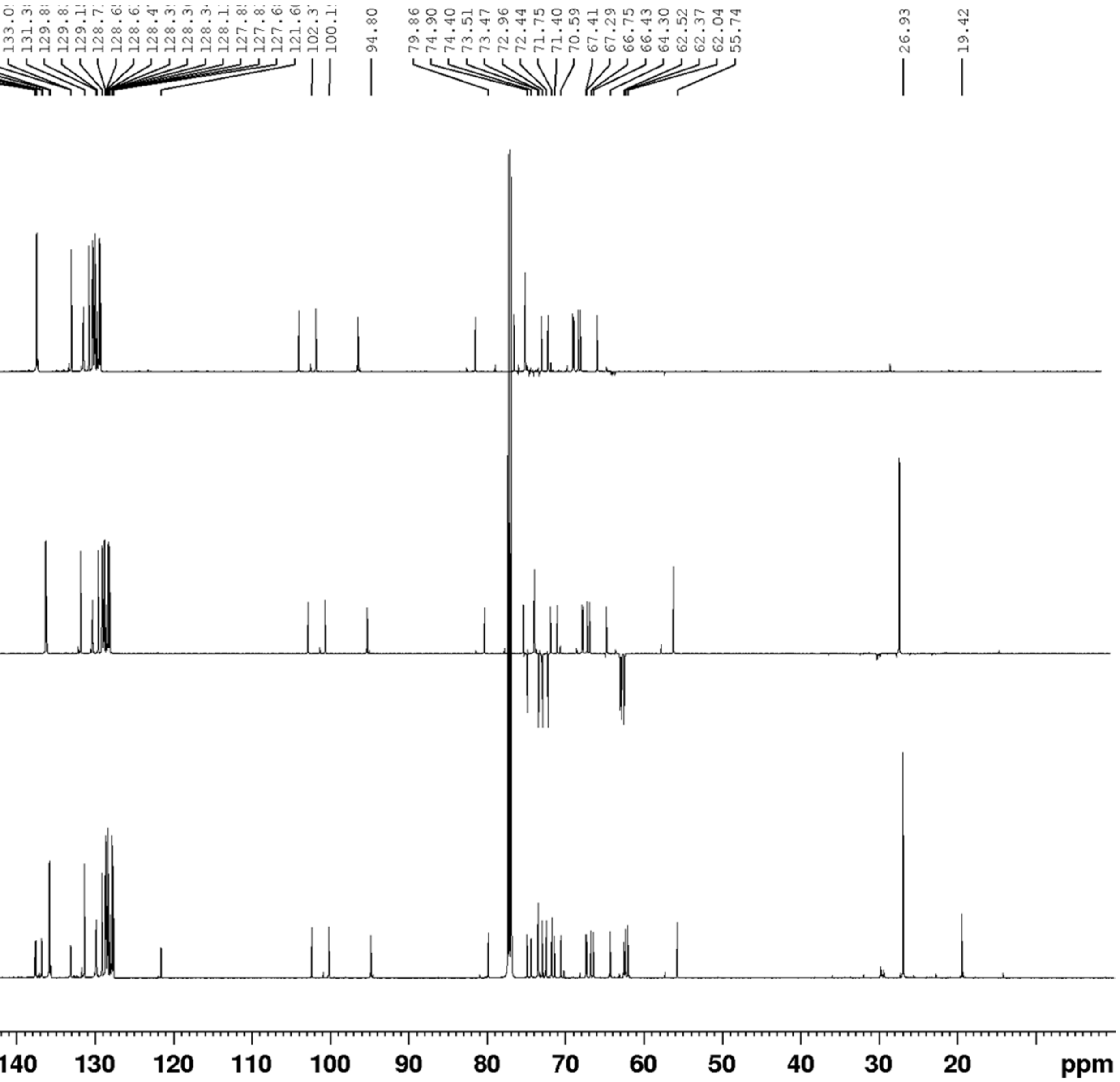
137.6
137.5
136.8
136.7
135.8
135.7
133.1
133.0
131.3
129.8
129.8
129.1
128.7
128.6
128.6
128.4
128.3
128.3
128.3
128.1
127.8
127.8
127.6
121.6
102.3
100.1
94.80
79.86
74.90
74.40
73.51
73.47
72.96
72.44
71.75
71.40
70.59
67.41
67.29
66.75
66.43
64.30
62.52
62.37
62.04
55.74
26.93
19.42
190
180
170
160
150
140
130
120
110
100
90
80
70
60
50
40
30
20
ppm

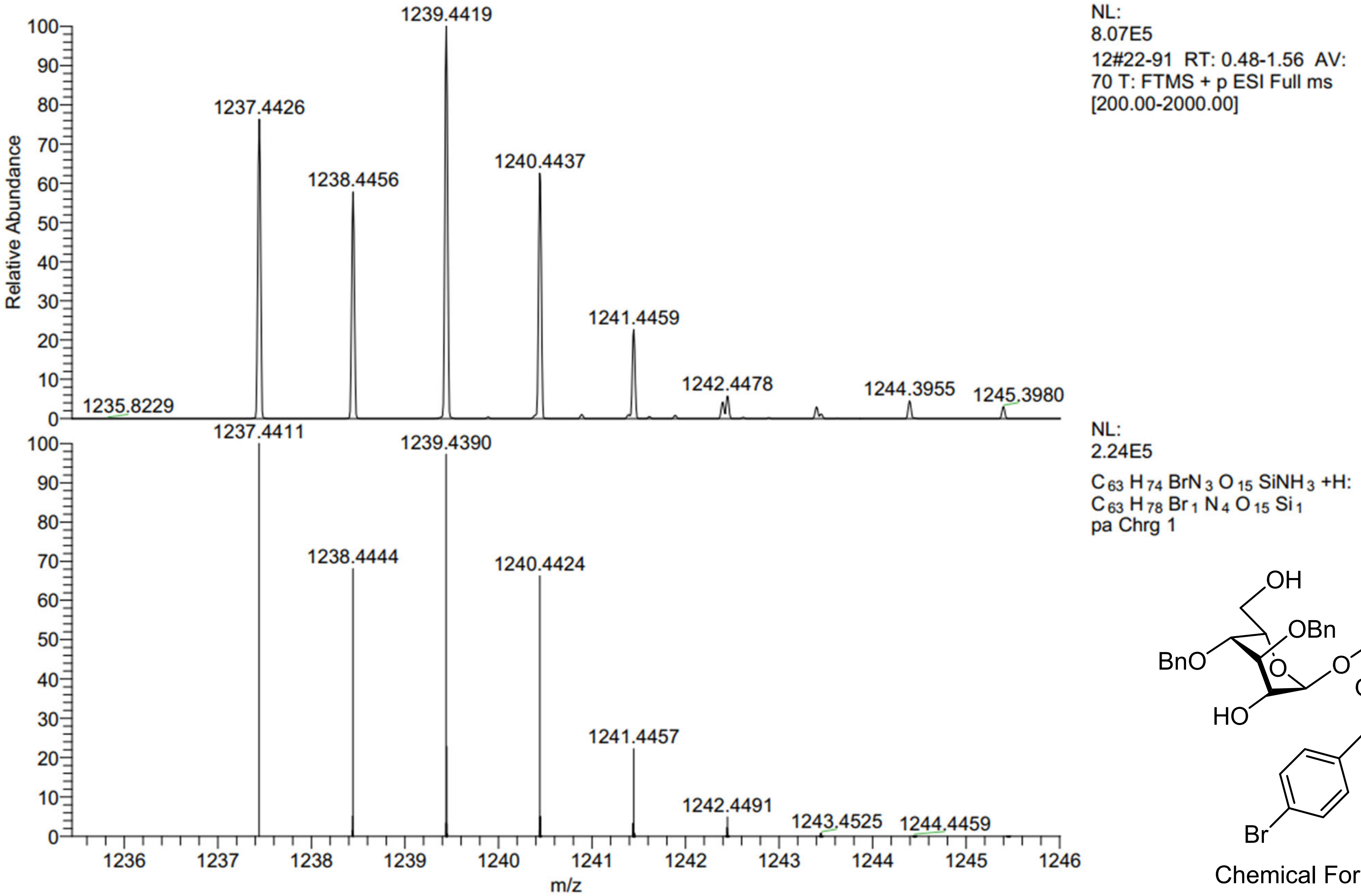

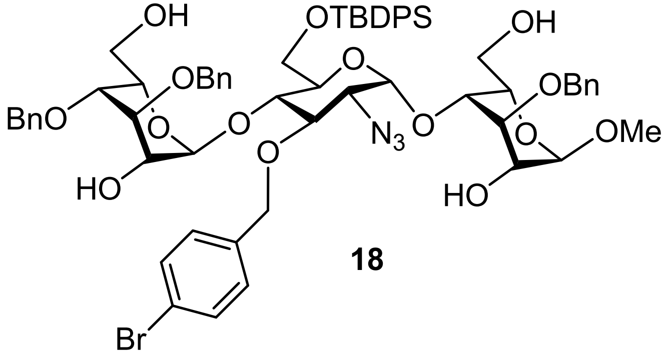

18

Chemical Formula: $C_{63}H_{74}BrN_3O_{15}Si$

Exact Mass: 1219.4073

Molecular Weight: 1221.2800

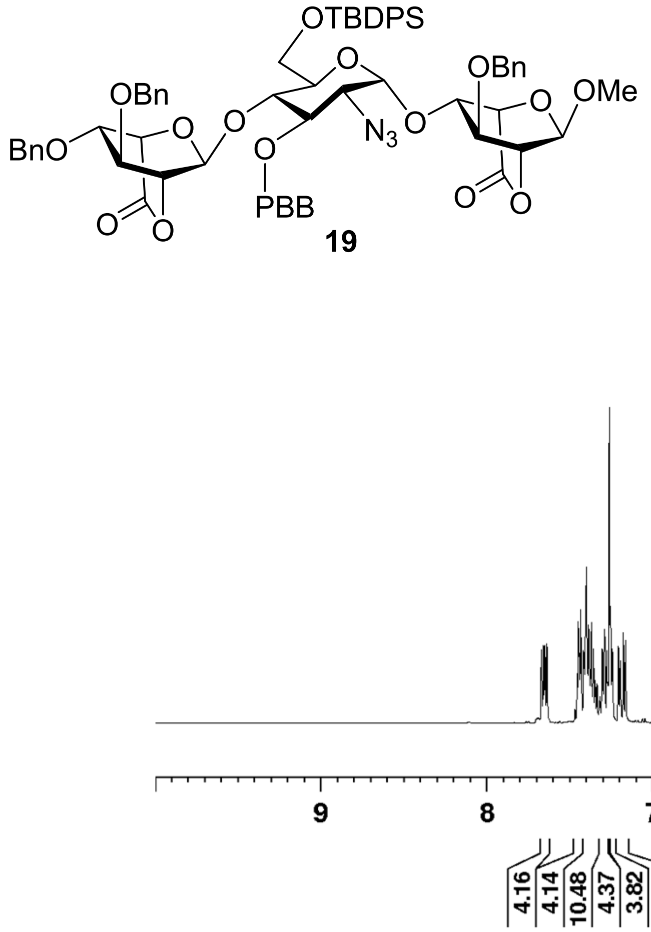

19

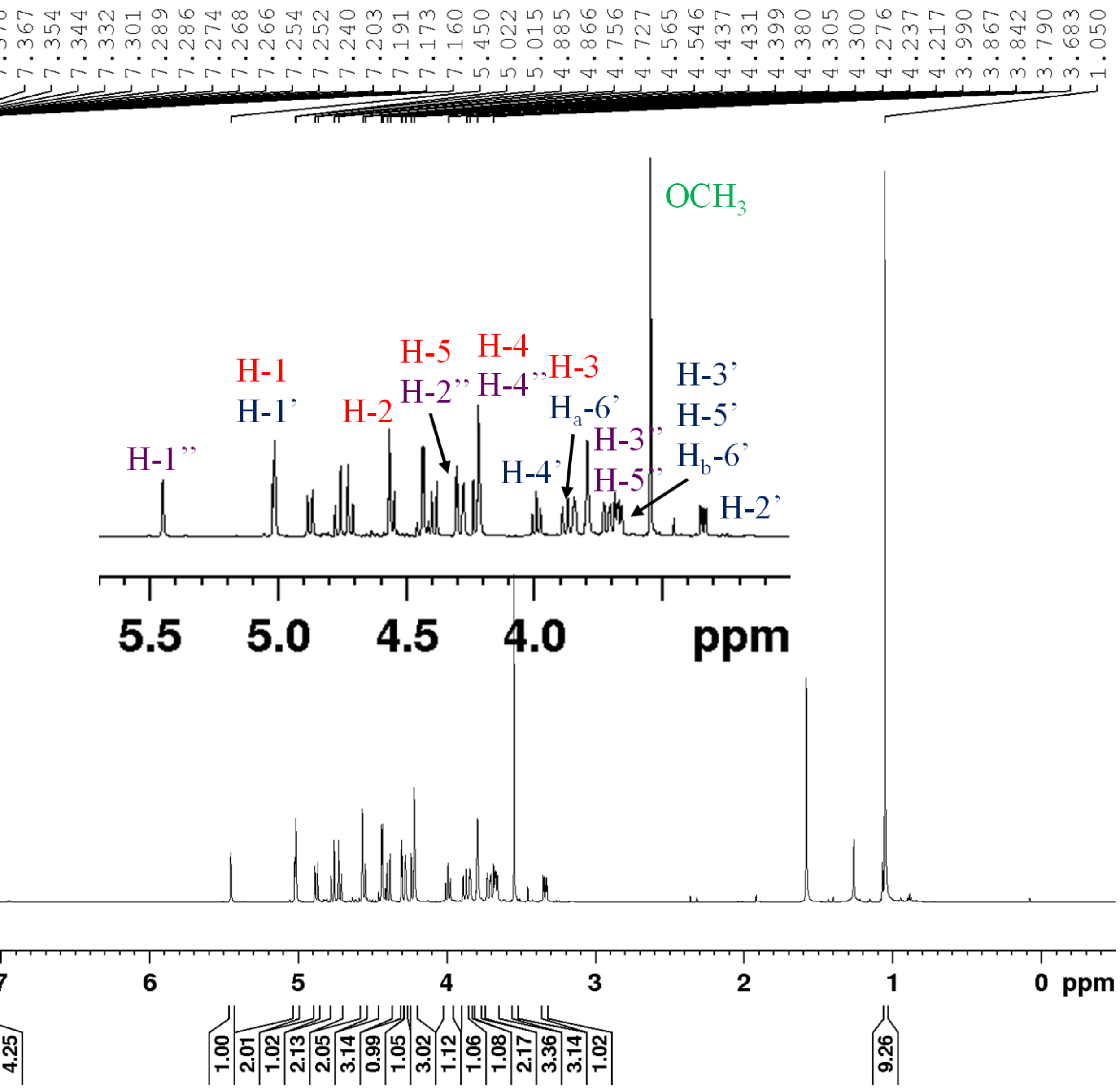

OCH$_3$
H-1''
H-1
H-1'
H-2
H-5
H-2''
H-4
H-4''
H-3
H$_a$-6'
H-4'
H-3''
H-5''
H-3'
H-5'
H$_b$-6'
H-2'
5.5 5.0 4.5 4.0 ppm
9 8 7 6 5 4 3 2 1 0 ppm

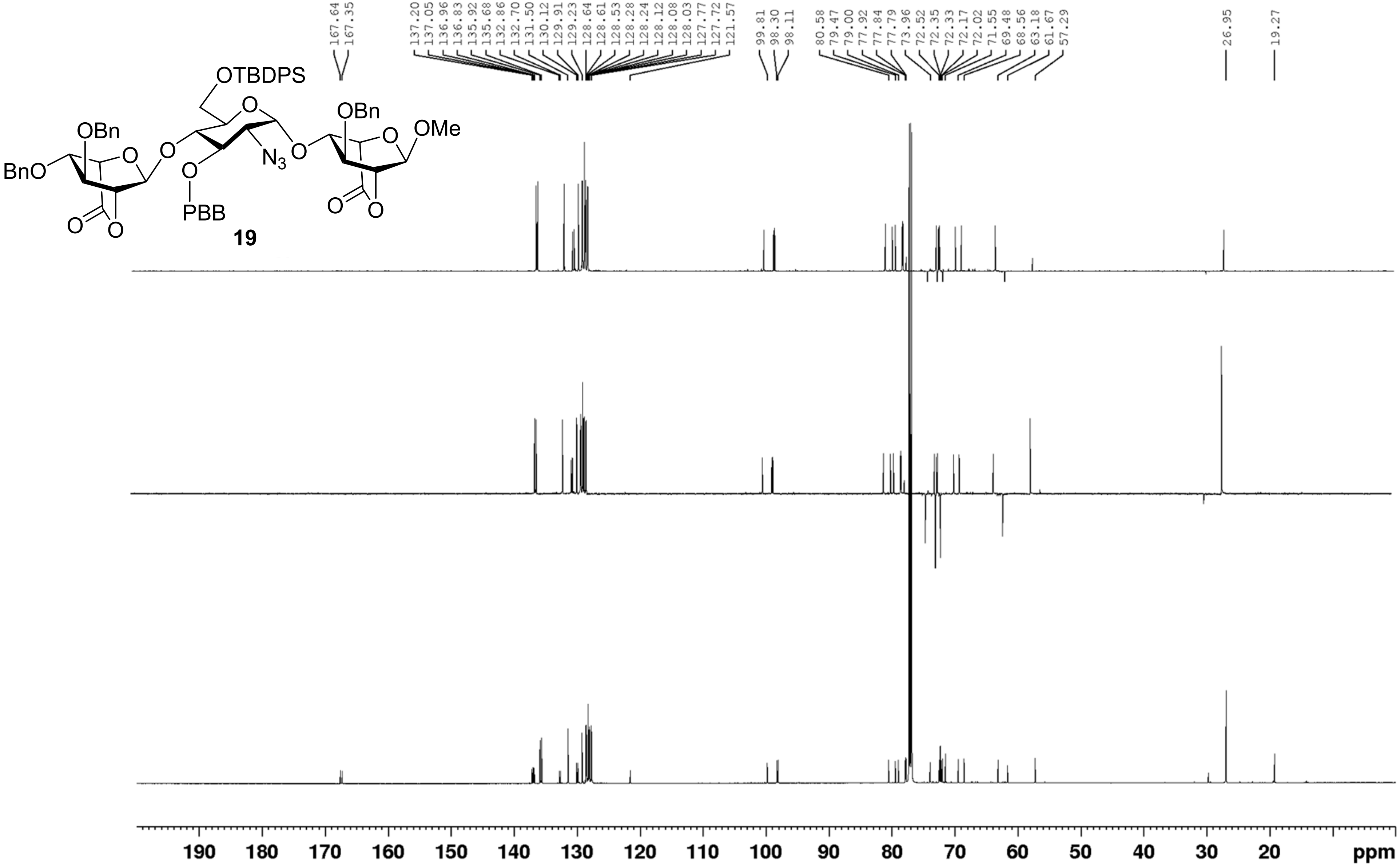
OTBDPS
OBn
OBn
OMe
BnO
O
N3
PBB
O
19
167.64
167.35
137.20
137.05
136.96
136.83
135.92
135.68
132.86
132.70
131.50
130.12
129.91
129.23
128.64
128.61
128.53
128.28
128.24
128.12
128.08
128.03
127.77
127.72
121.57
99.81
98.30
98.11
80.58
79.47
79.00
77.92
77.84
77.79
73.96
72.52
72.35
72.33
72.17
72.02
71.55
69.48
68.56
63.18
61.67
57.29
26.95
19.27
190
180
170
160
150
140
130
120
110
100
90
80
70
60
50
40
30
20
ppm

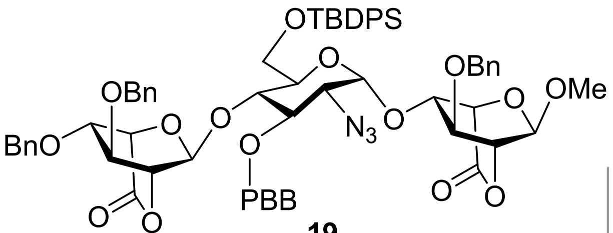
OTBDPS
OBn
OBn
OMe
BnO
N3
PBB
19

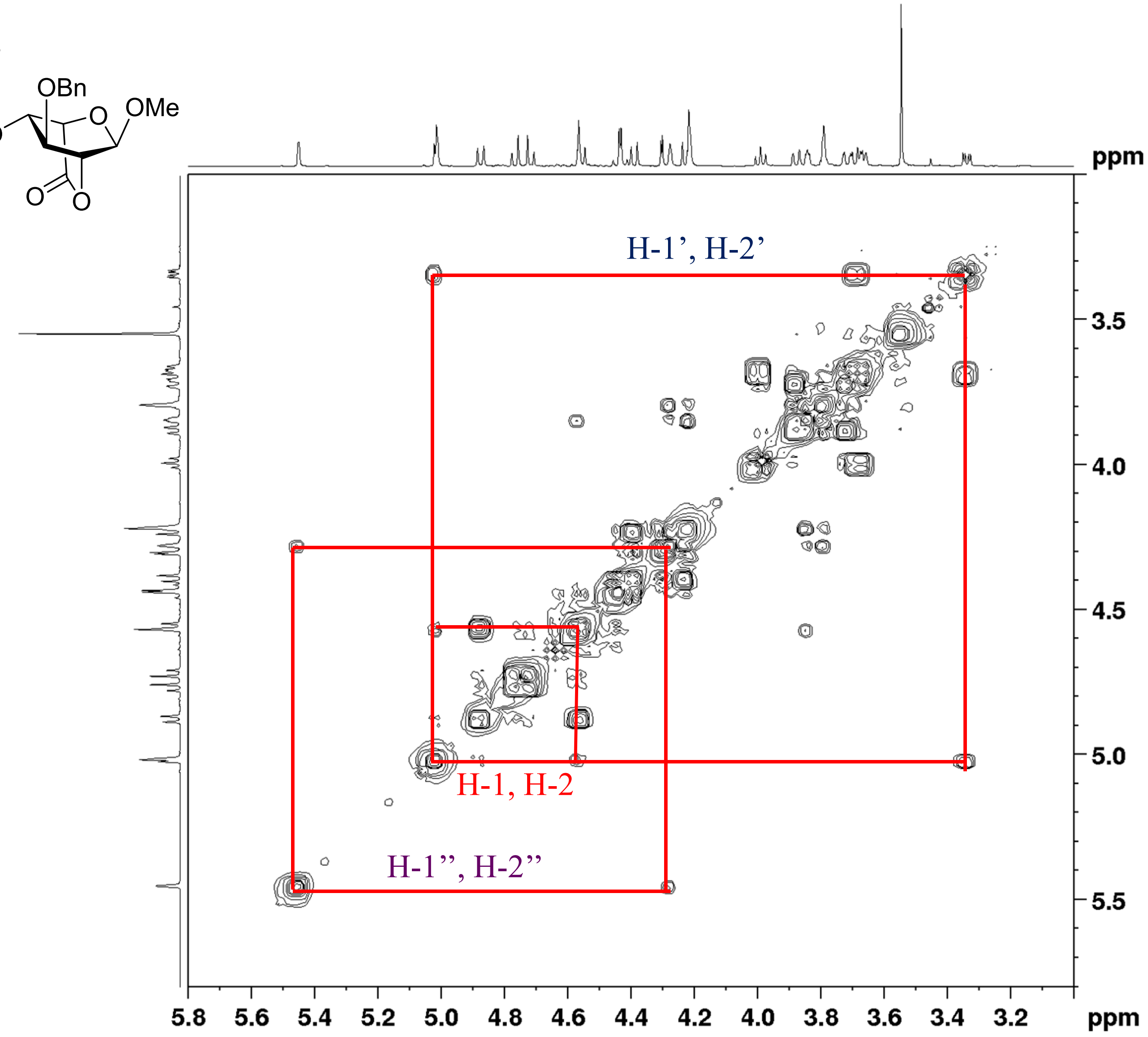
H-1’, H-2’
H-1, H-2
H-1’’, H-2’’
ppm
5.8 5.6 5.4 5.2 5.0 4.8 4.6 4.4 4.2 4.0 3.8 3.6 3.4 3.2
ppm
3.5
4.0
4.5
5.0
5.5

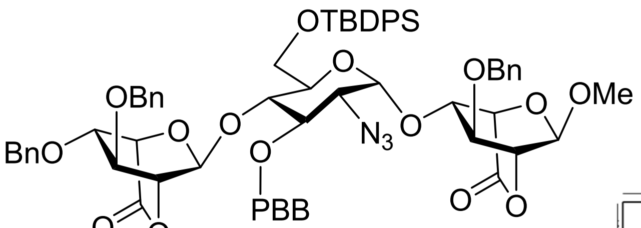
OTBDPS
OBn
OBn
OMe
BnO
N3
PBB

19

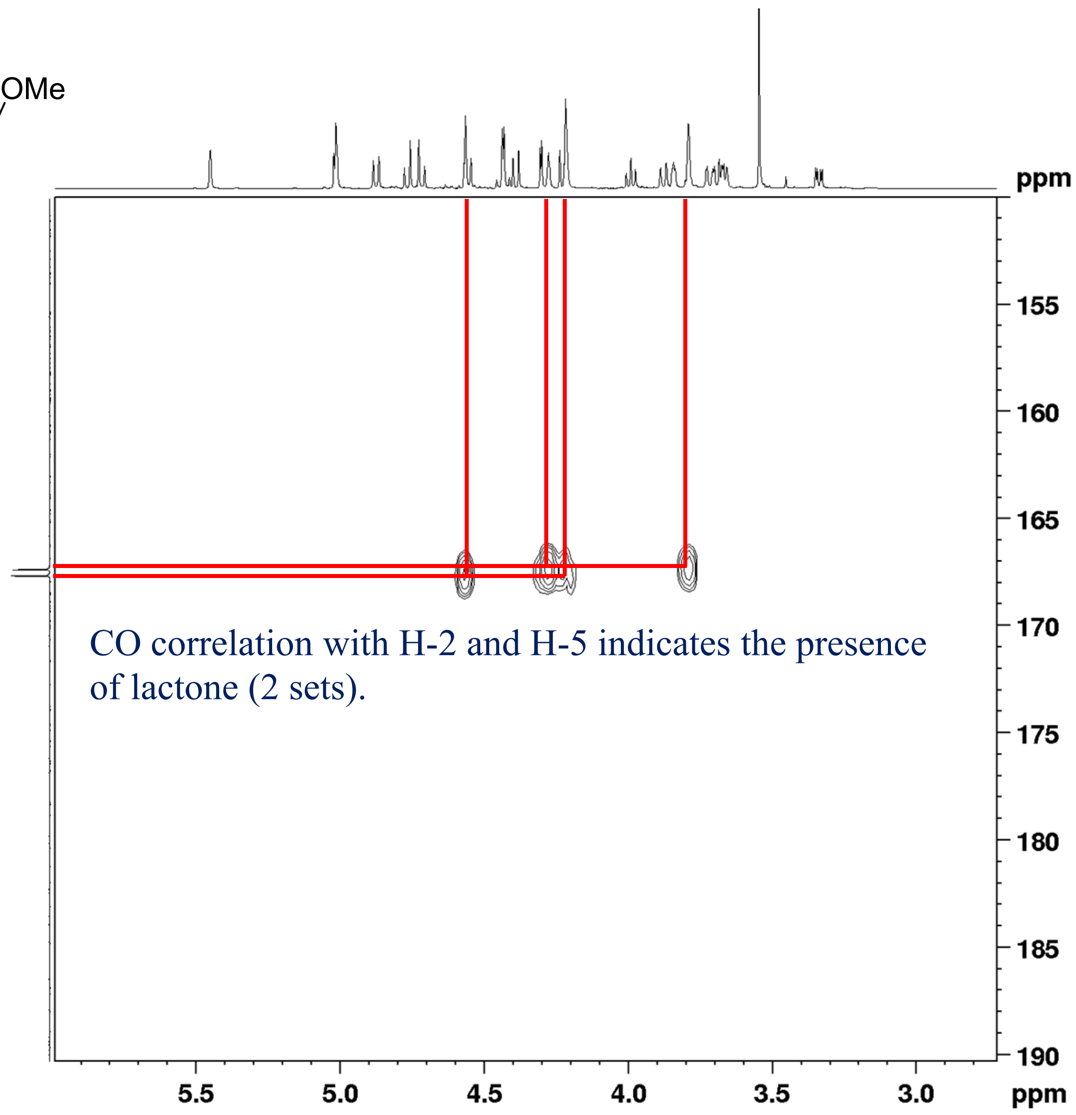
ppm
155
160
165
170
175
180
185
190
5.5
5.0
4.5
4.0
3.5
3.0
ppm
CO correlation with H-2 and H-5 indicates the presence of lactone (2 sets).

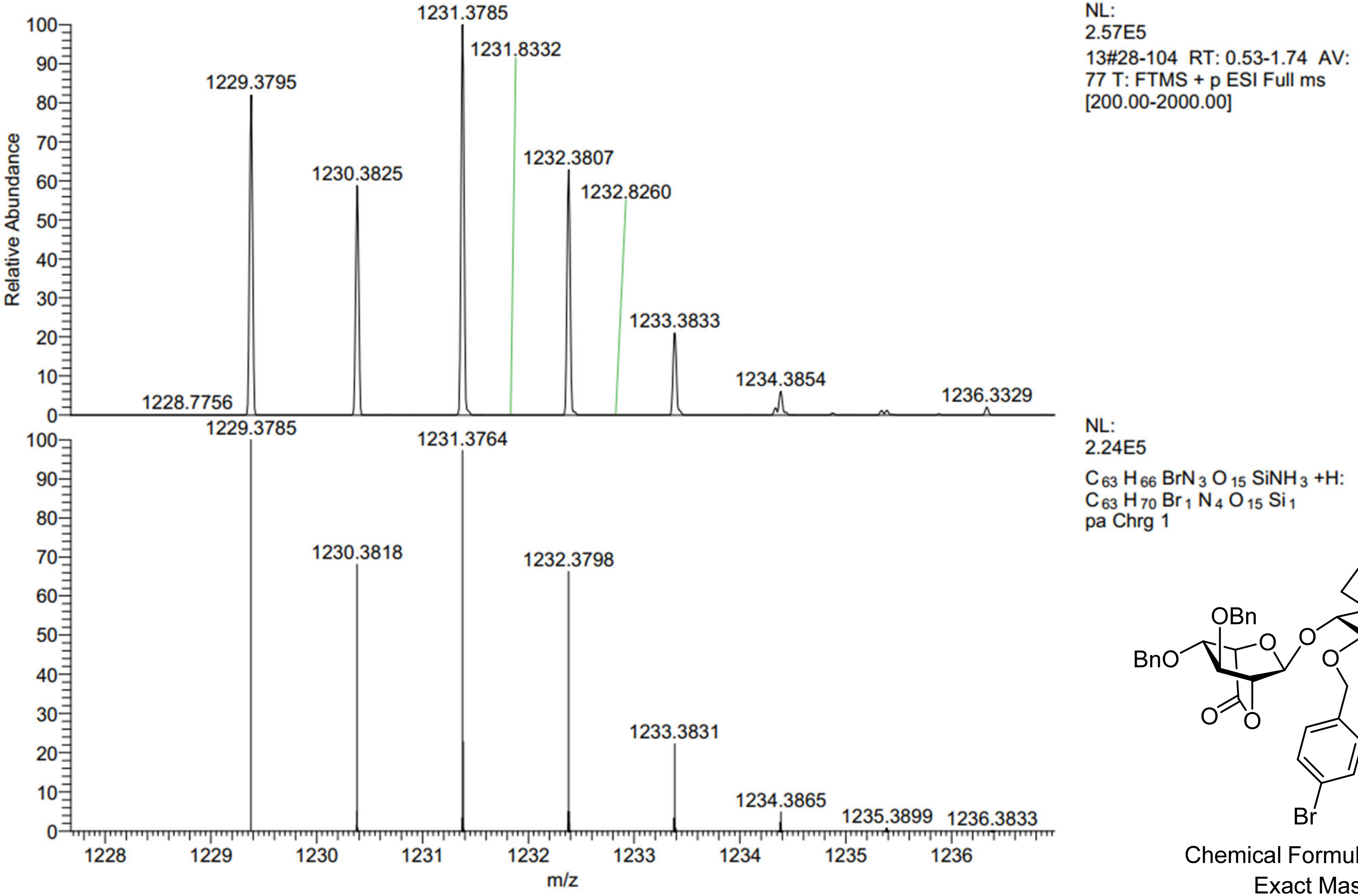

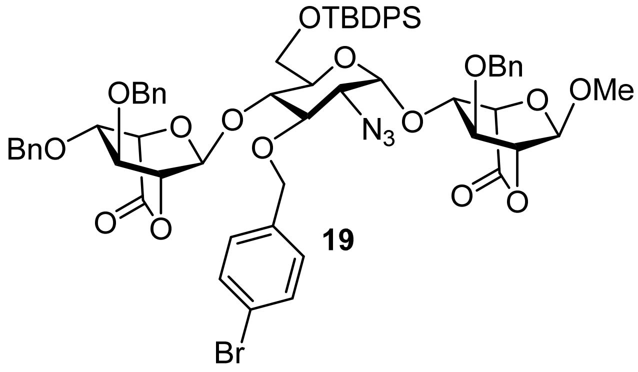

Chemical Formula: $C_{63}H_{66}BrN_3O_{15}Si$

Exact Mass: 1211.3447

Molecular Weight: 1213.2160

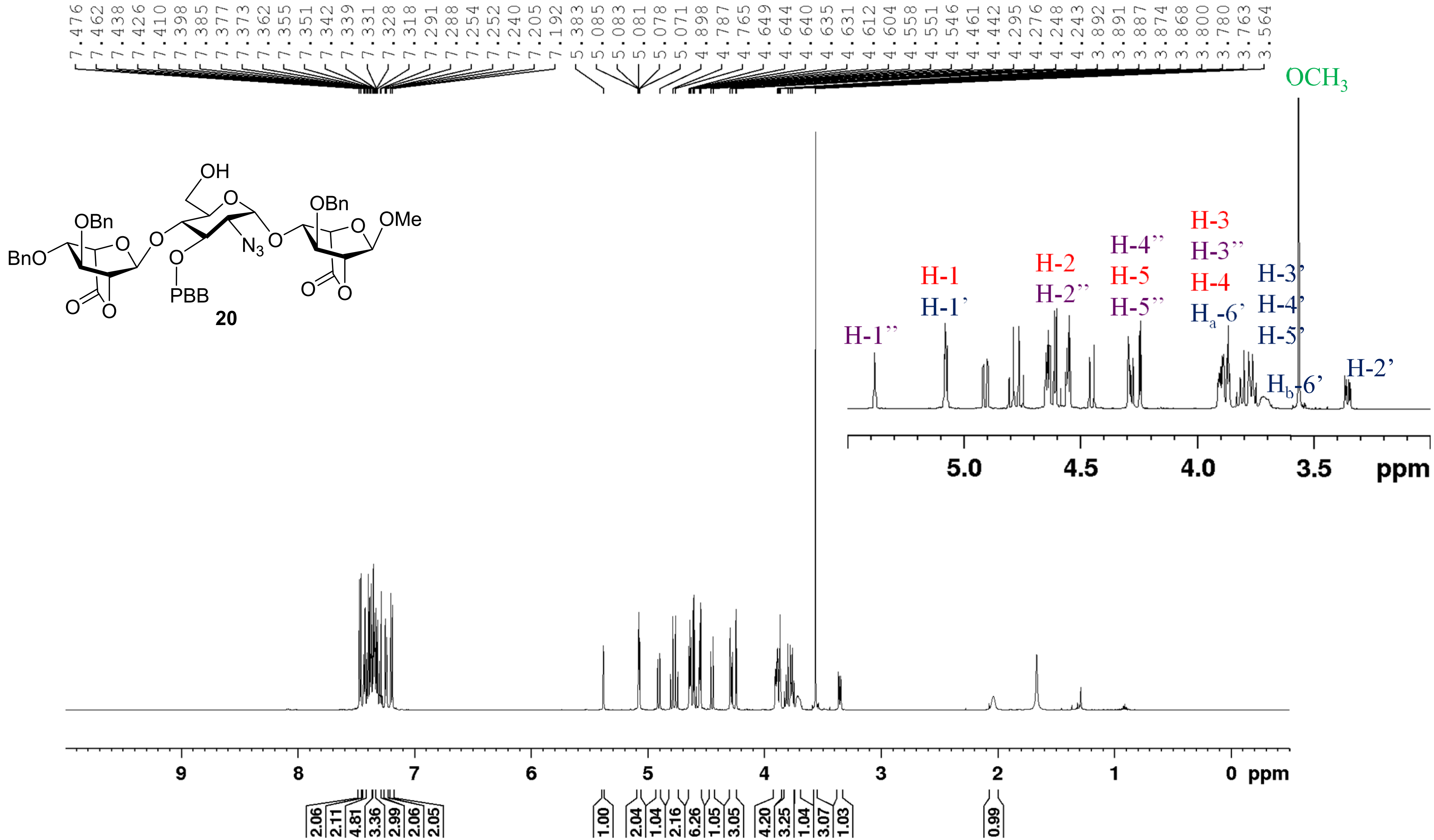

OCH3
H-1''
H-1
H-1'
H-2
H-2''
H-4''
H-5
H-5''
H-3
H-3''
H-4
Ha-6'
H-3'
H-4'
H-5'
Hb-6'
H-2'
OH
OBn
OMe
BnO
N3
PBB
20
ppm

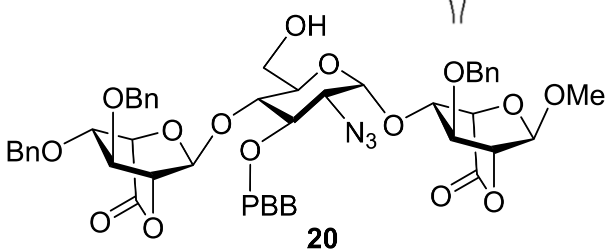

20

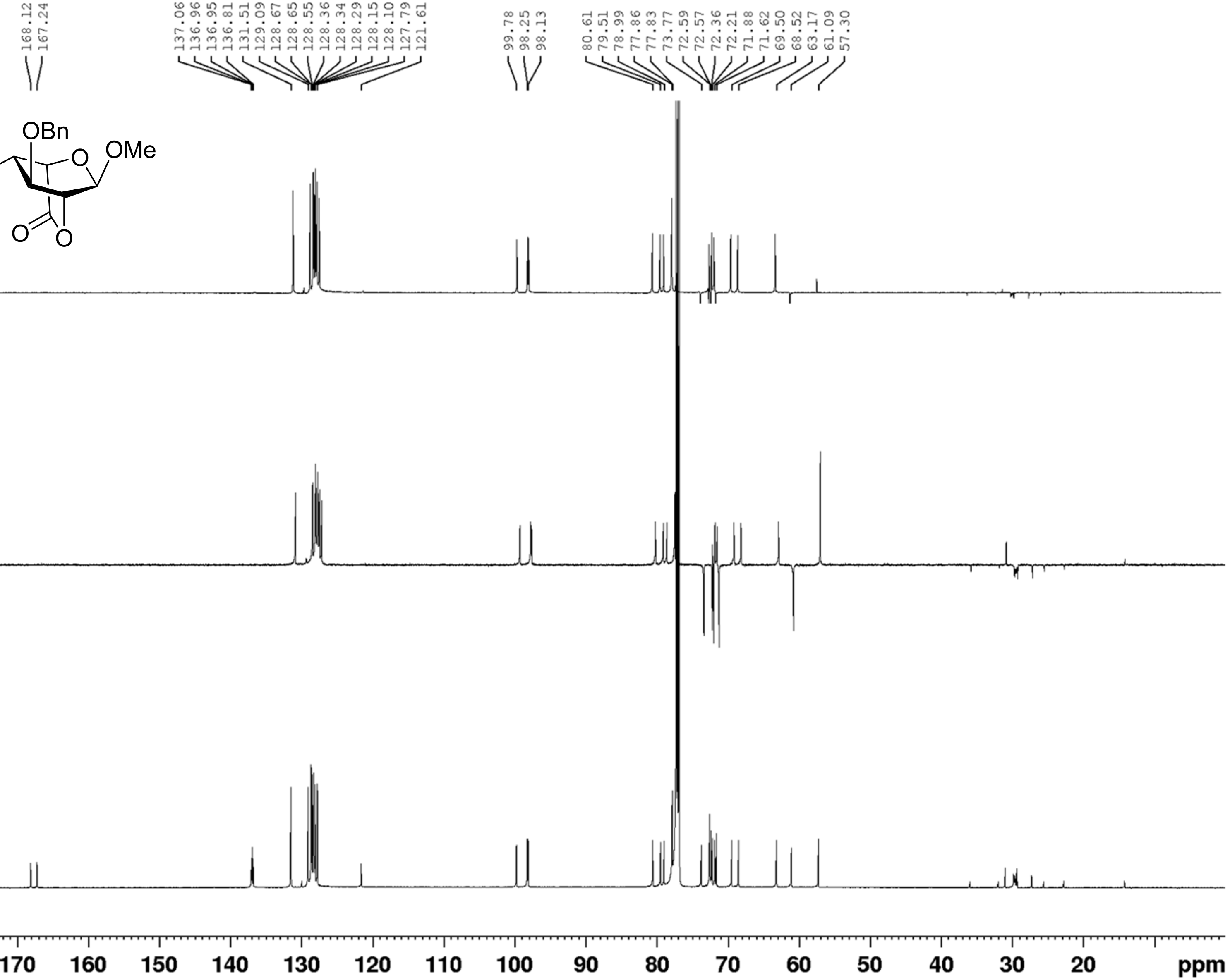
168.12
167.24
137.06
136.96
136.95
136.81
131.51
129.09
128.67
128.65
128.55
128.36
128.34
128.29
128.15
128.10
127.79
121.61
99.78
98.25
98.13
80.61
79.51
78.99
77.86
77.83
73.77
72.59
72.57
72.36
72.21
71.88
71.62
69.50
68.52
63.17
61.09
57.30
190 180 170 160 150 140 130 120 110 100 90 80 70 60 50 40 30 20 ppm

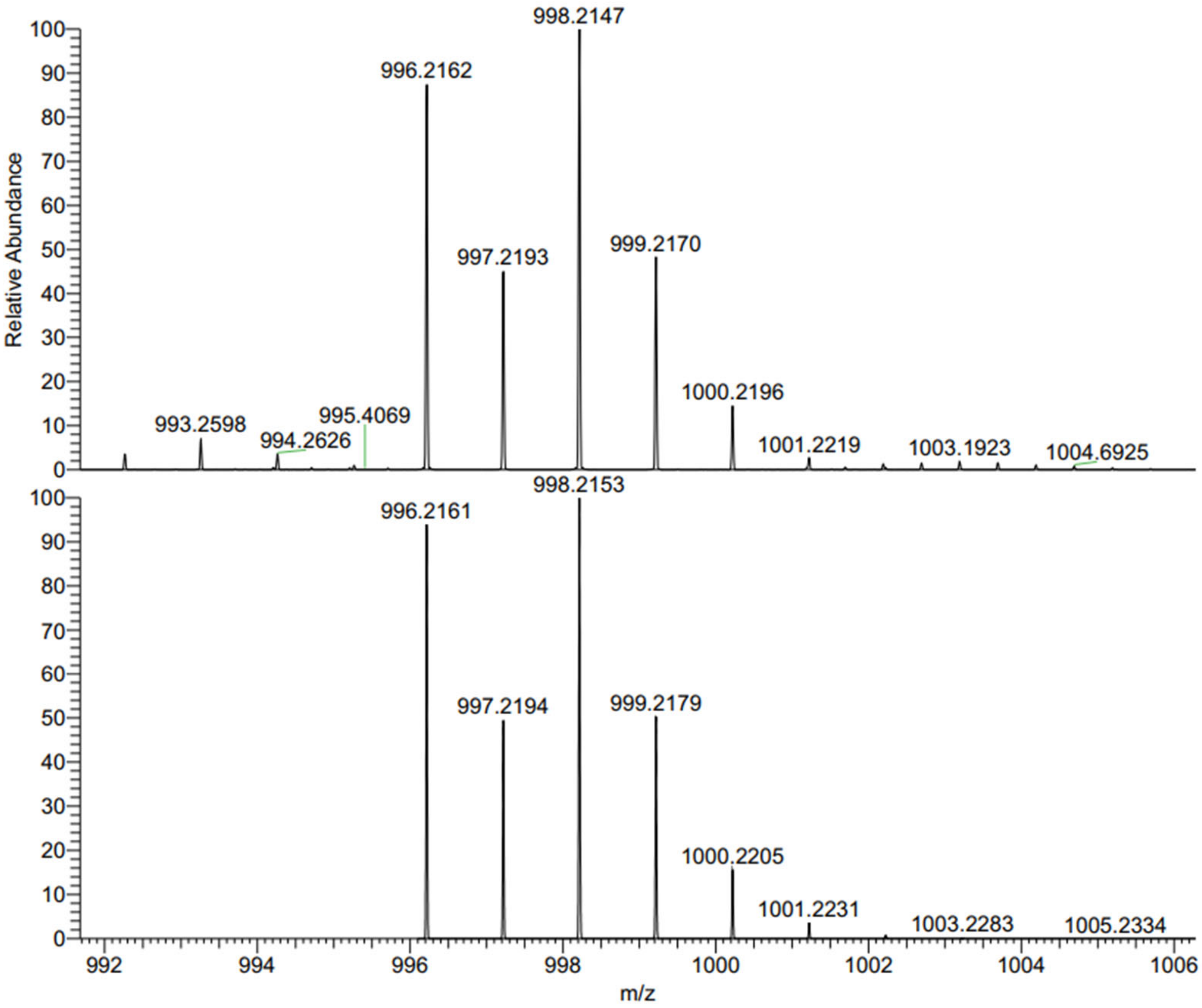

NL:
4.10E6
KYT-110#83-92 RT:
1.53-1.69 AV: 10 T: FTMS +
p ESI Full ms
[200.00-2000.00]

NL:
7.24E3
$C_{47}H_{48}BrN_3O_{15}$ +Na:
$C_{47}H_{48}Br_1N_3O_{15}Na_1$
p (gss, s /p:40) Chrg 1
R: 60000 Res .Pwr . @FWHM

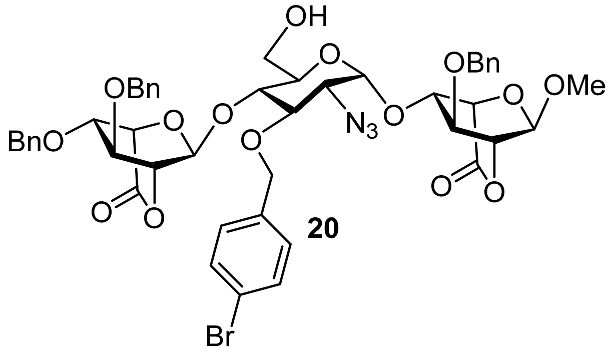

20

Chemical Formula: $C_{47}H_{48}BrN_3O_{15}$
Exact Mass: 973.2269
Molecular Weight: 974.8110

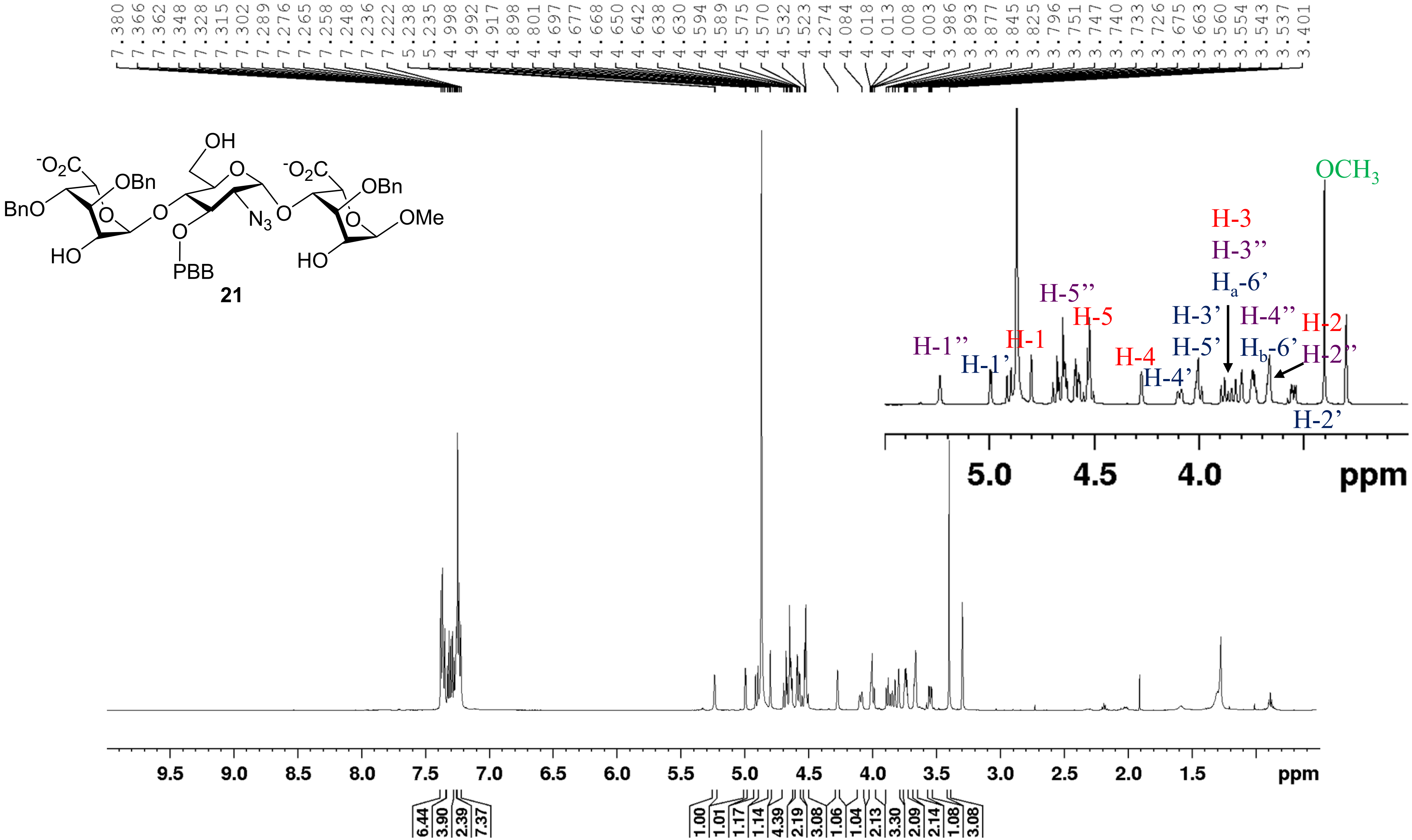

OH
-O2C
OBn
BnO
O
HO
PBB
N3
OMe
21
7.380 7.366 7.362 7.348 7.328 7.315 7.302 7.289 7.276 7.265 7.258 7.248 7.236 7.222 5.238 5.235 4.998 4.992 4.917 4.898 4.801 4.697 4.677 4.668 4.650 4.642 4.638 4.630 4.594 4.589 4.575 4.570 4.532 4.523 4.274 4.084 4.018 4.013 4.008 4.003 3.986 3.893 3.877 3.845 3.825 3.796 3.751 3.747 3.740 3.733 3.726 3.675 3.663 3.560 3.554 3.543 3.537 3.401
OCH3
H-3
H-3''
Ha-6'
H-5''
H-5
H-3'
H-5'
H-4''
Hb-6'
H-2
H-2''
H-1''
H-1'
H-1
H-4
H-4'
H-2'
5.0 4.5 4.0 ppm
9.5 9.0 8.5 8.0 7.5 7.0 6.5 6.0 5.5 5.0 4.5 4.0 3.5 3.0 2.5 2.0 1.5 ppm
6.44 3.90 2.39 7.37
1.00 1.01 1.17 1.14 4.39 2.19 3.08 1.06 1.04 2.13 3.30 2.09 2.14 1.08 3.08

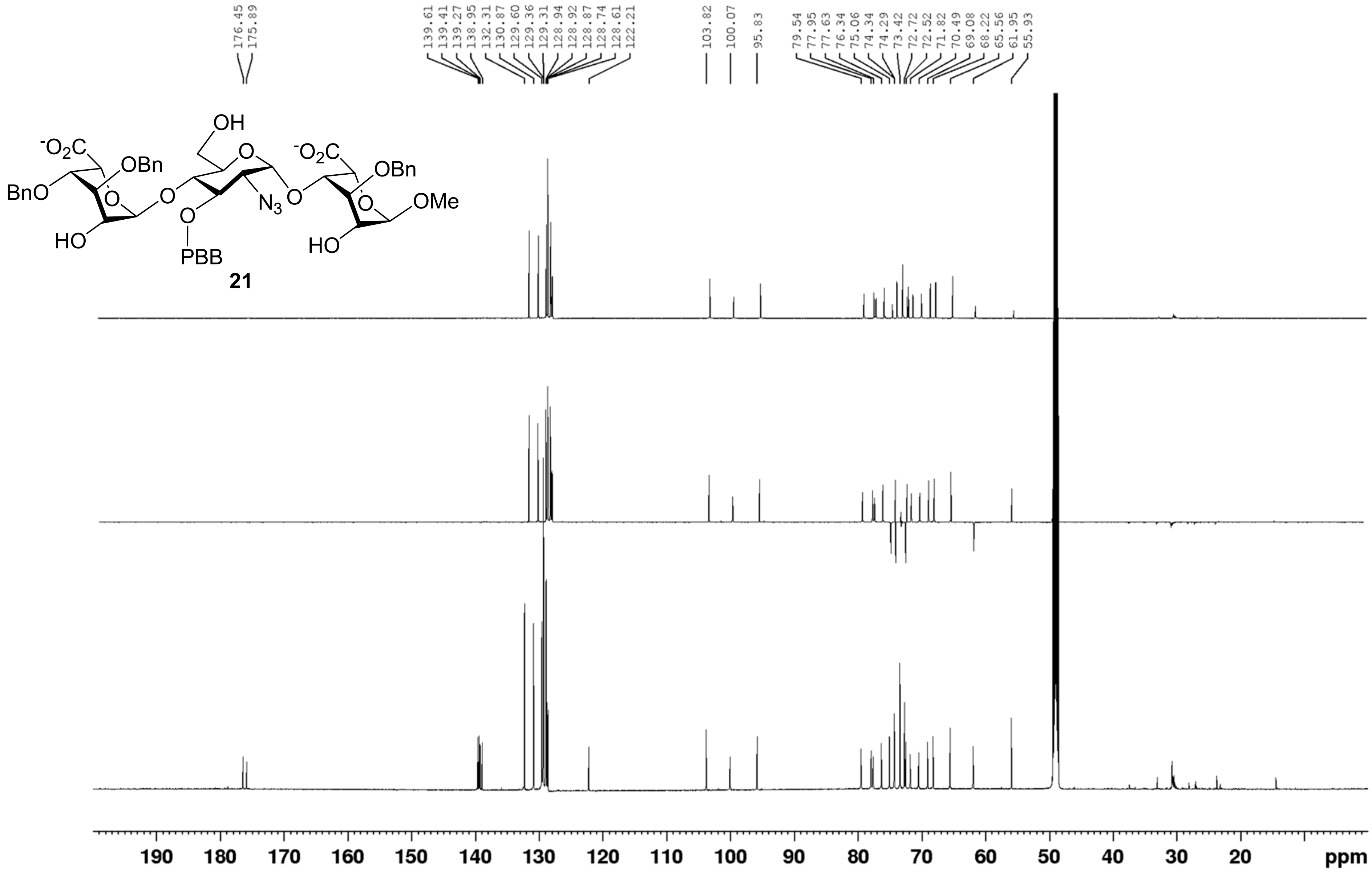
176.45
175.89
139.61
139.41
139.27
138.95
132.31
130.87
129.60
129.36
129.31
128.94
128.92
128.87
128.74
128.61
122.21
103.82
100.07
95.83
79.54
77.95
77.63
76.34
75.06
74.34
74.29
73.42
72.72
72.52
71.82
70.49
69.08
68.22
65.56
61.95
55.93
OH
$^{-}O_2C$
OBn
BnO
O
N_3
OMe
HO
PBB
21
190 180 170 160 150 140 130 120 110 100 90 80 70 60 50 40 30 20 ppm

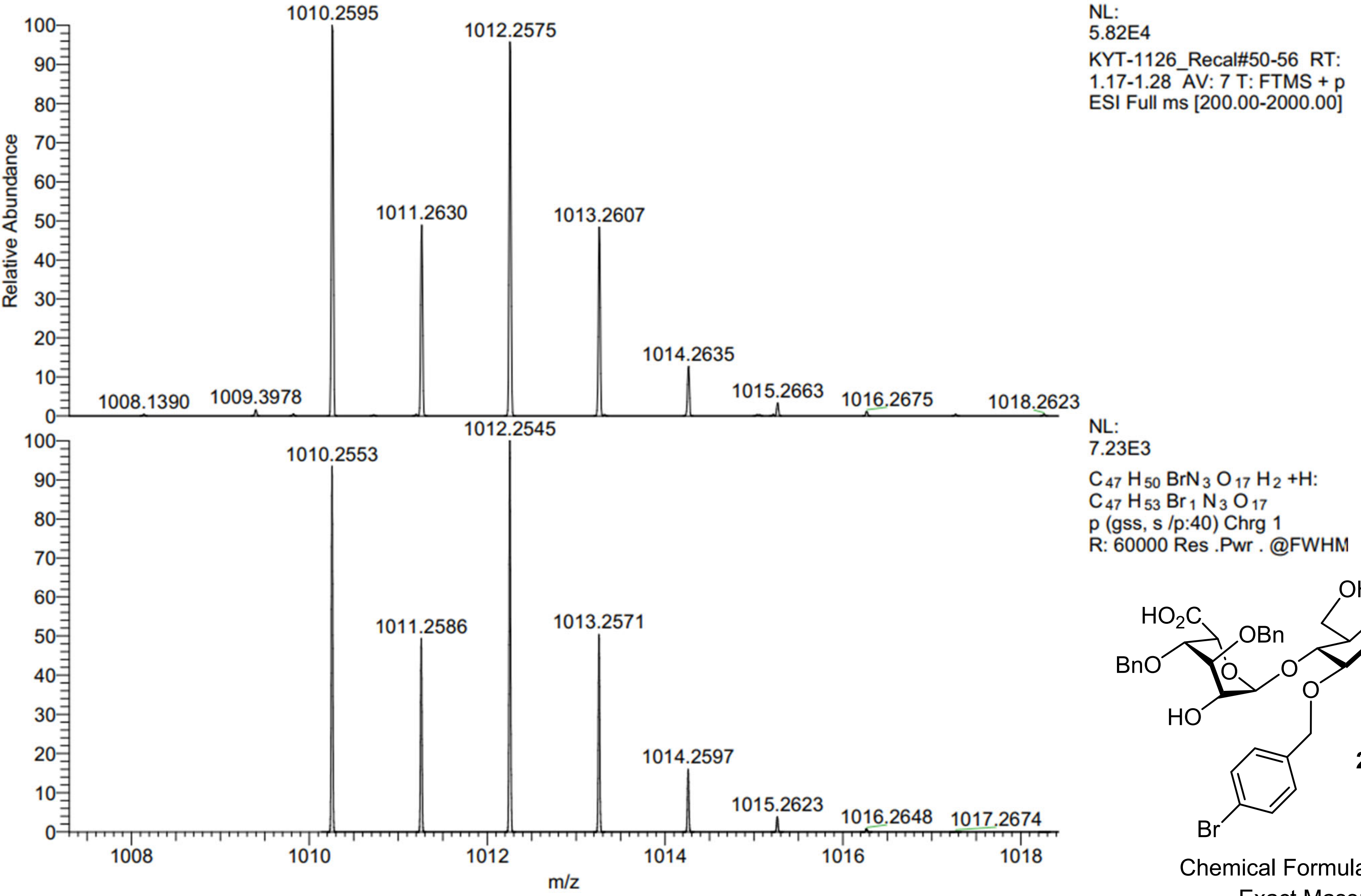

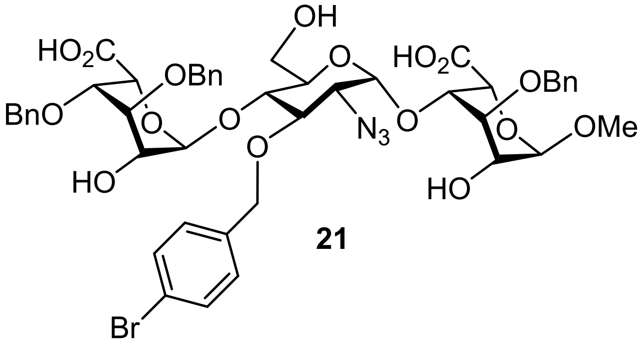

21

Chemical Formula: $C_{47}H_{52}BrN_3O_{17}$
Exact Mass: 1009.2480
Molecular Weight: 1010.8410

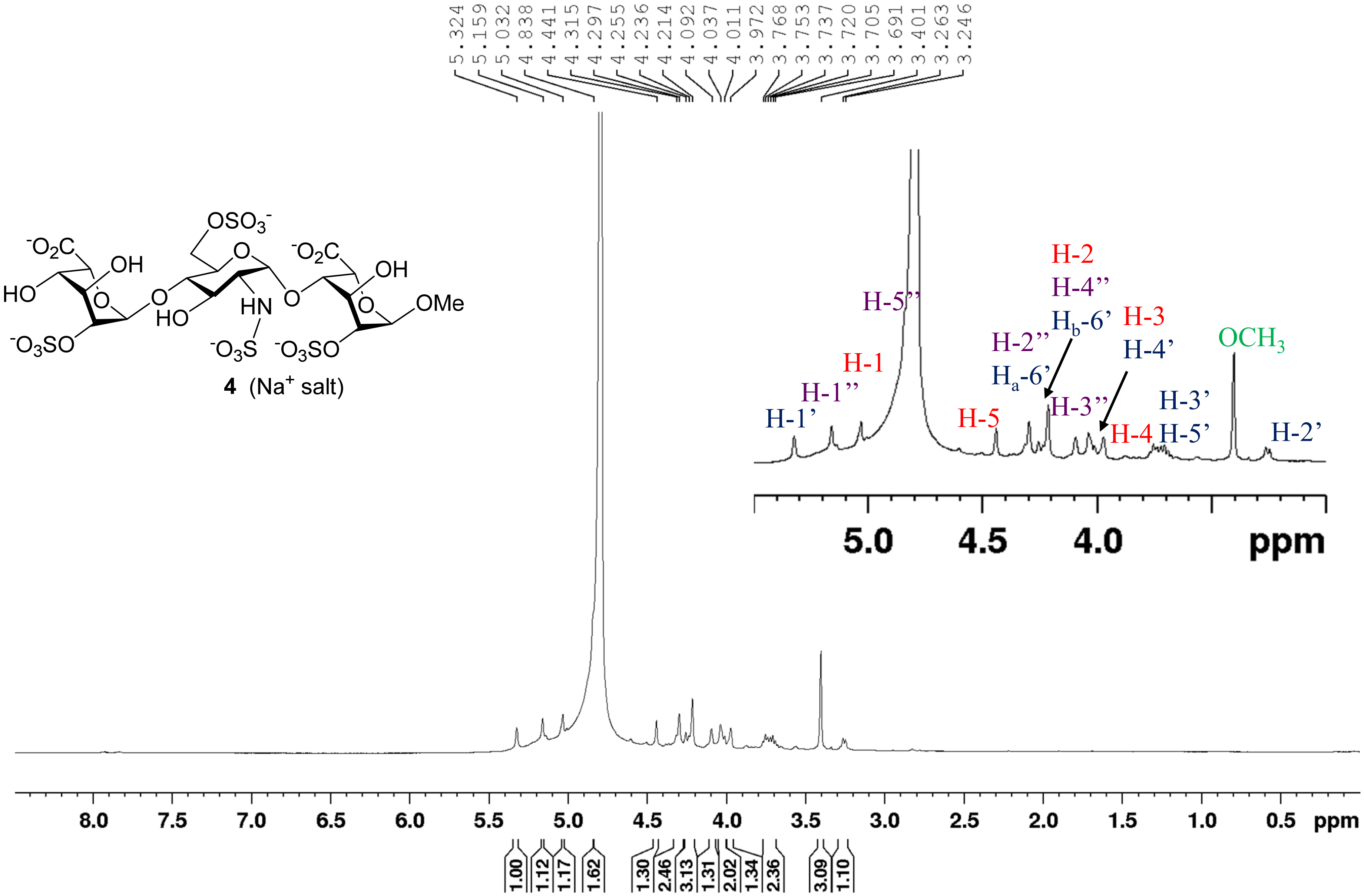
5.324
5.159
5.032
4.838
4.441
4.315
4.297
4.255
4.236
4.214
4.092
4.037
4.011
3.972
3.768
3.753
3.737
3.720
3.705
3.691
3.401
3.263
3.246
$^{-}O_2C$
OH
HO
O
$^{-}O_3SO$
OSO_3^{-}
O
HO
HN
$^{-}O_3S$
O
$^{-}O_2C$
OH
OMe
$^{-}O_3SO$
4 (Na+ salt)
H-1'
H-1''
H-1
H-5''
H-5
H-2''
Ha-6'
H-2
H-4''
Hb-6'
H-3''
H-3
H-4'
H-4
H-3'
H-5'
OCH3
H-2'
5.0
4.5
4.0
ppm
8.0
7.5
7.0
6.5
6.0
5.5
5.0
4.5
4.0
3.5
3.0
2.5
2.0
1.5
1.0
0.5
ppm
1.00
1.12
1.17
1.62
1.30
2.46
3.13
1.31
2.02
1.34
2.36
3.09
1.10

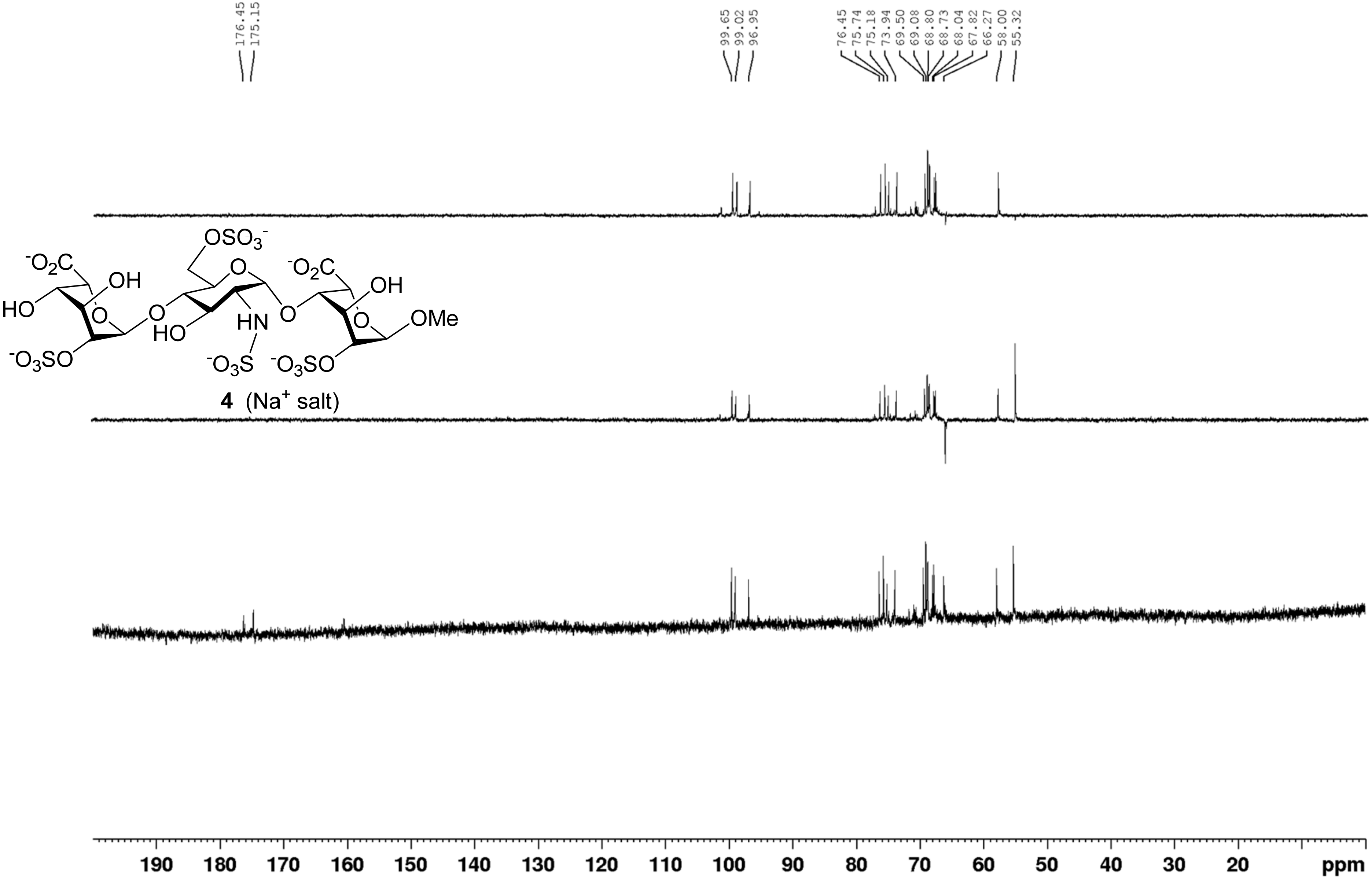
176.45
175.15
99.65
99.02
96.95
76.45
75.74
75.18
73.94
69.50
69.08
68.80
68.73
68.04
67.82
66.27
58.00
55.32
$^{-}O_2C$
OH
HO
O
$^{-}O_3SO$
OSO_3^{-}
HO
HN
$^{-}O_3S$
OMe
4 (Na+ salt)
190 180 170 160 150 140 130 120 110 100 90 80 70 60 50 40 30 20 ppm

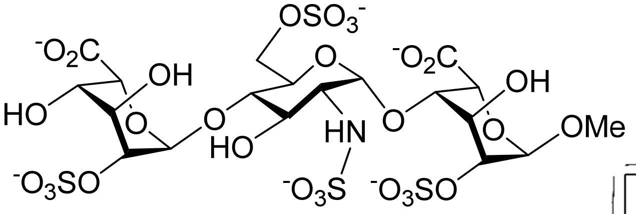

OSO3-
-O2C
OH
HO
O
HO
HN
O
-O2C
OH
OMe
-O3SO
-O3S
-O3SO

4 (Na+ salt)

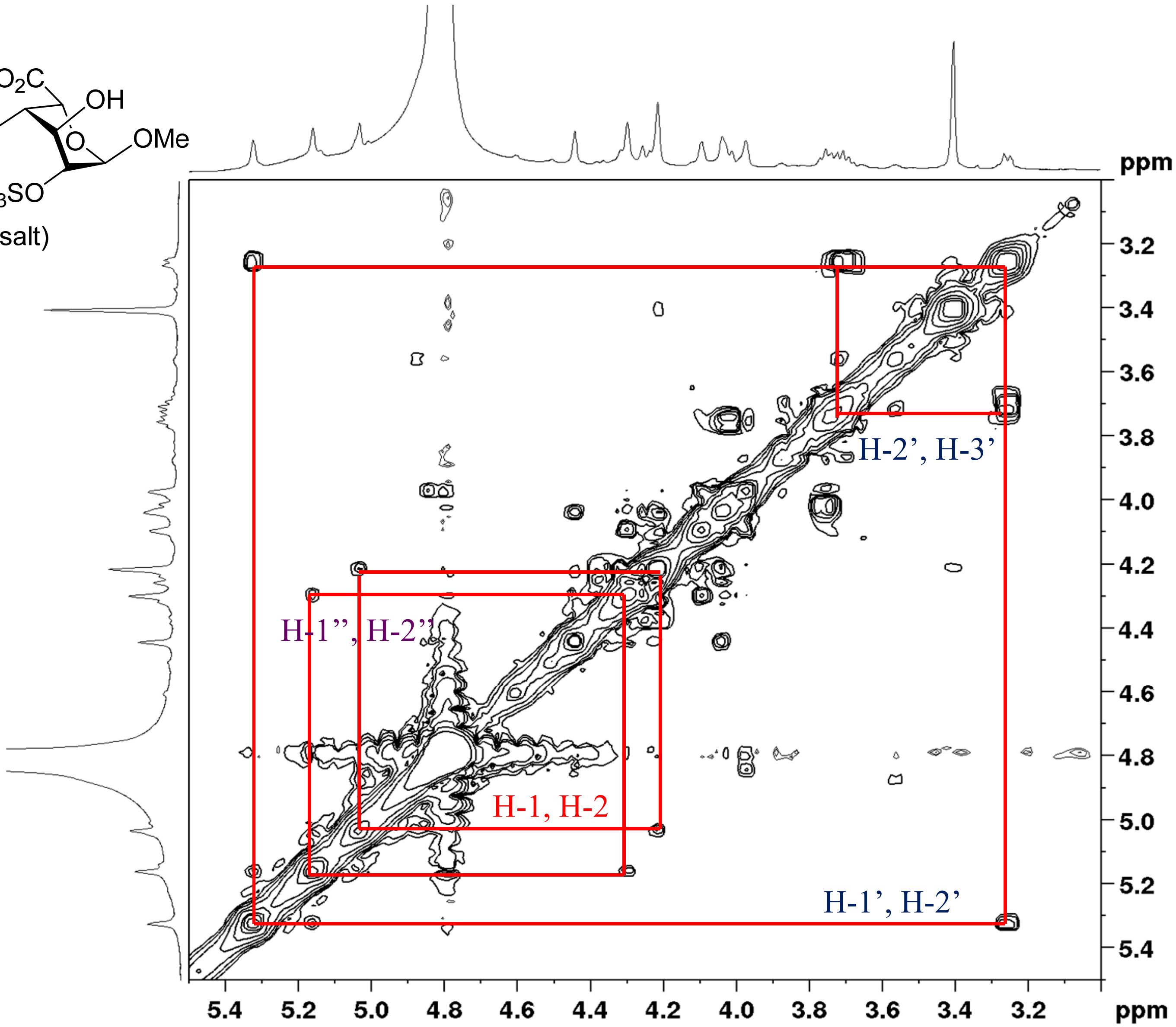

ppm
H-2', H-3'
H-1'', H-2''
H-1, H-2
H-1', H-2'
5.4
5.2
5.0
4.8
4.6
4.4
4.2
4.0
3.8
3.6
3.4
3.2
ppm

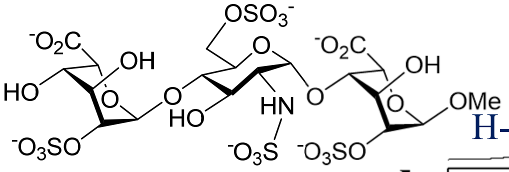

4 (Na+ salt)

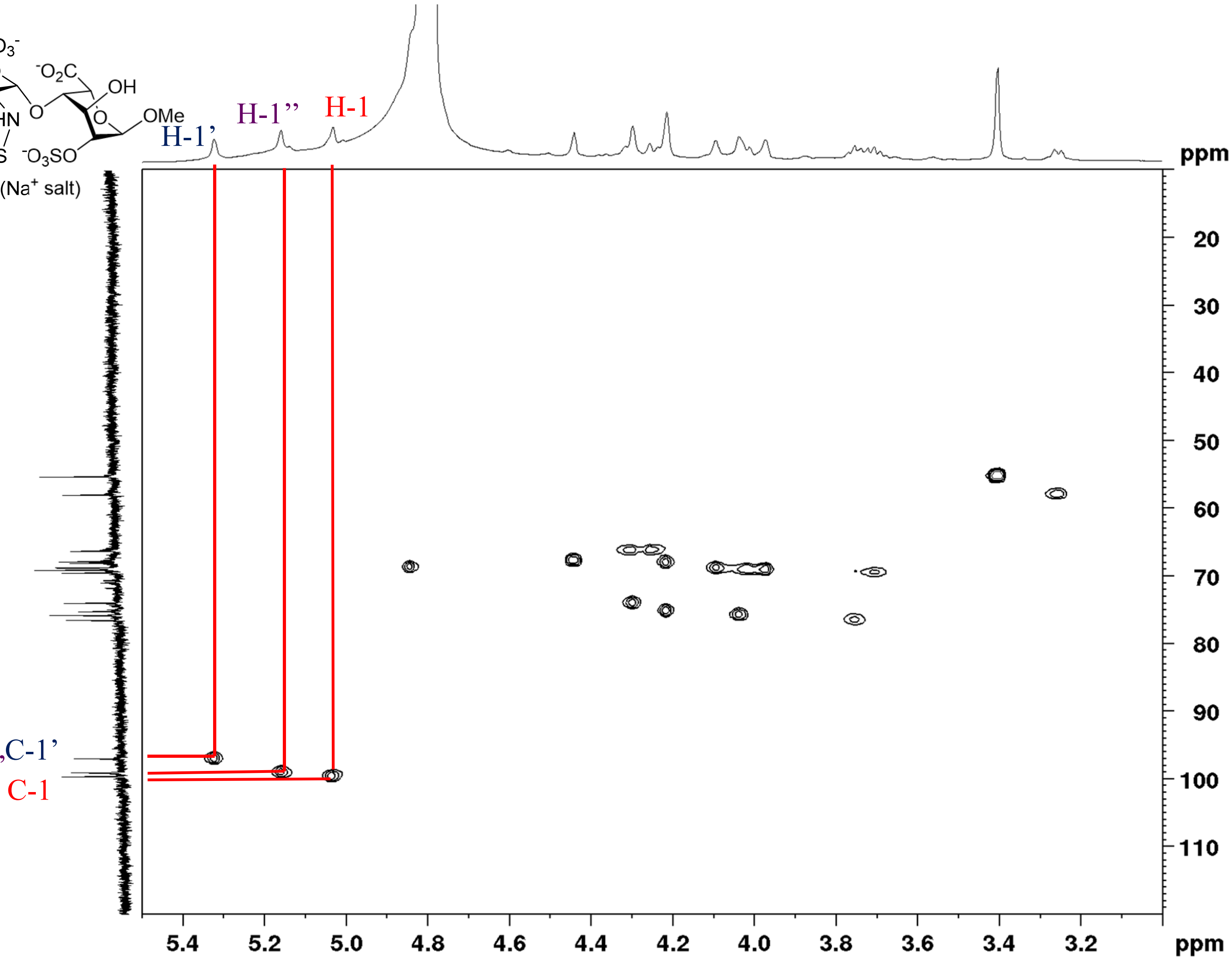
H-1'
H-1''
H-1
C-1'
C-1''
C-1
ppm
20
30
40
50
60
70
80
90
100
110
5.4
5.2
5.0
4.8
4.6
4.4
4.2
4.0
3.8
3.6
3.4
3.2
ppm

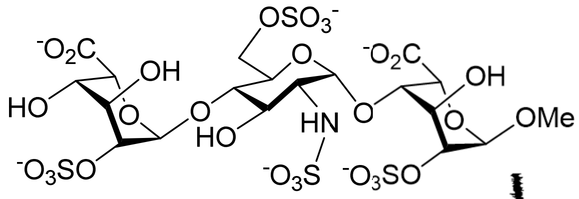

4 (Na$^+$ salt)

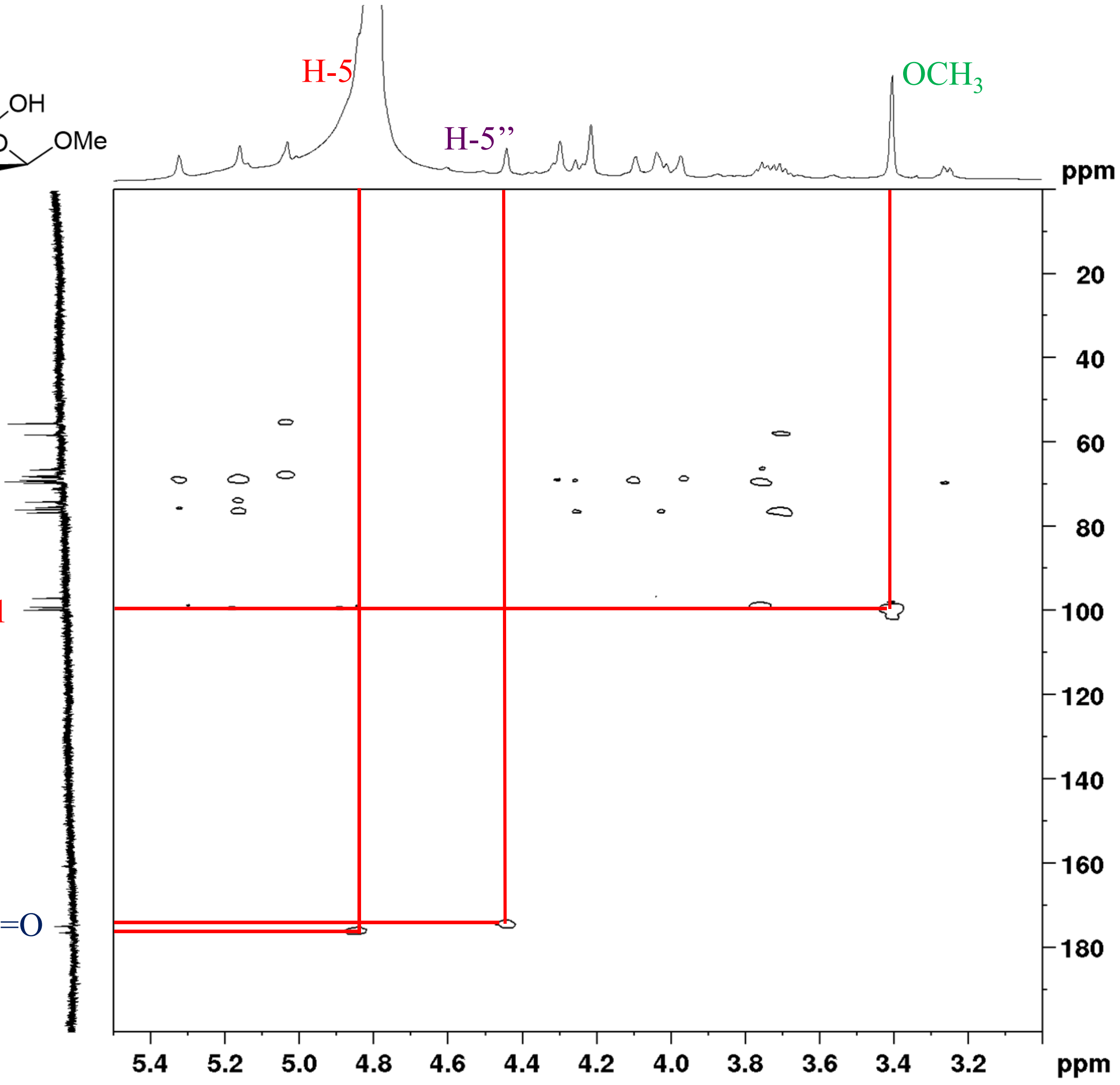
H-5
H-5''
OCH$_3$
ppm
C-1
C=O
20
40
60
80
100
120
140
160
180
5.4
5.2
5.0
4.8
4.6
4.4
4.2
4.0
3.8
3.6
3.4
3.2
ppm

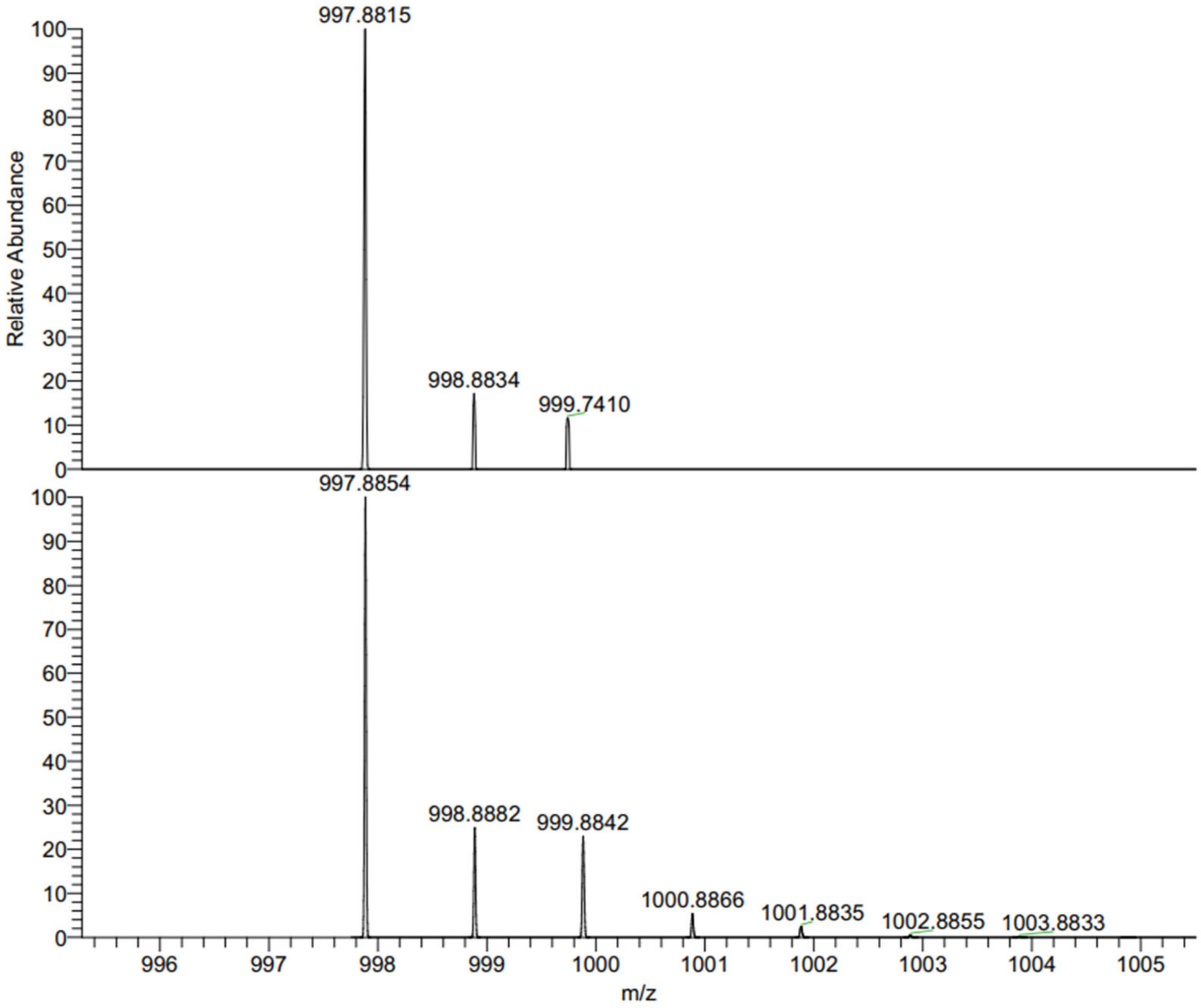

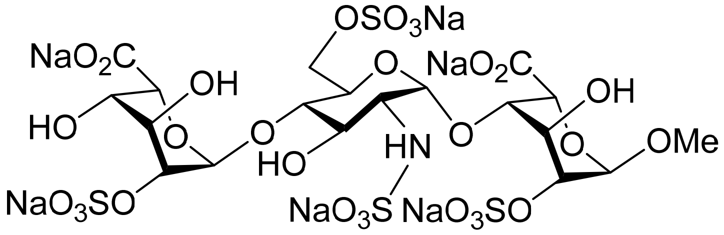

4 (Na^+ salt)

Chemical Formula: $C_{19}H_{25}NNa_6O_{29}S_4$
Exact Mass: 996.8781
Molecular Weight: 997.5656

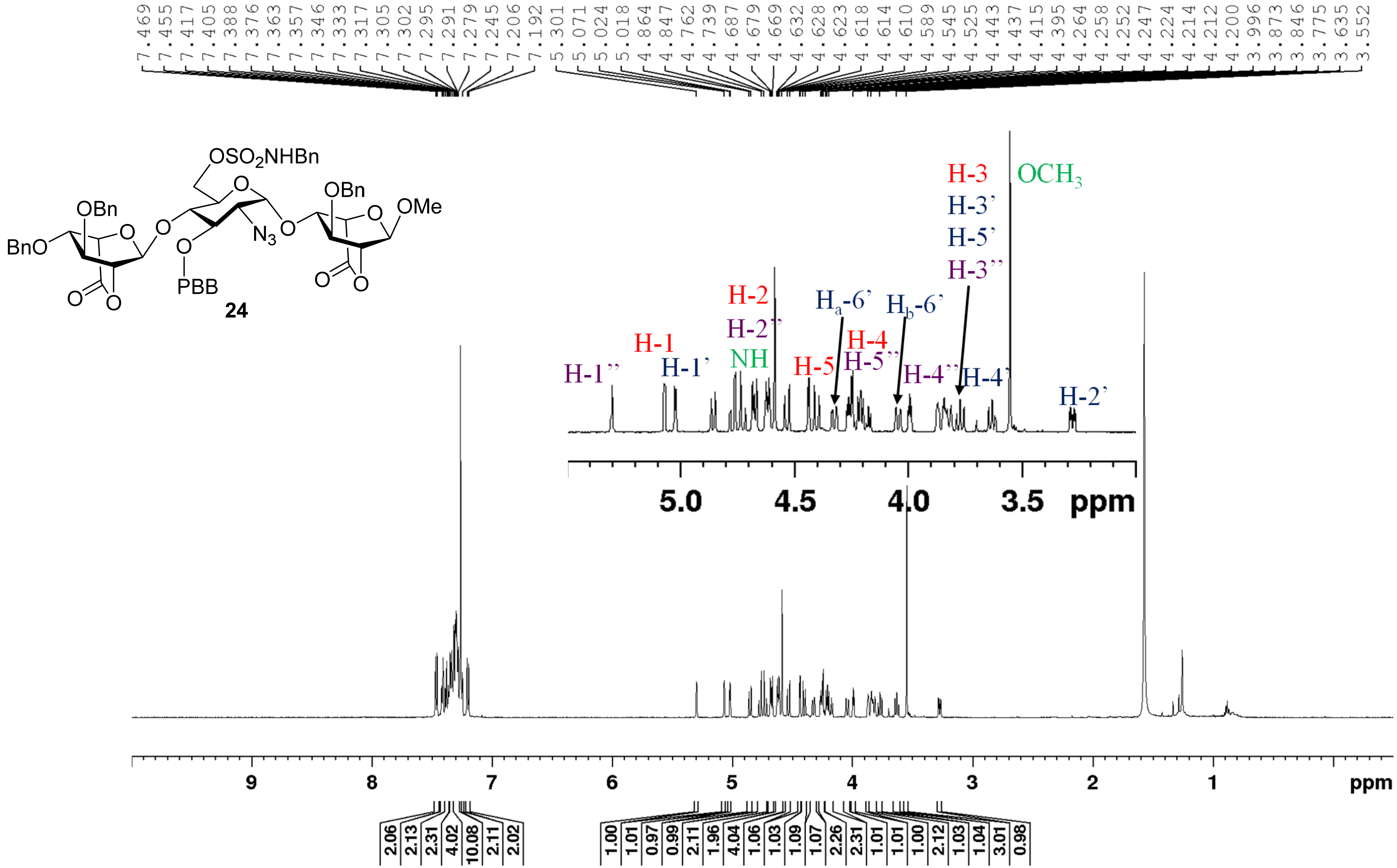

OSO2NHBn
OBn
OBn
OMe
BnO
O
N3
PBB
24
H-1''
H-1
H-1'
H-2
H-2''
NH
H-5
Ha-6'
H-4
H-5''
Hb-6'
H-4''
H-4'
H-3
H-3'
H-5'
H-3''
OCH3
H-2'
5.0
4.5
4.0
3.5
ppm
9
8
7
6
5
4
3
2
1
ppm

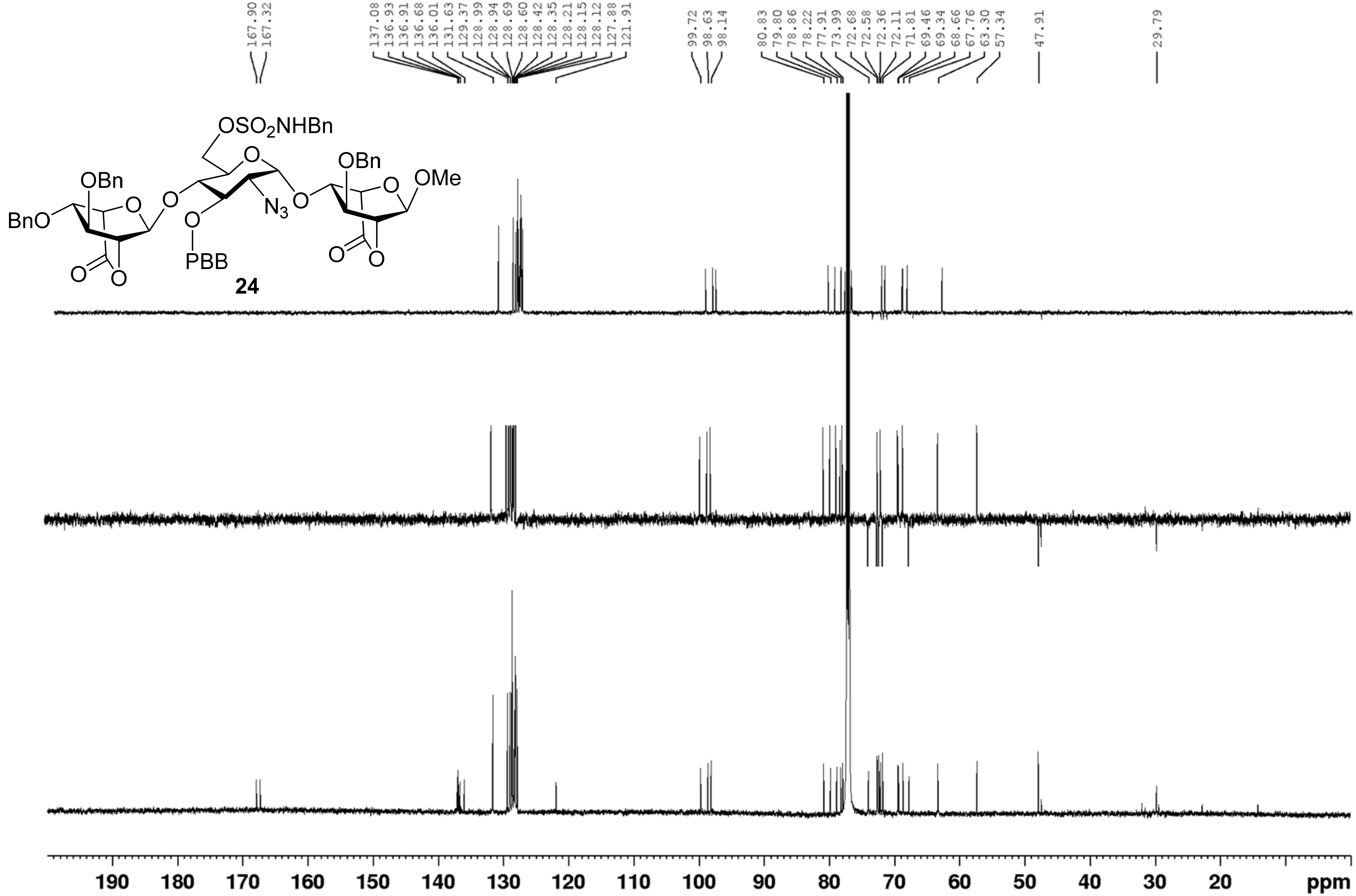
167.90
167.32
137.08
136.93
136.91
136.68
136.01
131.63
129.37
128.99
128.94
128.69
128.60
128.42
128.35
128.21
128.15
128.12
127.88
121.91
99.72
98.63
98.14
80.83
79.80
78.86
78.22
77.91
73.99
72.68
72.58
72.36
72.11
71.81
69.46
69.34
68.66
67.76
63.30
57.34
47.91
29.79
OSO2NHBn
OBn
OBn
OMe
BnO
N3
PBB
24
190 180 170 160 150 140 130 120 110 100 90 80 70 60 50 40 30 20 ppm

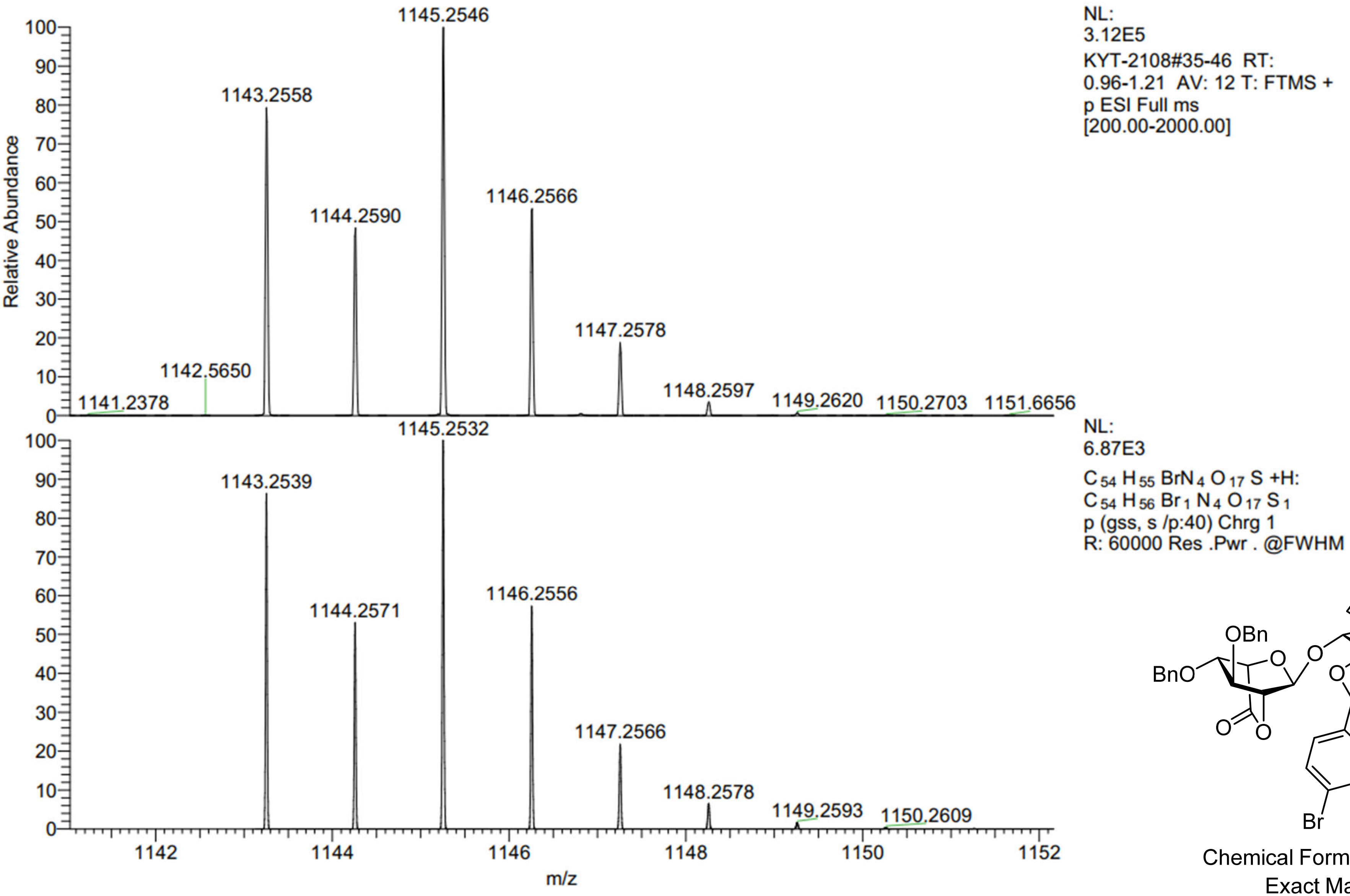

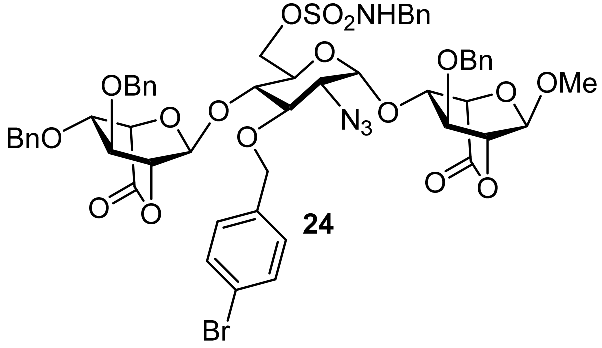

24

Chemical Formula: $C_{54}H_{55}BrN_4O_{17}S$
Exact Mass: 1142.2466
Molecular Weight: 1144.0090

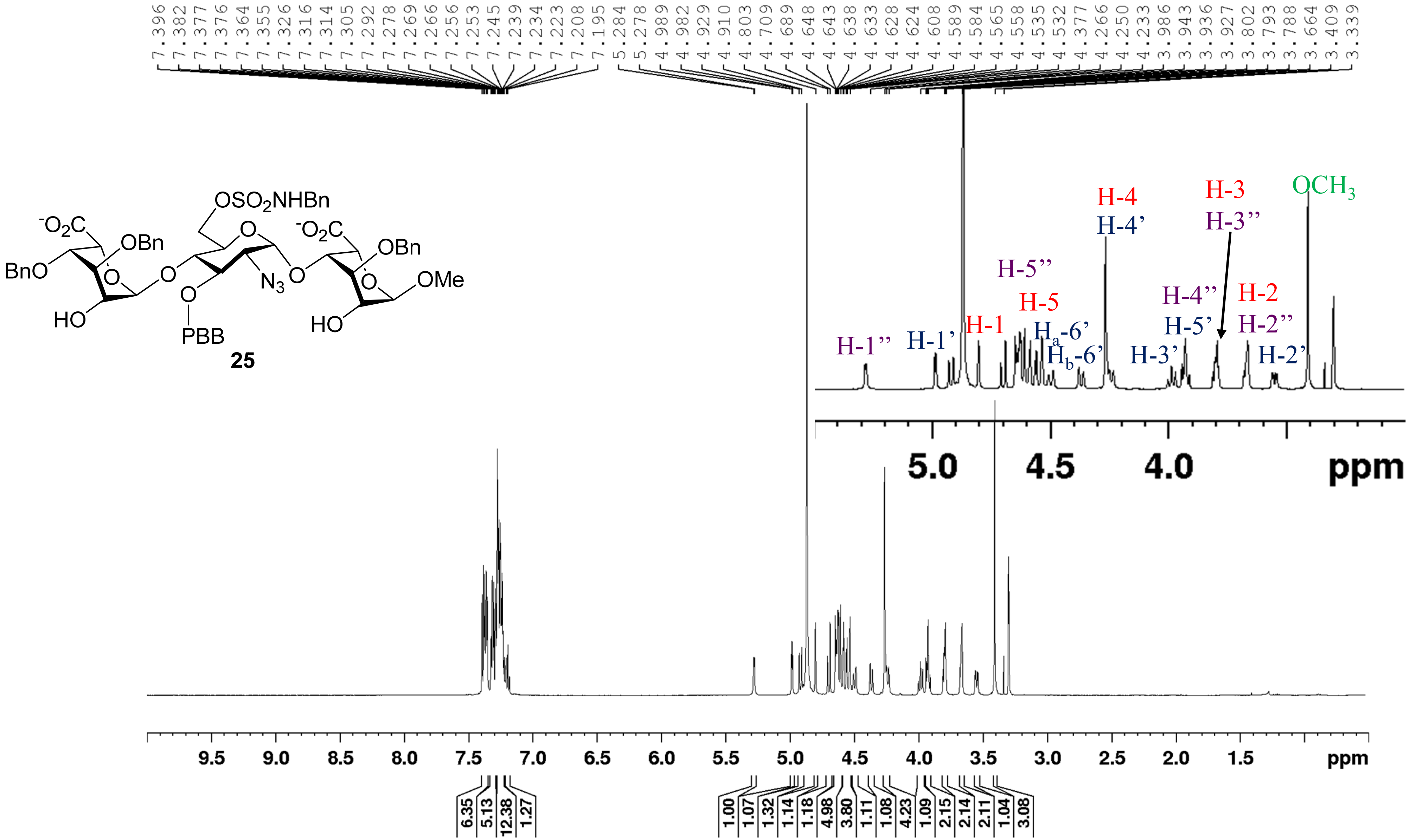
OSO2NHBn
-O2C
OBn
BnO
HO
PBB
N3
OMe
25
H-1''
H-1'
H-1
H-5''
H-5
Ha-6'
Hb-6'
H-4
H-4'
H-3'
H-4''
H-5'
H-3
H-3''
H-2
H-2''
H-2'
OCH3
ppm

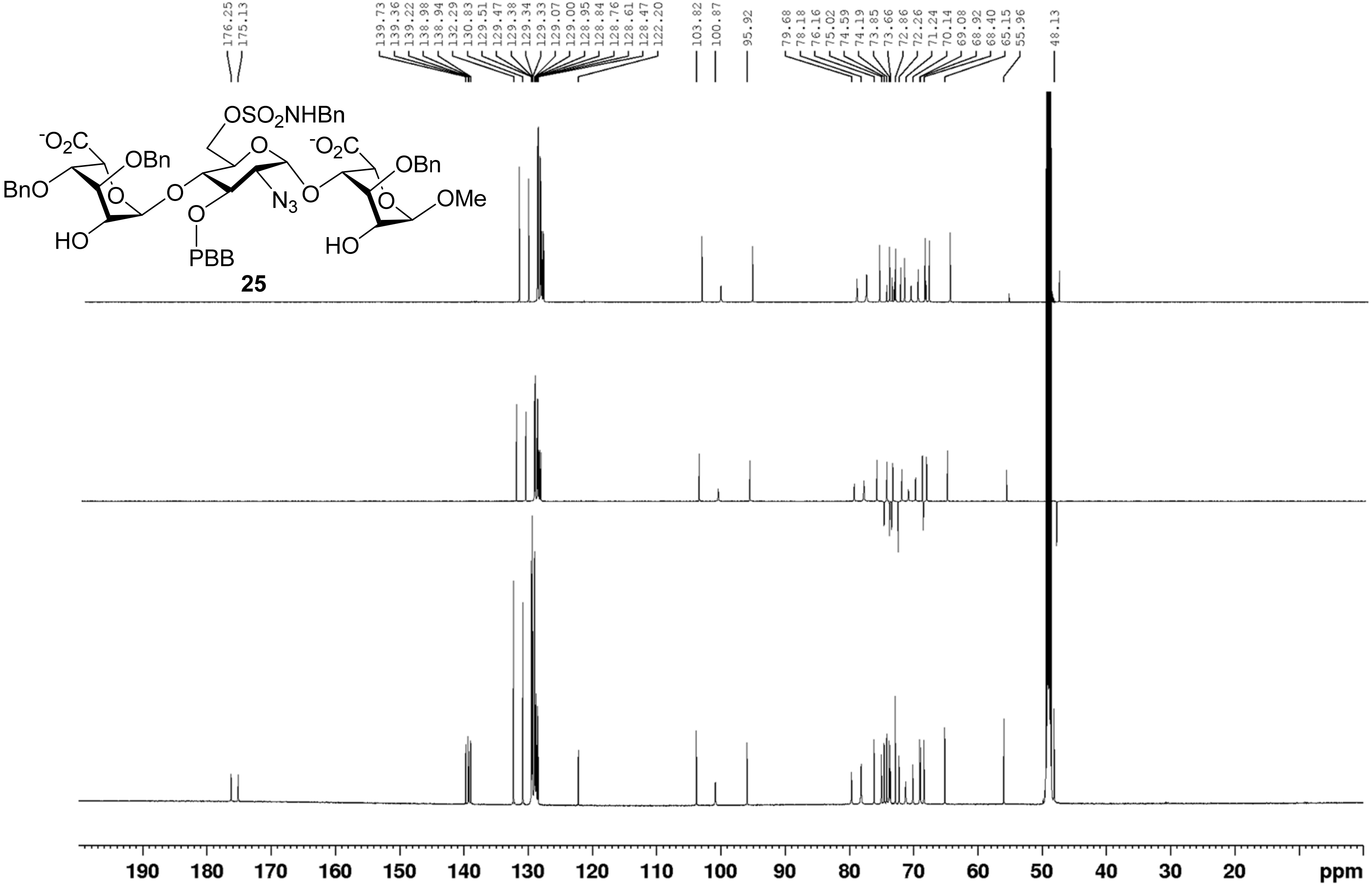

176.25
175.13
139.73
139.36
139.22
138.98
138.94
132.29
130.83
129.51
129.47
129.38
129.34
129.33
129.07
129.00
128.95
128.84
128.76
128.61
128.47
122.20
103.82
100.87
95.92
79.68
78.18
76.16
75.02
74.59
74.19
73.85
73.66
72.86
72.26
71.24
70.14
69.08
68.92
68.40
65.15
55.96
48.13
OSO2NHBn
-O2C
OBn
BnO
O
N3
OMe
HO
PBB
25
190 180 170 160 150 140 130 120 110 100 90 80 70 60 50 40 30 20 ppm

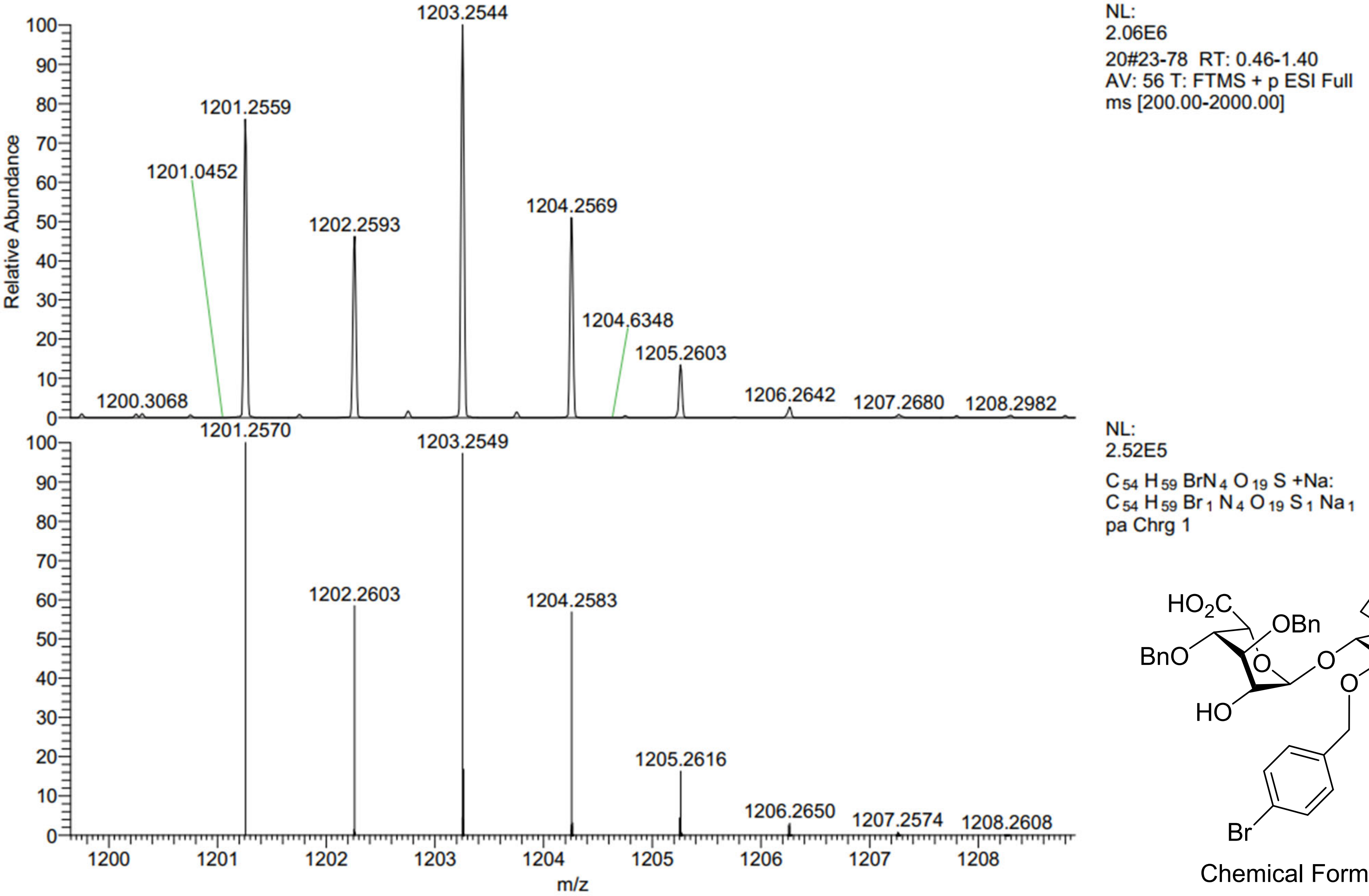

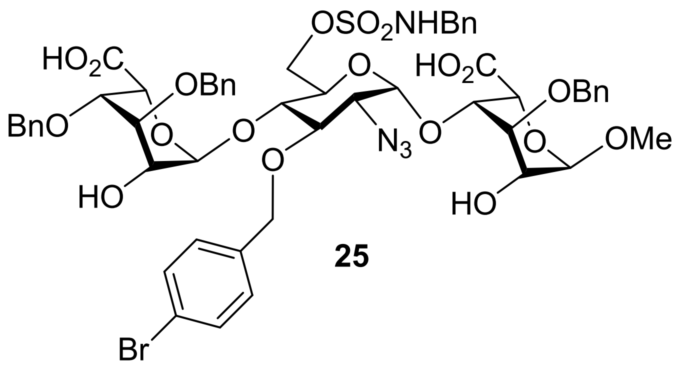

25

Chemical Formula: $C_{54}H_{59}BrN_4O_{19}S$
Exact Mass: 1178.2678
Molecular Weight: 1180.0390

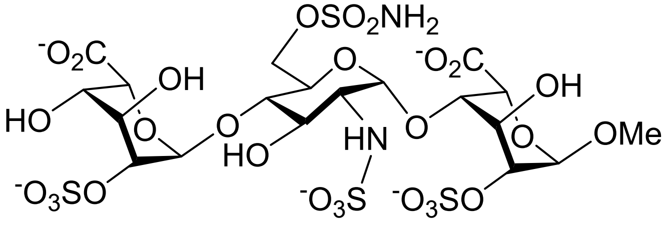

5 (Na$^+$ salt)

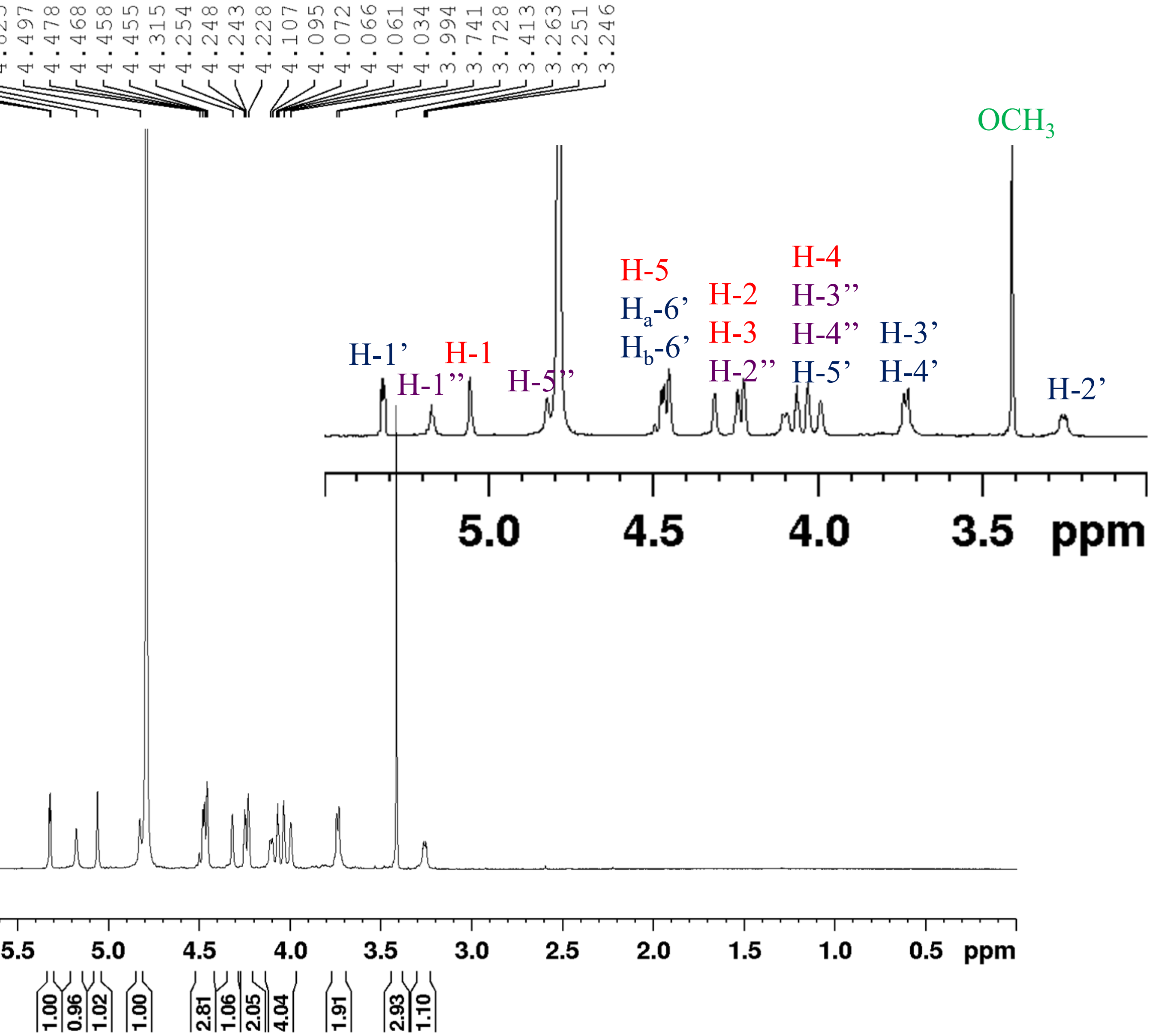

5.323
5.318
5.175
5.059
4.825
4.497
4.478
4.468
4.458
4.455
4.315
4.254
4.248
4.243
4.228
4.107
4.095
4.072
4.066
4.061
4.034
3.994
3.741
3.728
3.413
3.263
3.251
3.246
OCH3
H-1'
H-1''
H-1
H-5''
H-5
Ha-6'
Hb-6'
H-2
H-3
H-2''
H-4
H-3''
H-4''
H-5'
H-3'
H-4'
H-2'
ppm
1.00
0.96
1.02
1.00
2.81
1.06
2.05
4.04
1.91
2.93
1.10

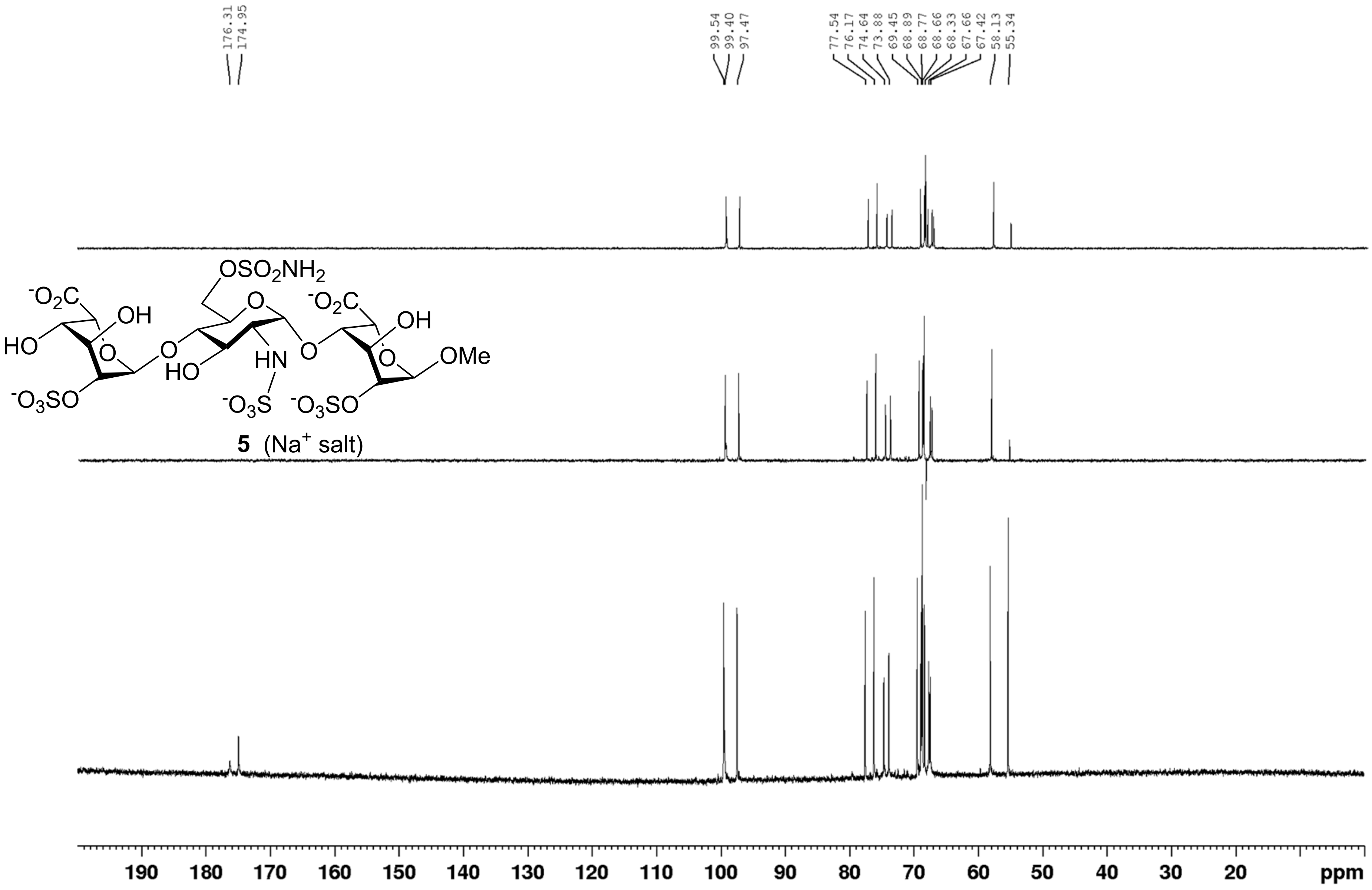
176.31
174.95
99.54
99.40
97.47
77.54
76.17
74.64
73.88
69.45
68.89
68.77
68.66
68.33
67.66
67.42
58.13
55.34
5 (Na+ salt)
190 180 170 160 150 140 130 120 110 100 90 80 70 60 50 40 30 20 ppm

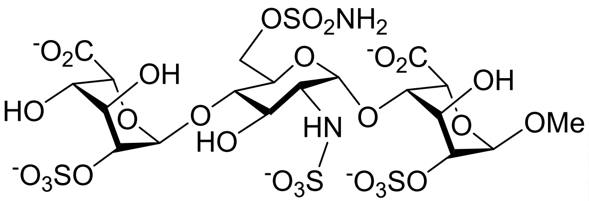

5 (Na$^+$ salt)

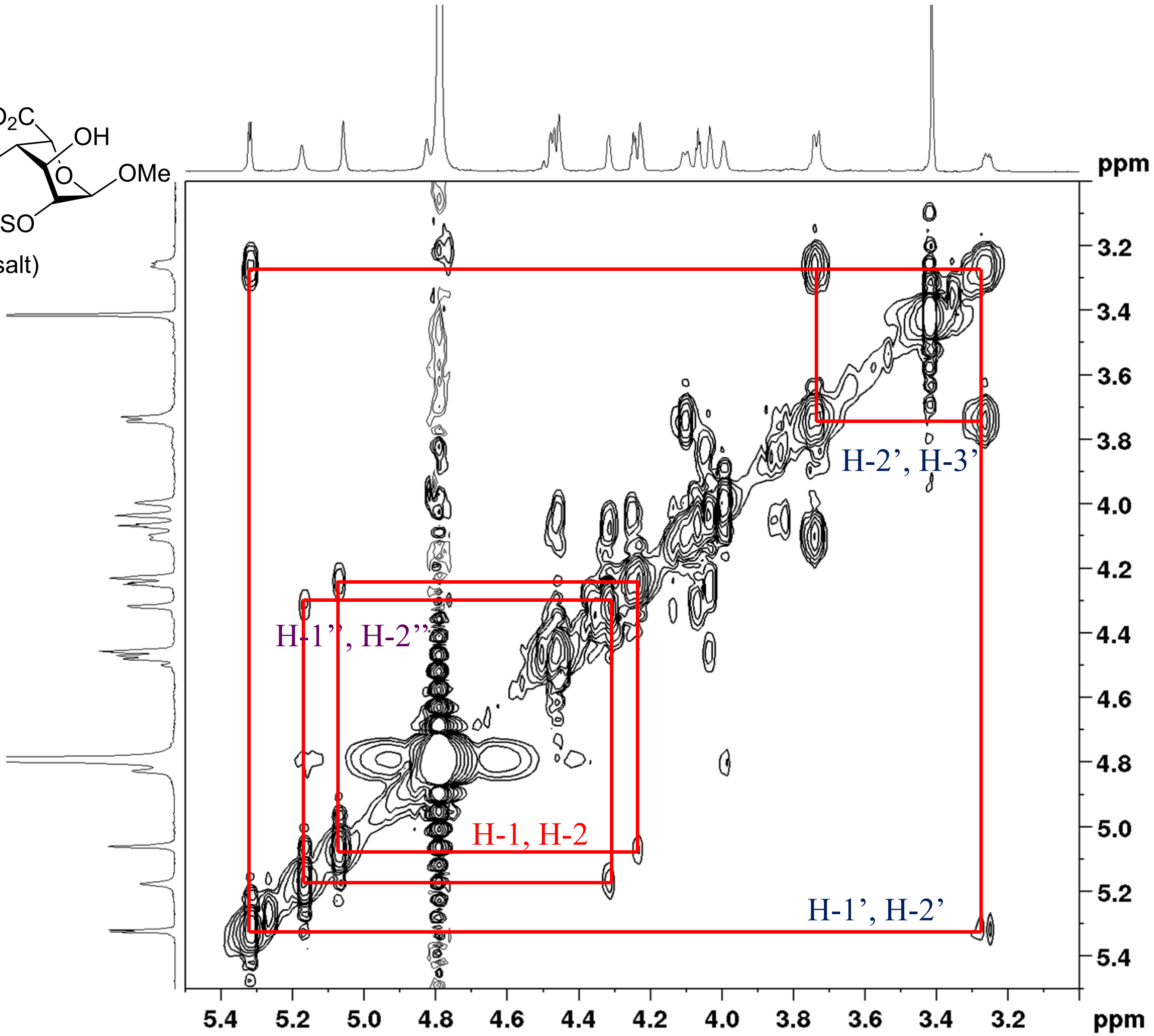
ppm
H-2', H-3'
H-1'', H-2''
H-1, H-2
H-1', H-2'
3.2
3.4
3.6
3.8
4.0
4.2
4.4
4.6
4.8
5.0
5.2
5.4
5.4 5.2 5.0 4.8 4.6 4.4 4.2 4.0 3.8 3.6 3.4 3.2
ppm

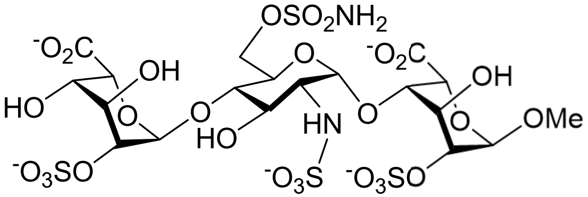

5 (Na$^+$ salt)

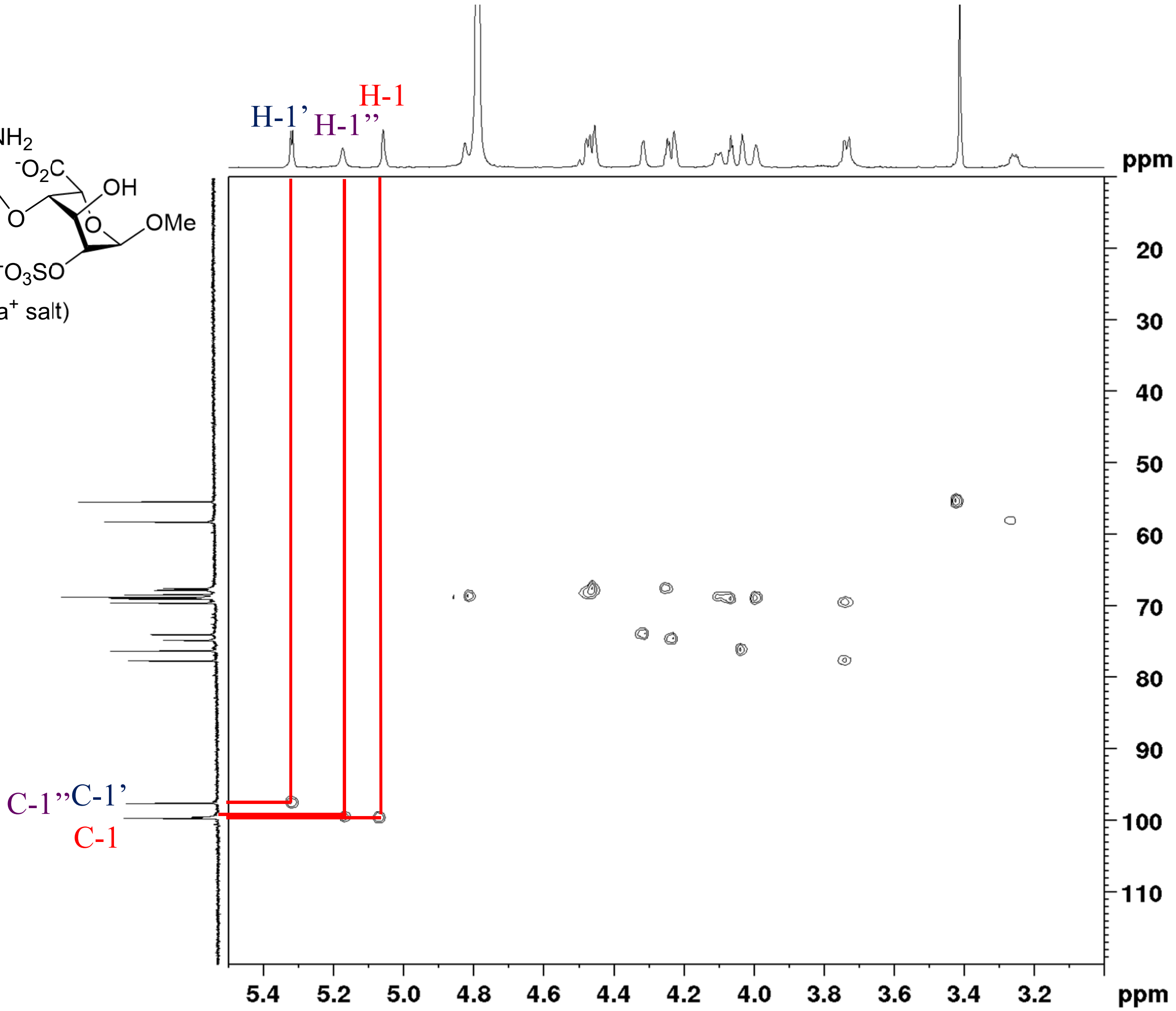
H-1'
H-1''
H-1
ppm
20
30
40
50
60
70
80
90
100
110
C-1''
C-1'
C-1
5.4
5.2
5.0
4.8
4.6
4.4
4.2
4.0
3.8
3.6
3.4
3.2
ppm

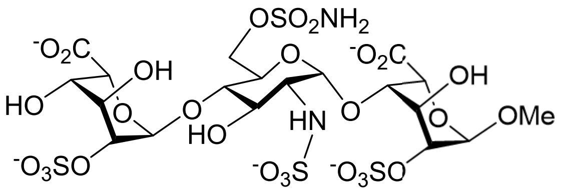
OSO2NH2
-O2C
OH
HO
O
-O3SO
HO
HN
-O3S
-O3SO
OMe

5 (Na+ salt)

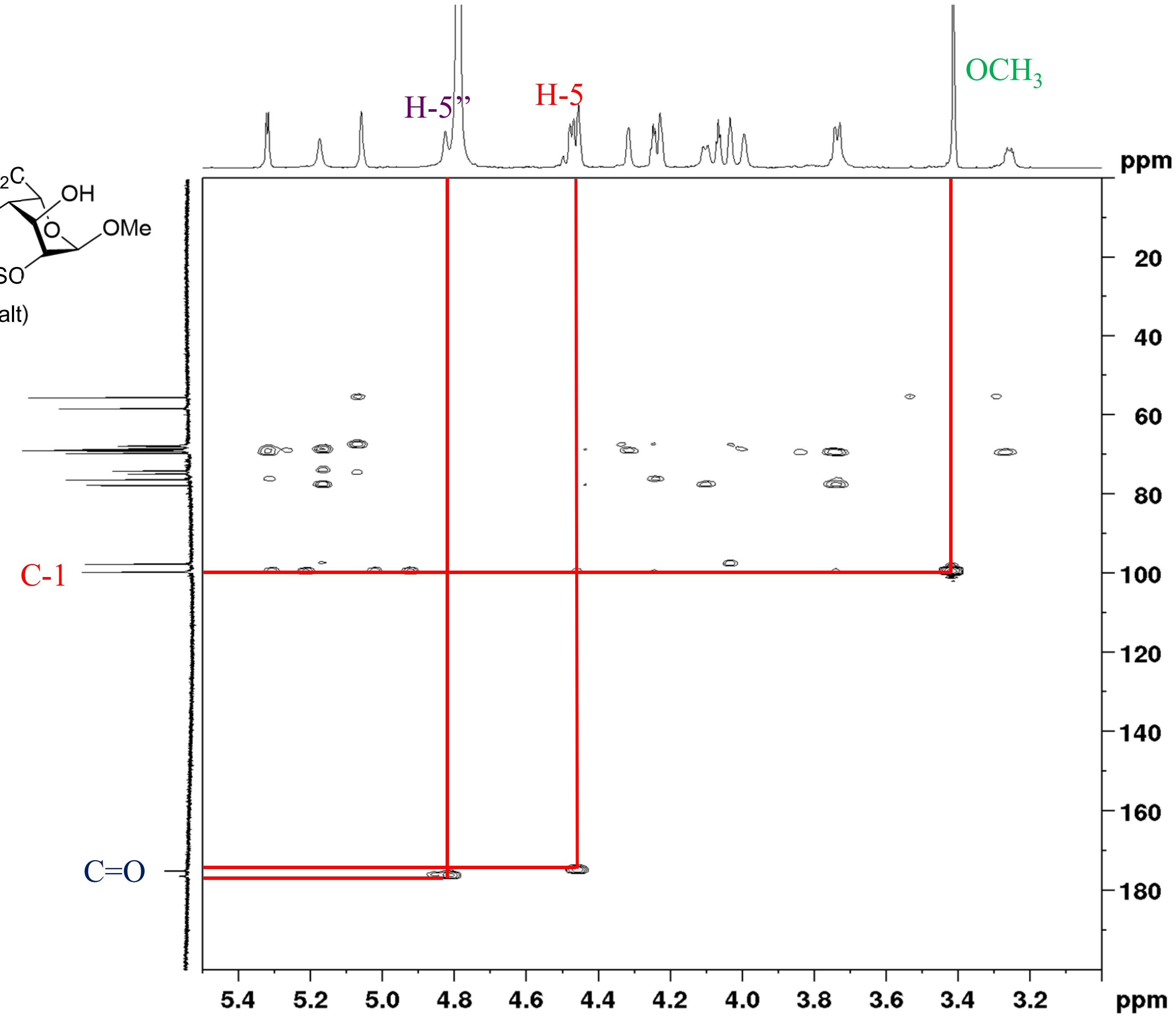
H-5"
H-5
OCH3
ppm
C-1
C=O
20
40
60
80
100
120
140
160
180
5.4
5.2
5.0
4.8
4.6
4.4
4.2
4.0
3.8
3.6
3.4
3.2
ppm

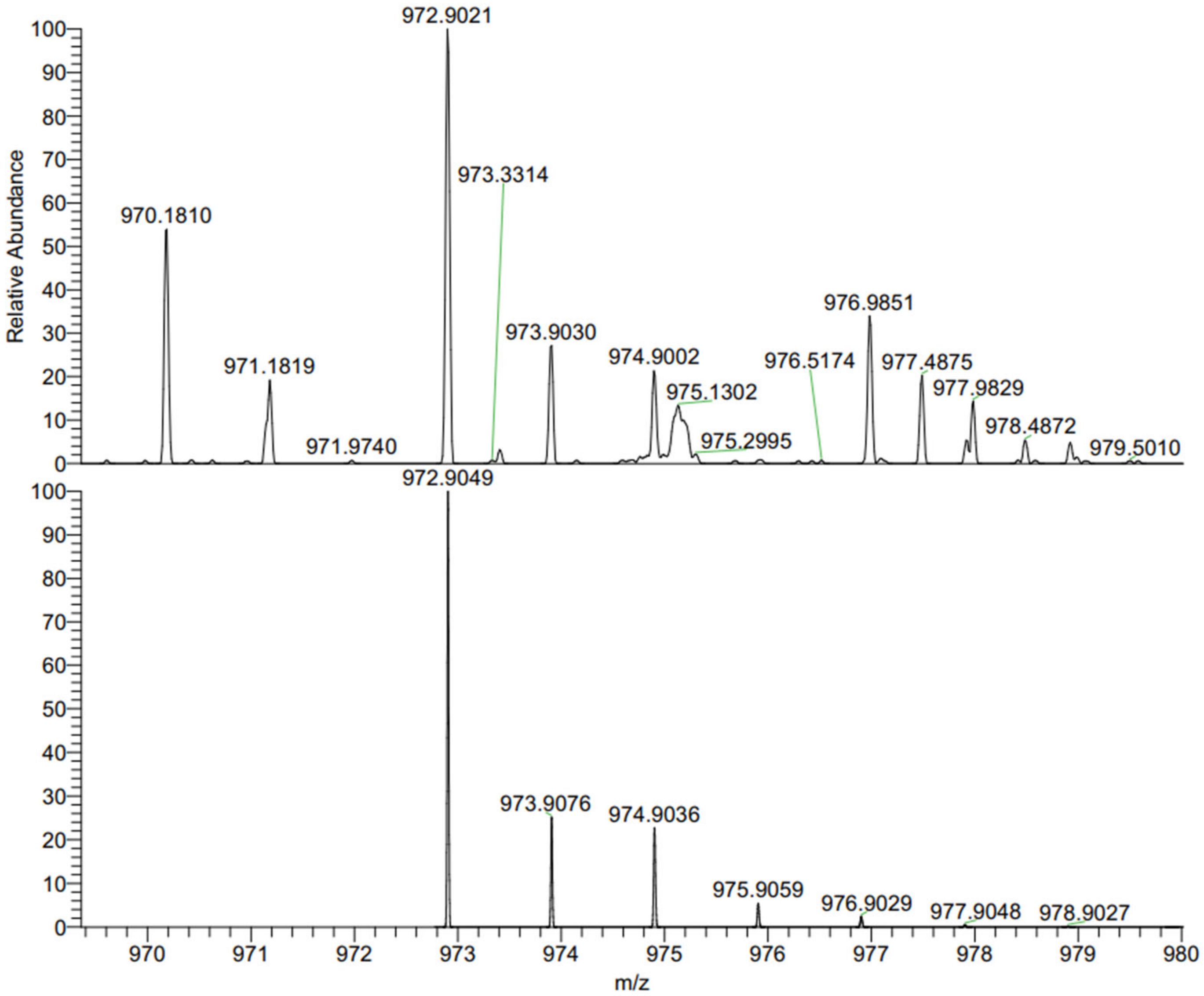

NL:
1.61E3
KYT-3020#20-42 RT:
0.51-1.10 AV: 23 T: FTMS - p
ESI Full ms [200.00-2000.00]

NL:
1.44E4
C 19 H 27 N 2 Na 5 O 28 S 4 +H:
C 19 H 26 N 2 Na 5 O 28 S 4
p (gss, s /p:40) Chrg -1
R: 60000 Res .Pwr . @FWHM

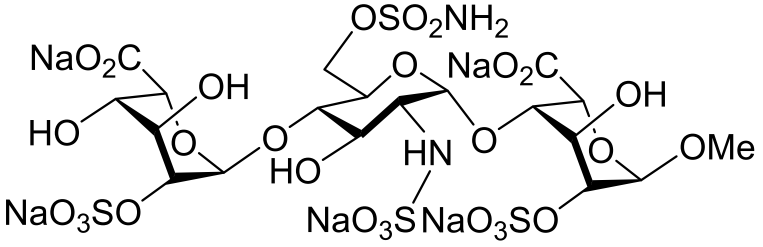

5 (Na$^+$ salt)

Chemical Formula: $C_{19}H_{27}N_2Na_5O_{28}S_4$
Exact Mass: 973.9122
Molecular Weight: 974.5998

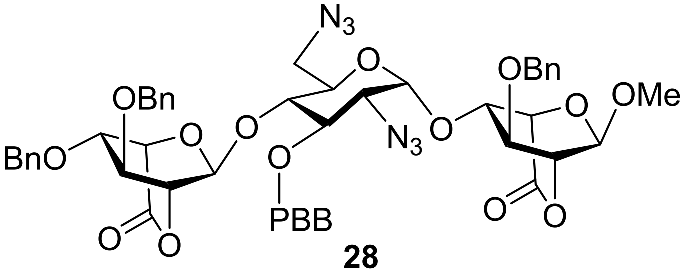
N3
OBn
OBn
OMe
BnO
O
N3
PBB

28

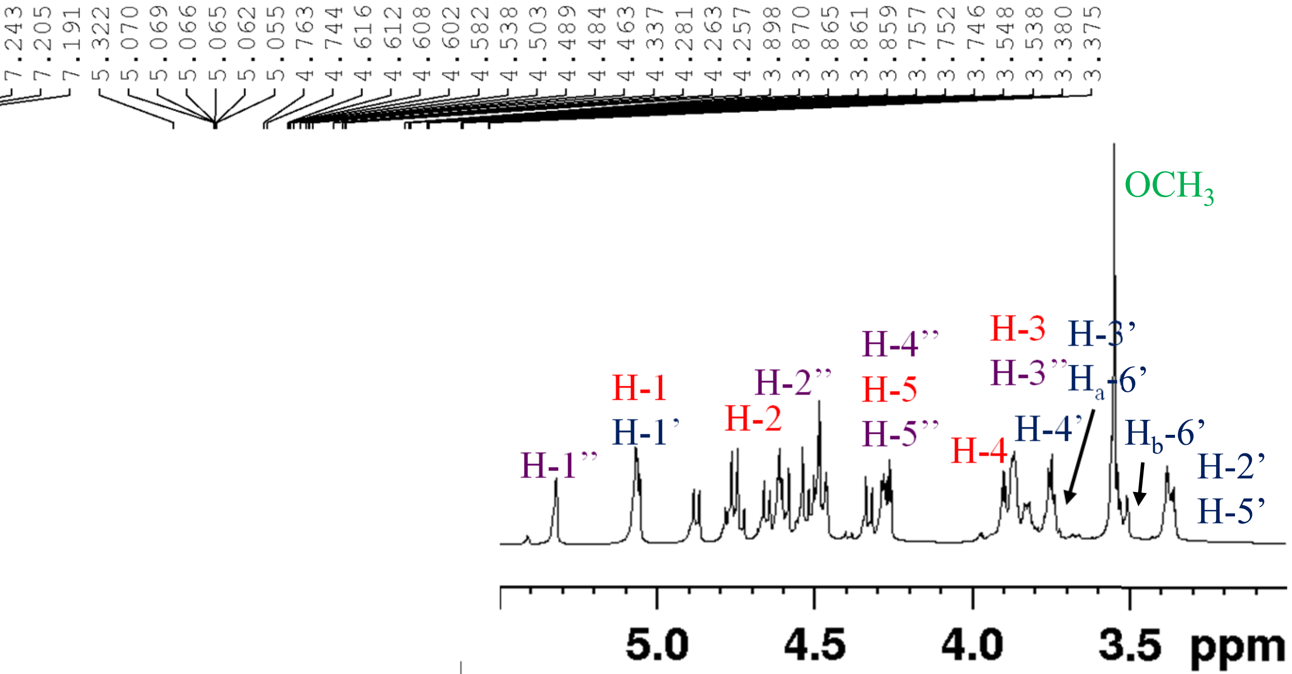
7.464 7.450 7.417 7.406 7.385 7.383 7.374 7.363 7.351 7.336 7.326 7.313 7.305 7.304 7.297 7.294 7.292 7.285 7.266 7.255 7.243 7.205 7.191 5.322 5.070 5.069 5.066 5.065 5.062 5.055 4.763 4.744 4.616 4.612 4.608 4.602 4.582 4.538 4.503 4.489 4.484 4.463 4.337 4.281 4.263 4.257 3.898 3.870 3.865 3.861 3.859 3.757 3.752 3.746 3.548 3.538 3.380 3.375
OCH3
H-1''
H-1
H-1'
H-2
H-2''
H-4''
H-5
H-5''
H-4
H-3
H-3''
H-4'
H-3'
Ha-6'
Hb-6'
H-2'
H-5'
5.0
4.5
4.0
3.5 ppm

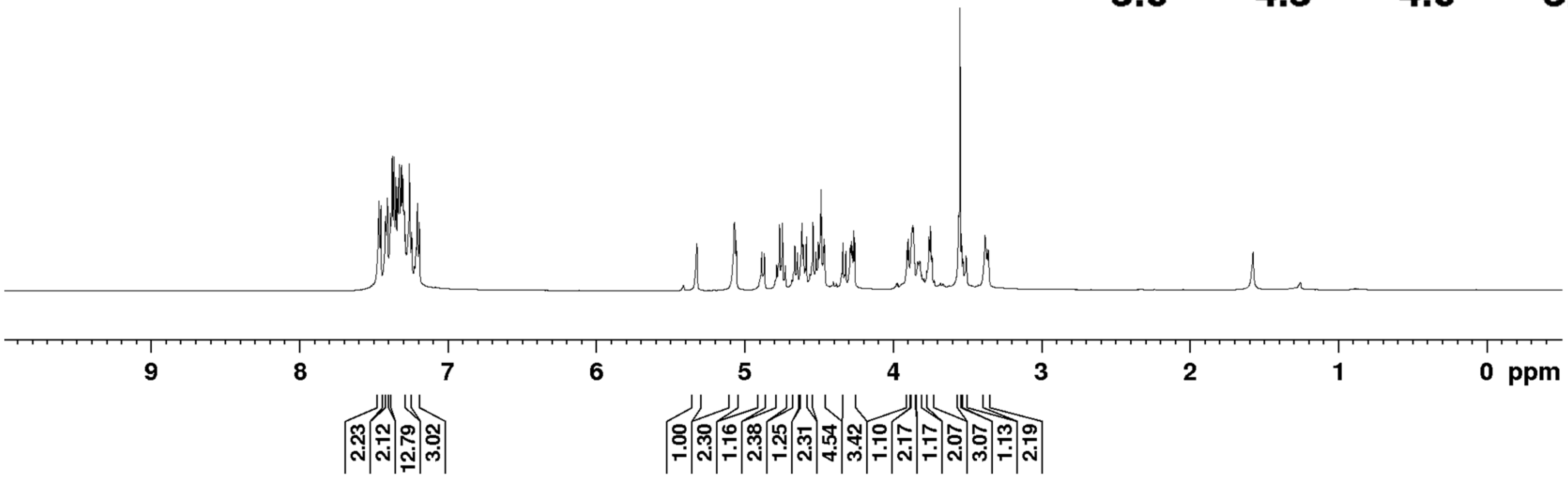
9
8
7
6
5
4
3
2
1
0 ppm
2.23 2.12 12.79 3.02
1.00 2.30 1.16 2.38 1.25 2.31 4.54 3.42 1.10 2.17 1.17 2.07 3.07 1.13 2.19

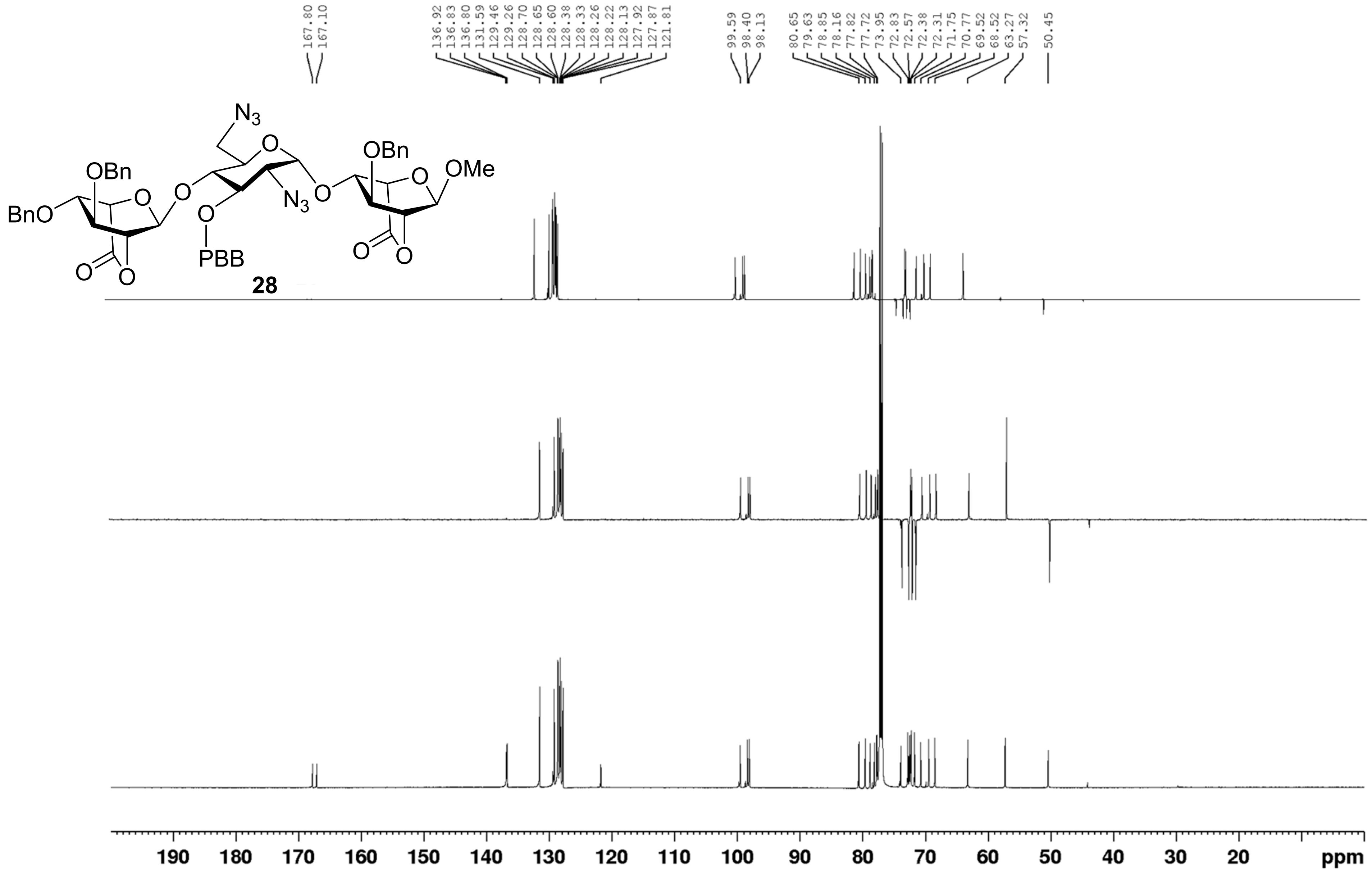
167.80
167.10
136.92
136.83
136.80
131.59
129.46
129.26
128.70
128.65
128.60
128.38
128.33
128.26
128.22
128.13
127.92
127.87
121.81
99.59
98.40
98.13
80.65
79.63
78.85
78.16
77.82
77.72
73.95
72.83
72.57
72.38
72.31
71.75
70.77
69.52
68.52
63.27
57.32
50.45
N3
O
OBn
OMe
BnO
N3
PBB
28
190 180 170 160 150 140 130 120 110 100 90 80 70 60 50 40 30 20 ppm

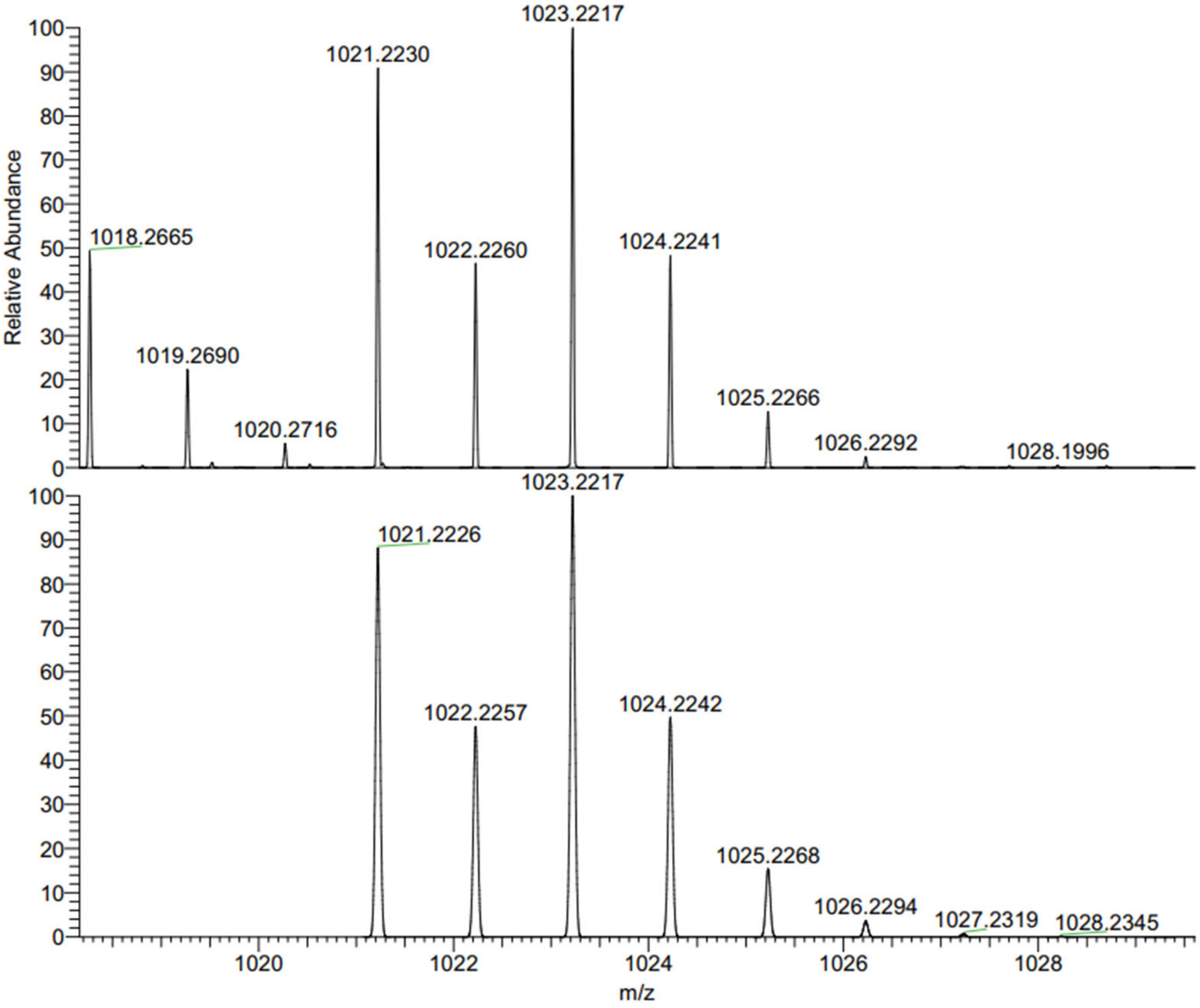

NL:
4.87E5
KYT-2050#26-35 RT:
0.73-0.88 AV: 10 T: FTMS +
p ESI Full ms
[200.00-2000.00]

NL:
7.65E3
C 47 H 47 BrN 6 O 14 +Na:
C 47 H 47 Br 1 N 6 O 14 Na 1
p (gss, s /p:40) Chrg 1
R: 20000 Res .Pwr . @FWHM

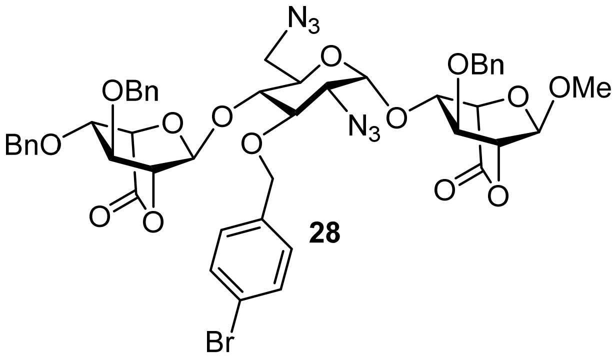

28

Chemical Formula: $C_{47}H_{47}BrN_6O_{14}$
Exact Mass: 998.2334
Molecular Weight: 999.8250

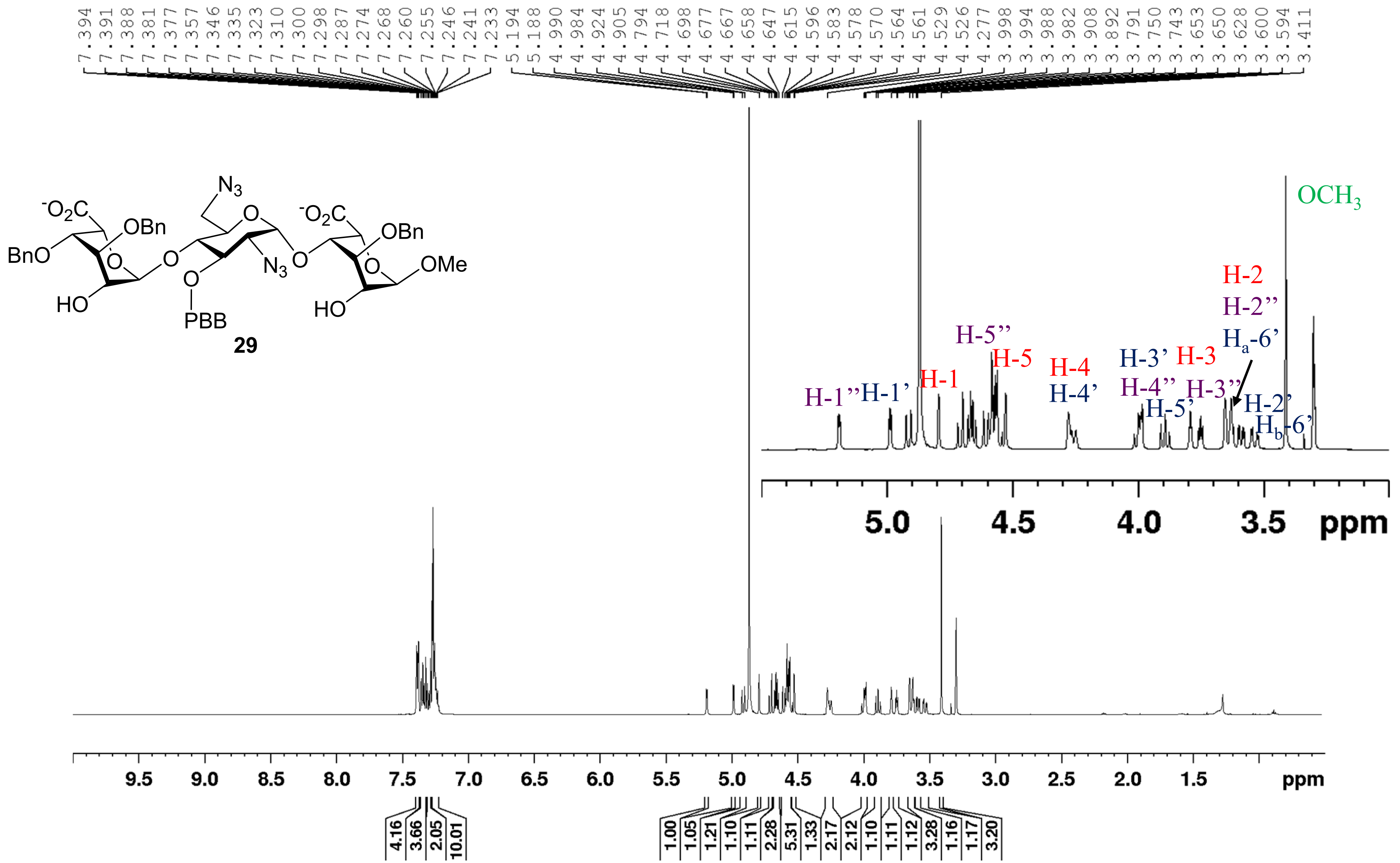
29
OCH3
H-1''
H-1'
H-1
H-5''
H-5
H-4
H-4'
H-3'
H-4''
H-5'
H-3
H-3''
H-2
H-2''
Ha-6'
H-2'
Hb-6'
ppm

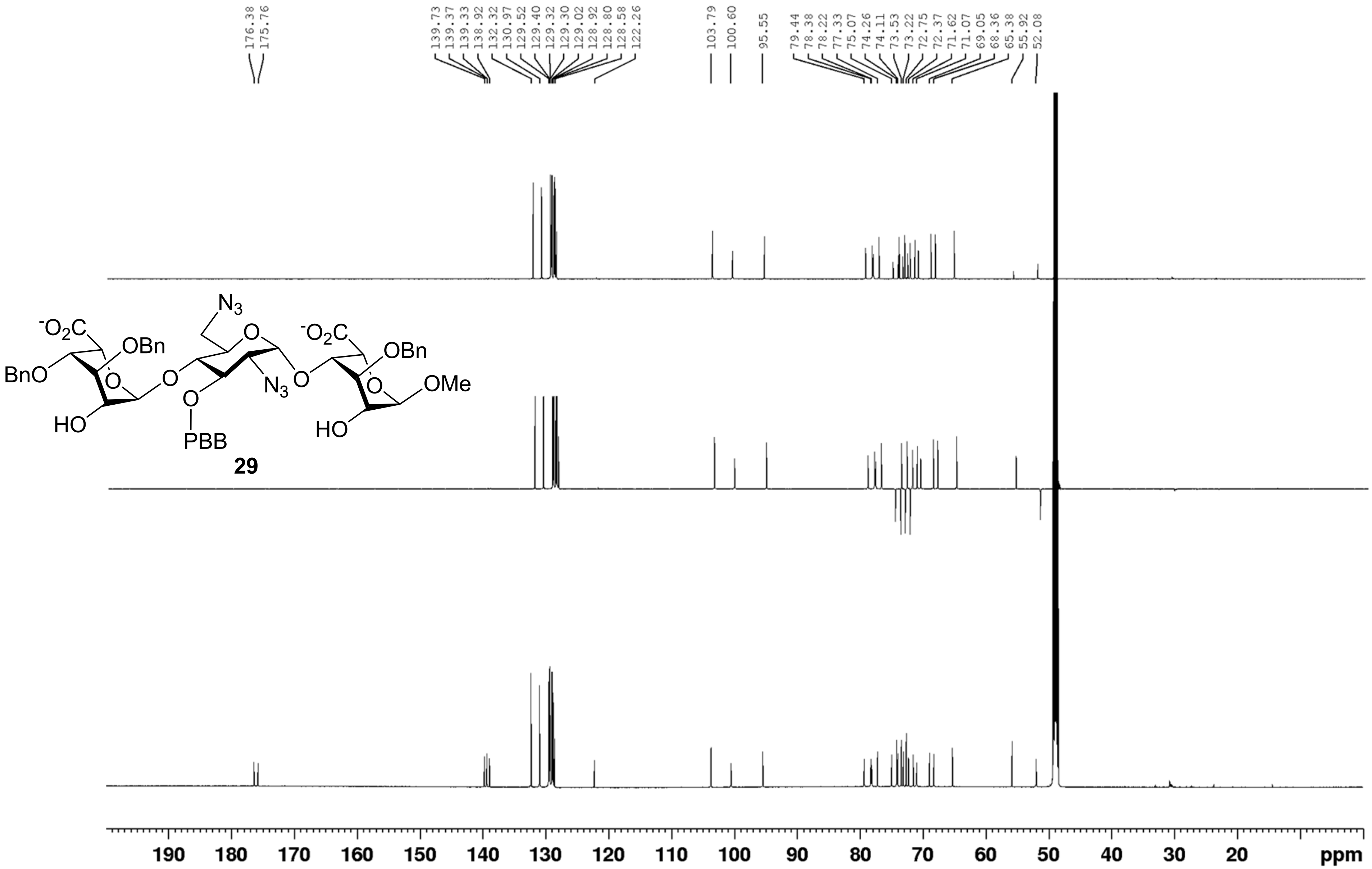

176.38
175.76
139.73
139.37
139.33
138.92
132.32
130.97
129.52
129.40
129.32
129.30
129.02
128.92
128.80
128.58
122.26
103.79
100.60
95.55
79.44
78.38
78.22
77.33
75.07
74.26
74.11
73.53
73.22
72.75
72.37
71.62
71.07
69.05
68.36
65.38
55.92
52.08
N3
-O2C
OBn
BnO
O
HO
PBB
N3
OMe
29
190
180
170
160
150
140
130
120
110
100
90
80
70
60
50
40
30
20
ppm

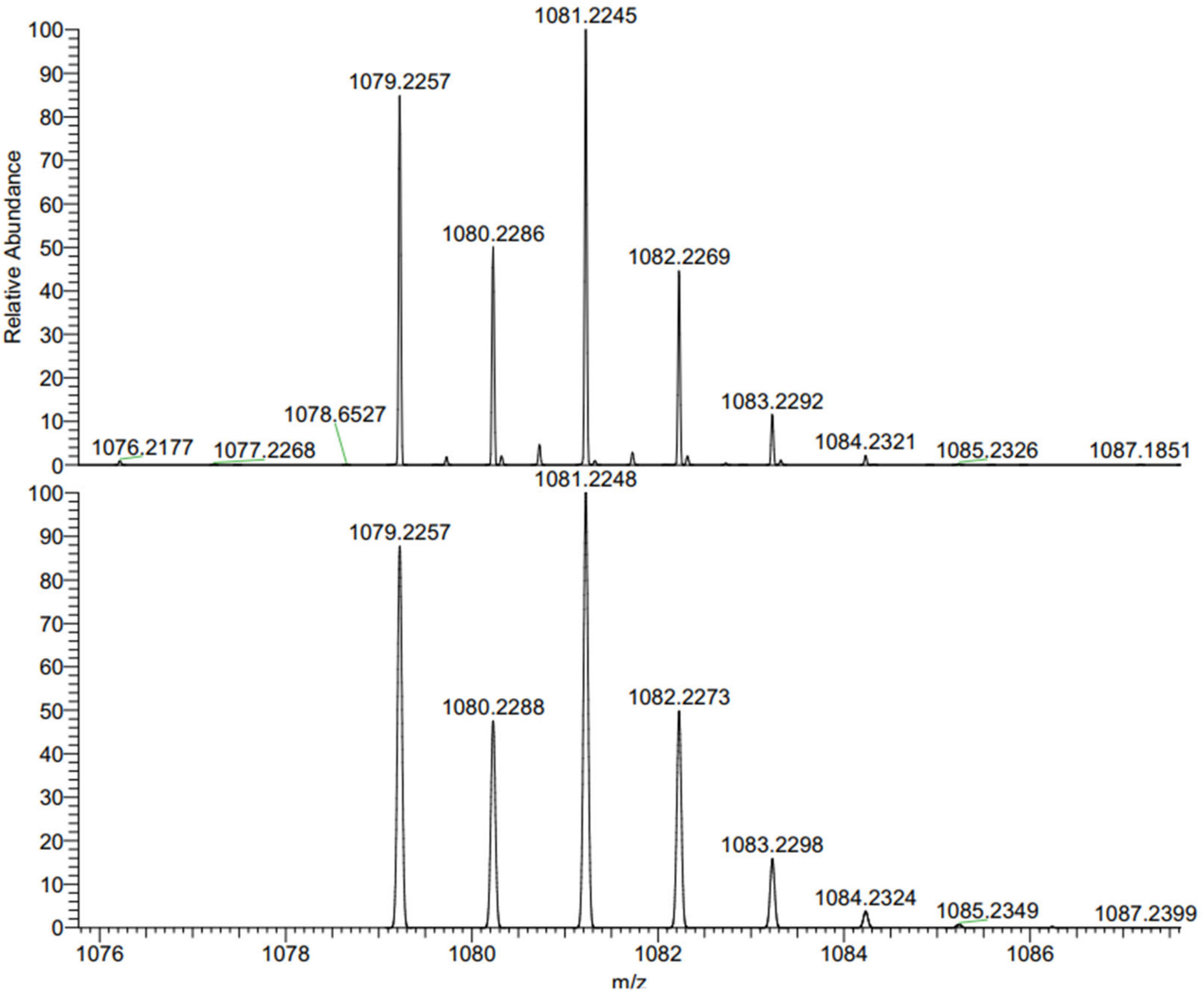

NL:
1.32E5
KYT-2054#24-32 RT:
0.69-0.85 AV: 9 T: FTMS + p
ESI Full ms [200.00-2000.00]

NL:
7.65E3
$C_{47}H_{49}BrN_6Na_2O_{16}$ +H:
$C_{47}H_{50}Br_1N_6Na_2O_{16}$
p (gss, s /p:40) Chrg 1
R: 20000 Res .Pwr . @FWHM

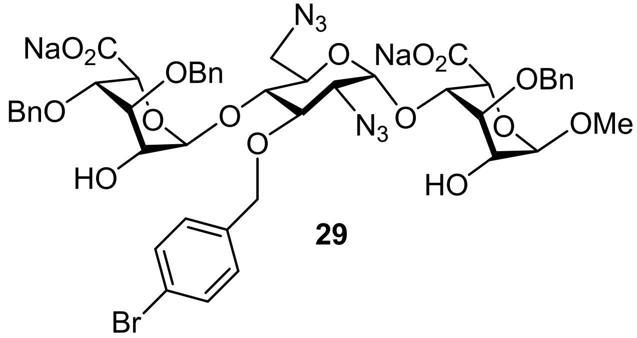

29

Chemical Formula: $C_{47}H_{49}BrN_6Na_2O_{16}$
Exact Mass: 1078.2184
Molecular Weight: 1079.8185

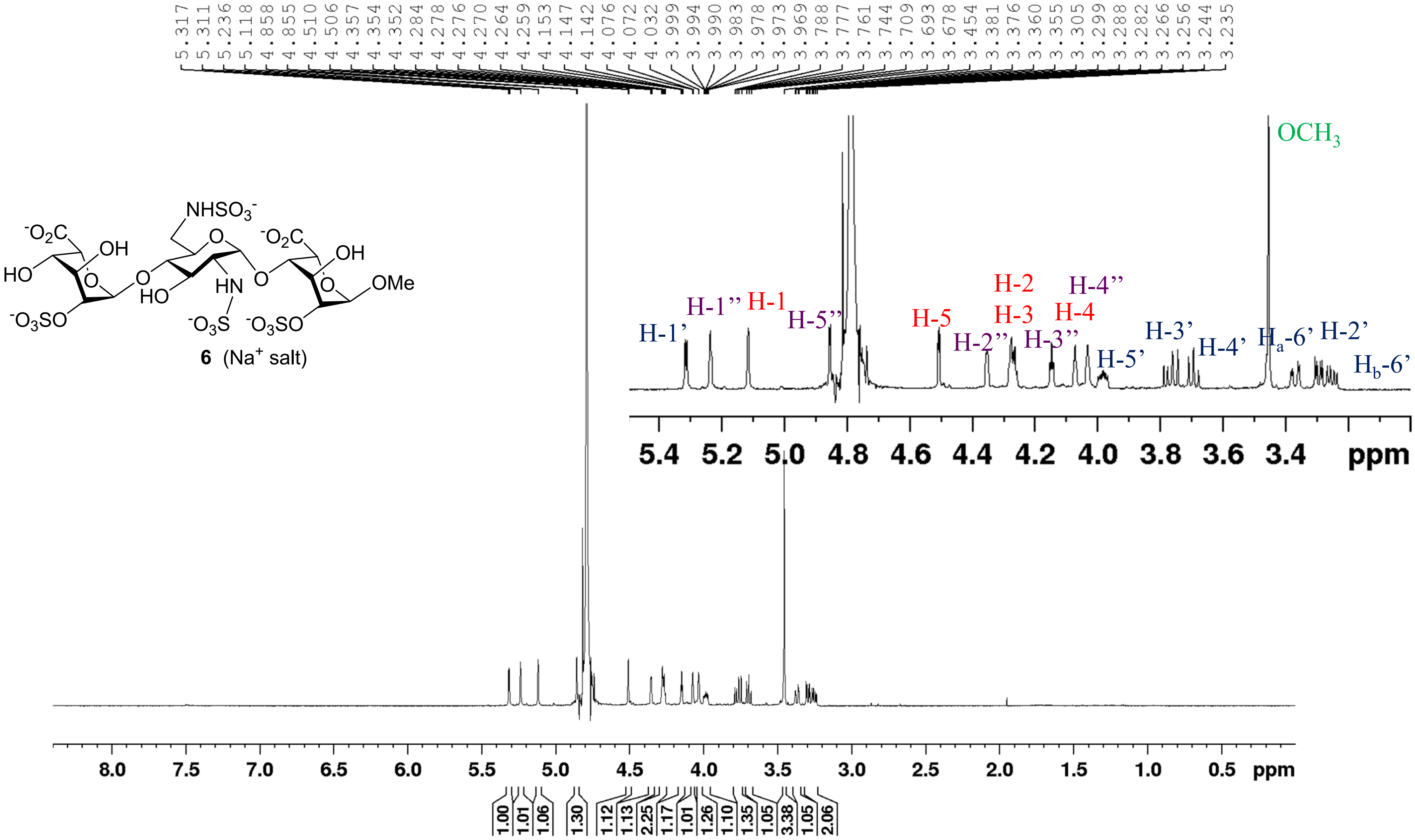

NHSO3-
-O2C
OH
HO
O
O
HO
HN
-O3S
O
-O2C
OH
O
OMe
-O3SO
-O3SO

6 (Na+ salt)

5.317
5.311
5.236
5.118
4.858
4.855
4.510
4.506
4.357
4.354
4.352
4.284
4.278
4.276
4.270
4.264
4.259
4.153
4.147
4.142
4.076
4.072
4.032
3.999
3.994
3.990
3.983
3.978
3.973
3.969
3.788
3.777
3.761
3.744
3.709
3.693
3.678
3.454
3.381
3.376
3.360
3.355
3.305
3.299
3.288
3.282
3.266
3.256
3.244
3.235
OCH3
H-1'
H-1''
H-1
H-5''
H-5
H-2''
H-2
H-3
H-3''
H-4
H-4''
H-5'
H-3'
H-4'
Ha-6'
H-2'
Hb-6'
5.4
5.2
5.0
4.8
4.6
4.4
4.2
4.0
3.8
3.6
3.4
ppm
8.0
7.5
7.0
6.5
6.0
5.5
5.0
4.5
4.0
3.5
3.0
2.5
2.0
1.5
1.0
0.5
ppm
1.00
1.01
1.06
1.30
1.12
1.13
2.25
1.17
1.01
1.26
1.10
1.35
1.05
3.38
1.05
2.06

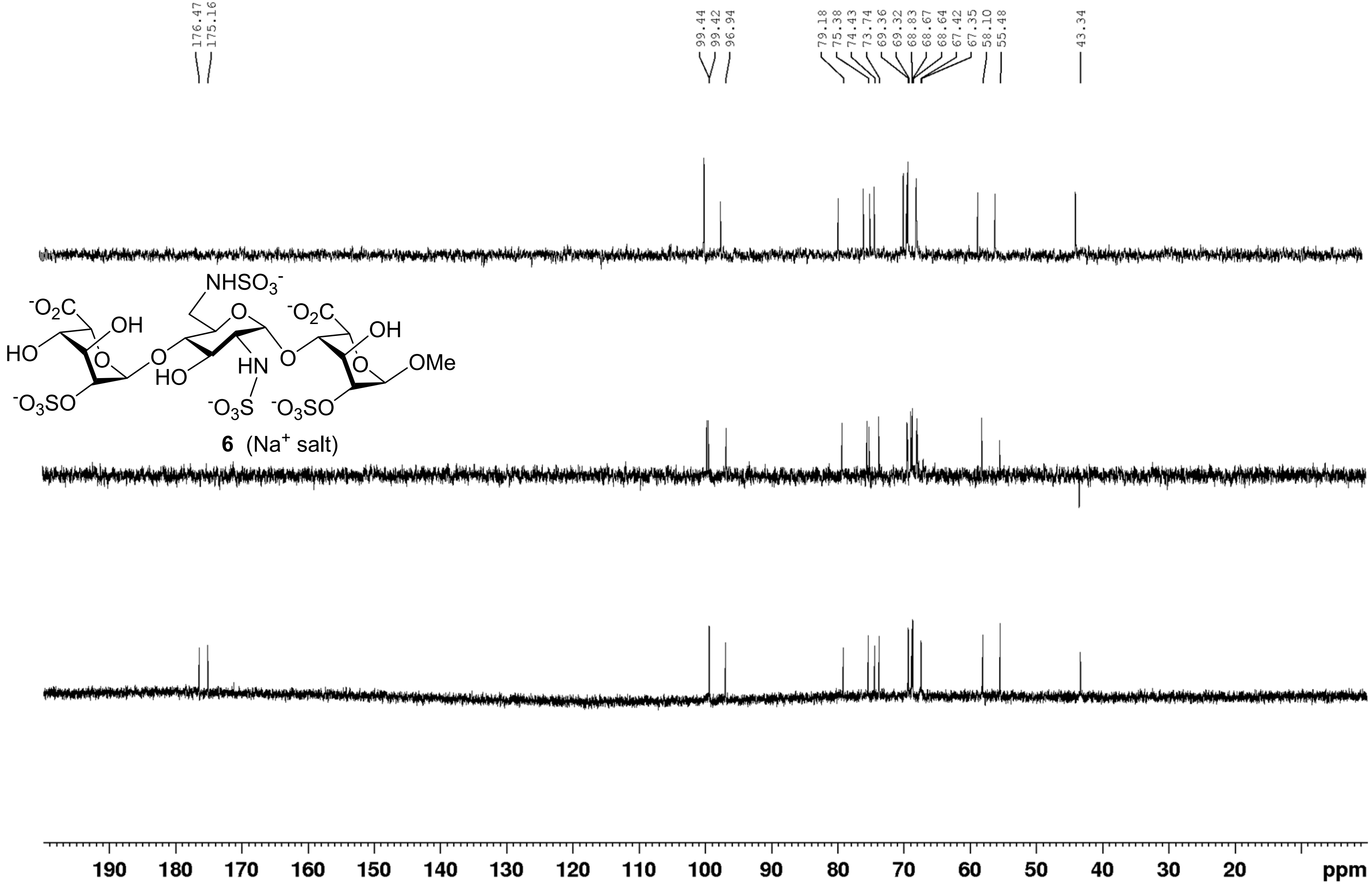
176.47
175.16
99.44
99.42
96.94
79.18
75.38
74.43
73.74
69.36
69.32
68.83
68.67
68.64
67.42
67.35
58.10
55.48
43.34
NHSO3-
-O2C
OH
HO
O
-O3SO
HO
HN
-O3S
OMe
6 (Na+ salt)
190 180 170 160 150 140 130 120 110 100 90 80 70 60 50 40 30 20 ppm

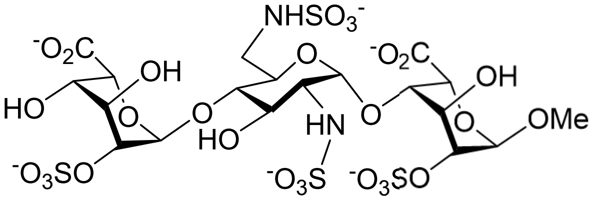

NHSO3-
-O2C
OH
HO
O
-O3SO
HO
HN
-O3S
OMe

6 (Na+ salt)

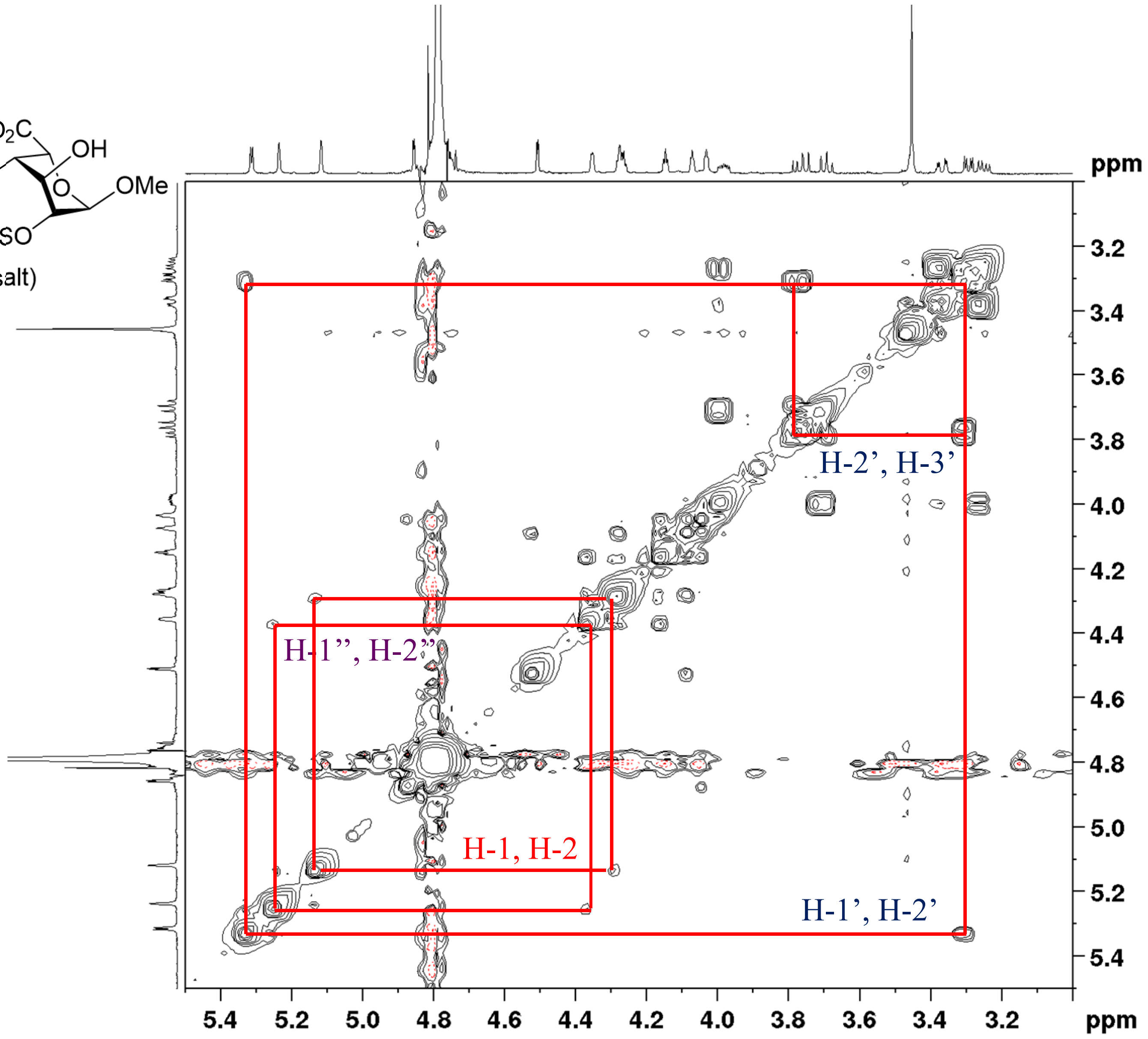

ppm
H-2’, H-3’
H-1”, H-2”
H-1, H-2
H-1’, H-2’
3.2
3.4
3.6
3.8
4.0
4.2
4.4
4.6
4.8
5.0
5.2
5.4

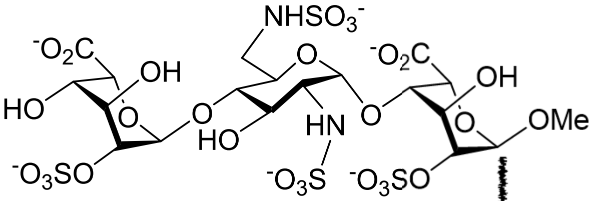
NHSO3-
-O2C
OH
HO
O
O
HN
O
OMe
-O3SO
HO
-O3S
-O3SO

6 (Na+ salt)

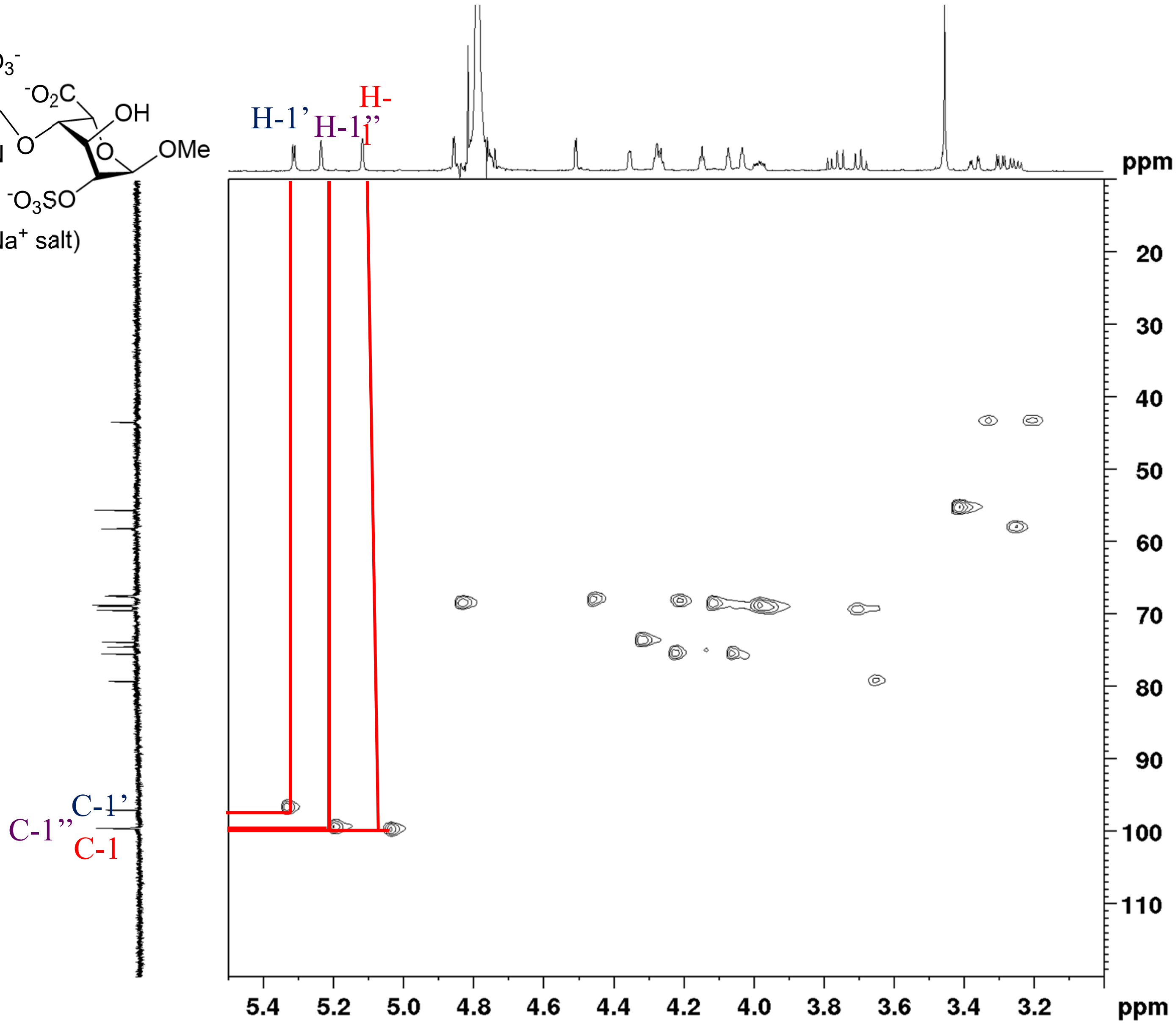
H-1'
H-1''
H-1
ppm
C-1'
C-1''
C-1
20
30
40
50
60
70
80
90
100
110
ppm
5.4
5.2
5.0
4.8
4.6
4.4
4.2
4.0
3.8
3.6
3.4
3.2
ppm

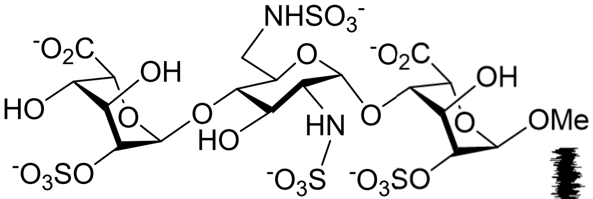

$^{-}O_2C$
OH
HO
O
$^{-}O_3SO$
$NHSO_3^{-}$
HO
HN
$^{-}O_3S$
$^{-}O_2C$
OH
$^{-}O_3SO$
OMe

6 (Na^{+} salt)

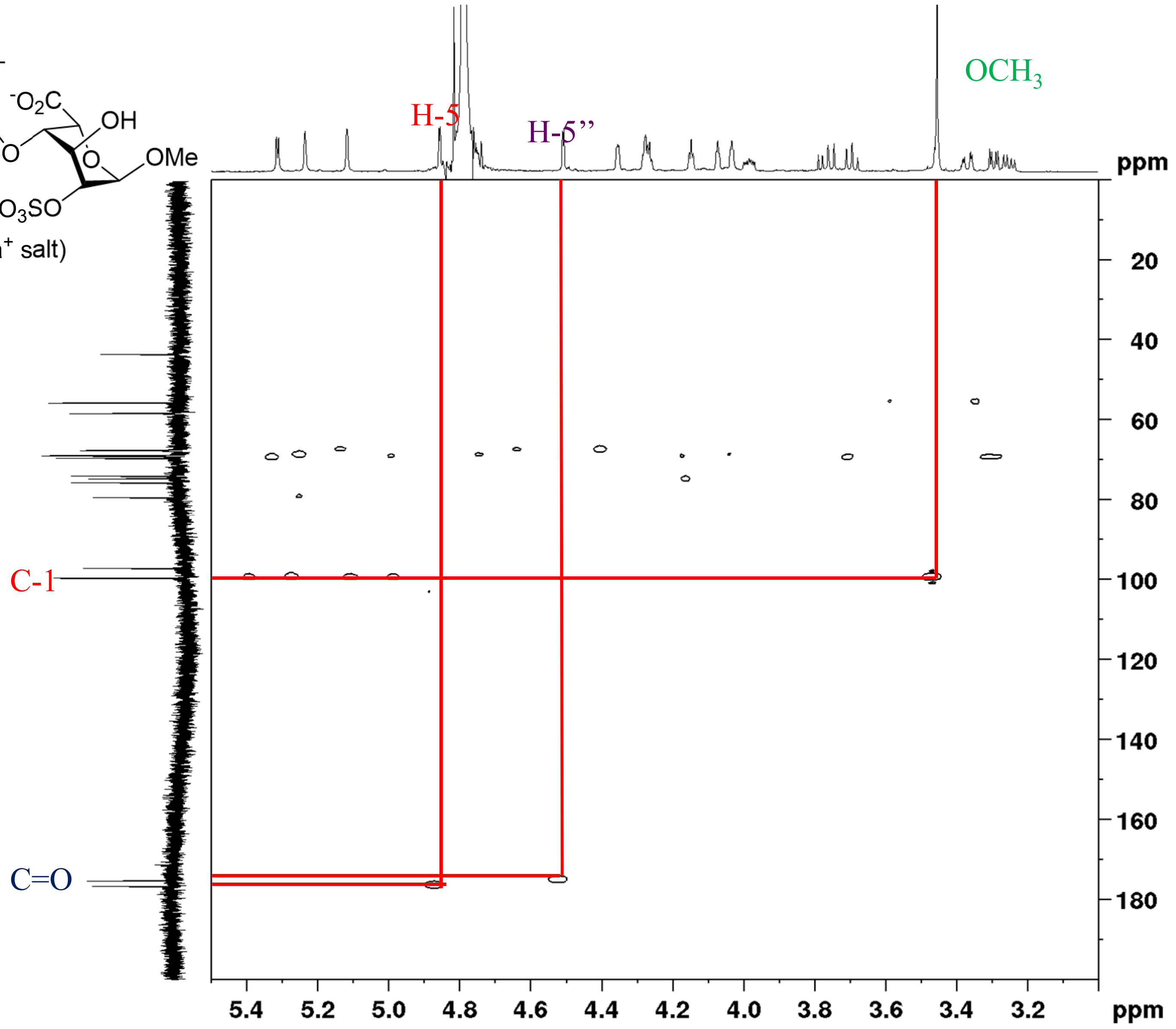

H-5
H-5''
OCH$_3$
ppm
C-1
C=O
20
40
60
80
100
120
140
160
180
5.4
5.2
5.0
4.8
4.6
4.4
4.2
4.0
3.8
3.6
3.4
3.2
ppm

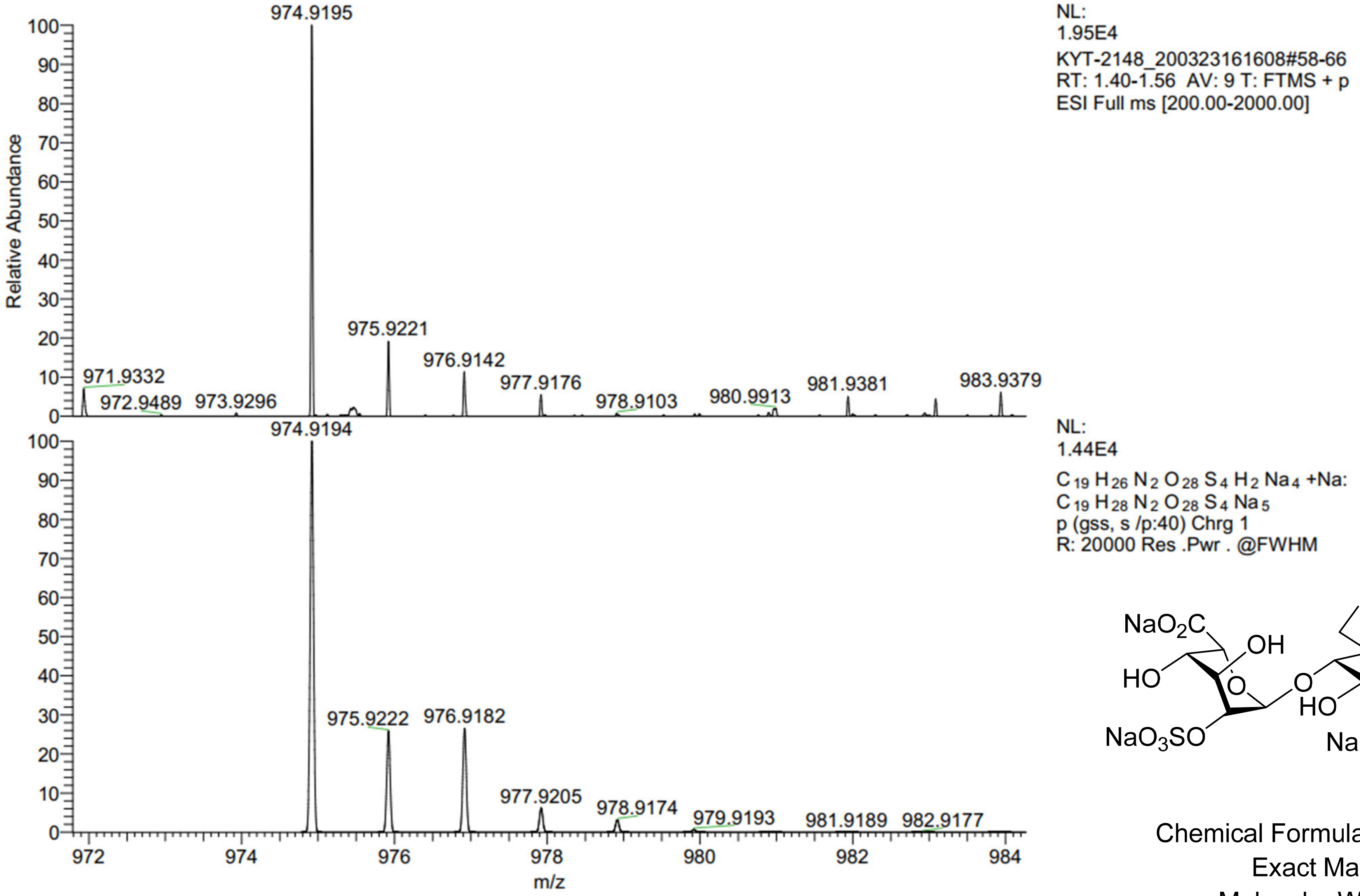

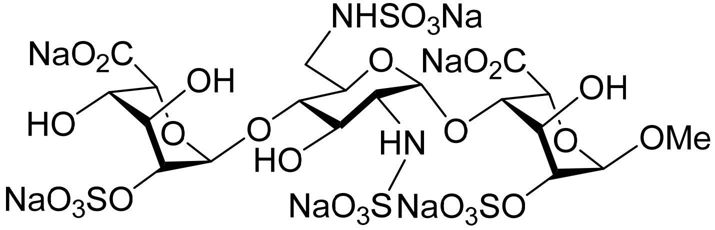

6 (Na^+ salt)

Chemical Formula: $C_{19}H_{26}N_2Na_6O_{28}S_4$

Exact Mass: 995.8941

Molecular Weight: 996.5816
